# Supplementary figures and images for: Urdu text in natural scene images: a new dataset and preliminary text detection
Source: PeerJ Comput Sci. 2021 Sep 16;7:e717. doi: 10.7717/peerj-cs.717 (PMC8459794; doi:10.7717/peerj-cs.717)

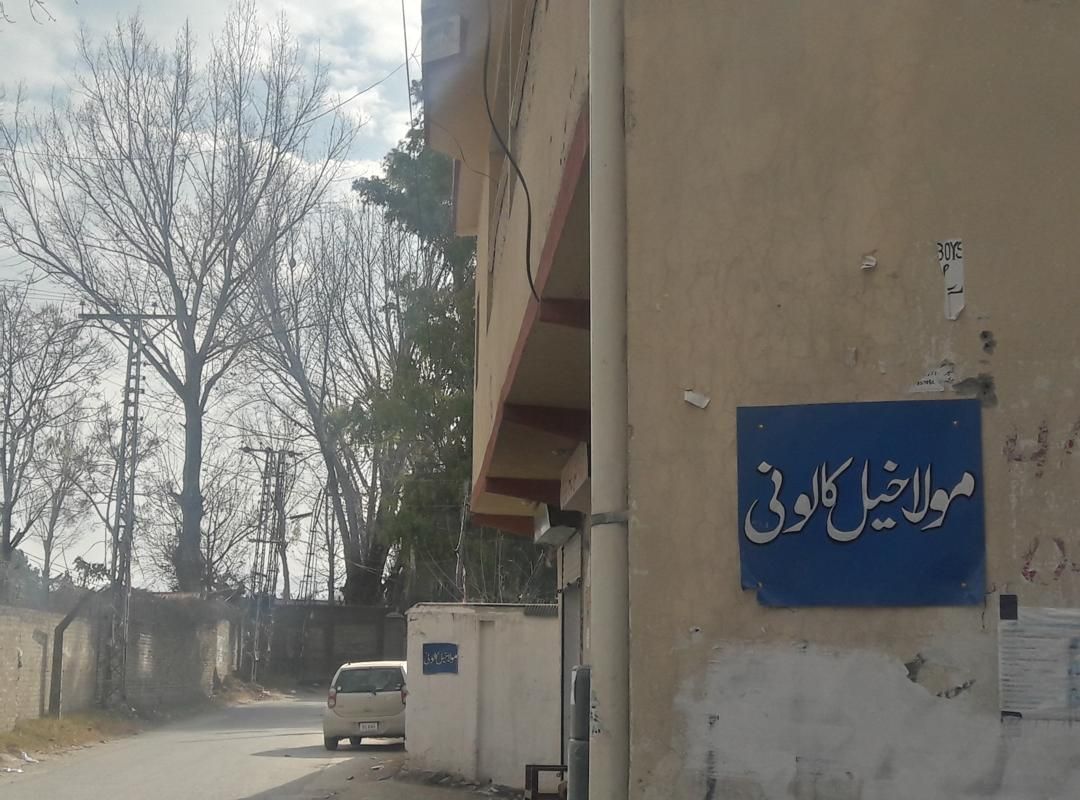

Supplement: Supplemental Information 2 [file peerj-cs-07-717-s002.zip › Testing Dataset/01.jpg]

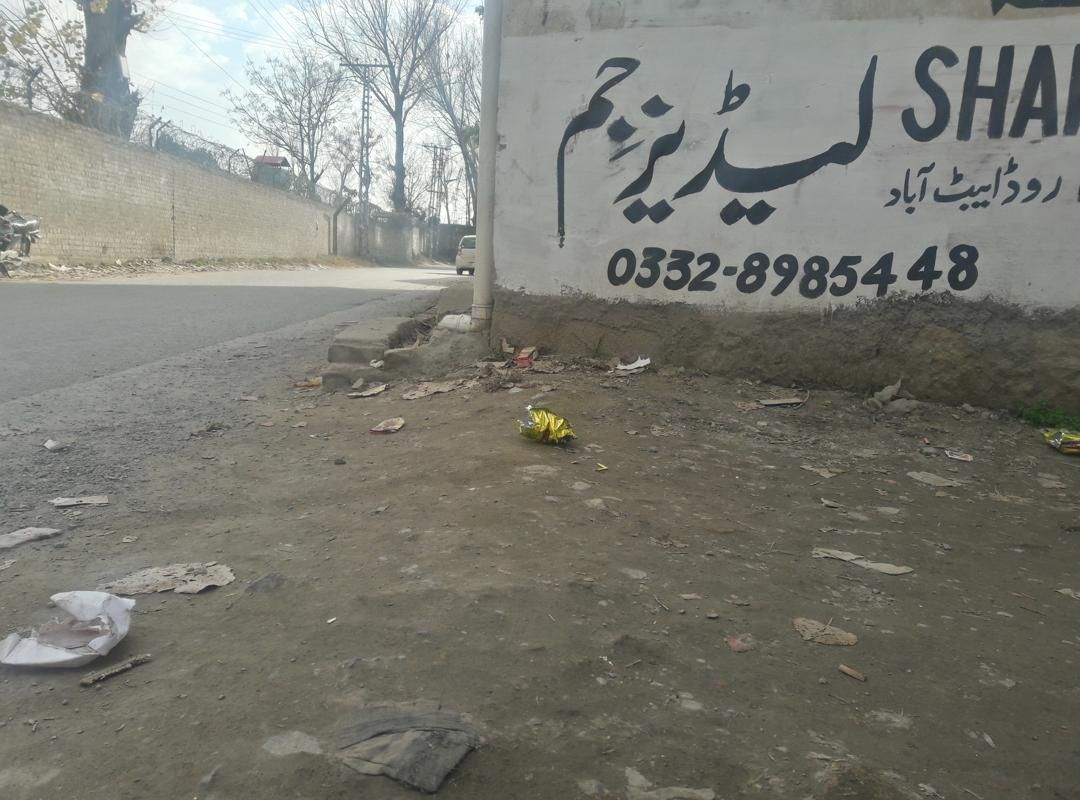

Supplement: Supplemental Information 2 [file peerj-cs-07-717-s002.zip › Testing Dataset/02.jpg]

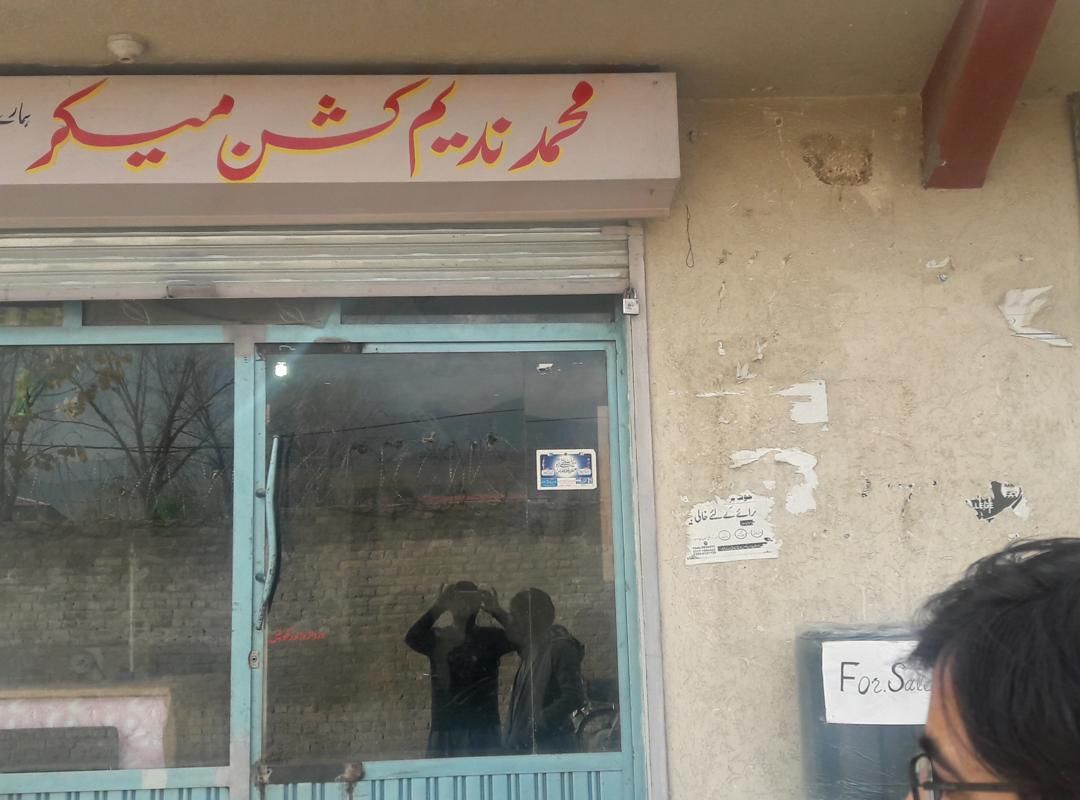

Supplement: Supplemental Information 2 [file peerj-cs-07-717-s002.zip › Testing Dataset/03.jpg]

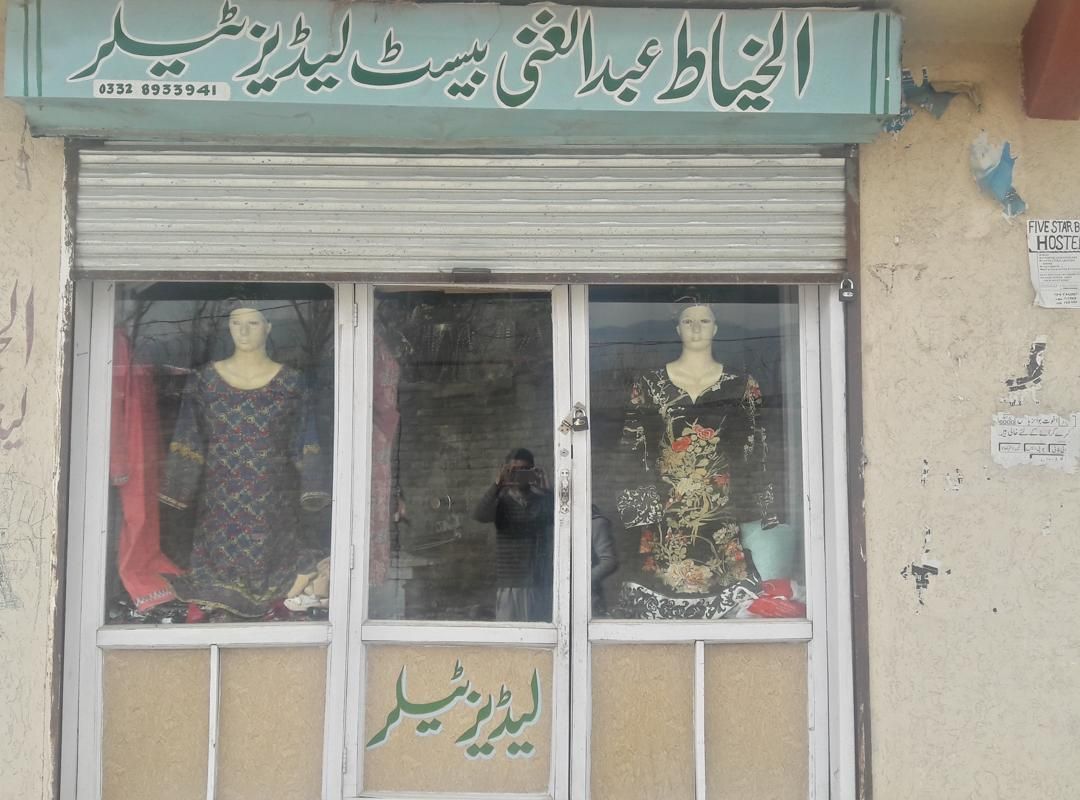

Supplement: Supplemental Information 2 [file peerj-cs-07-717-s002.zip › Testing Dataset/04.jpg]

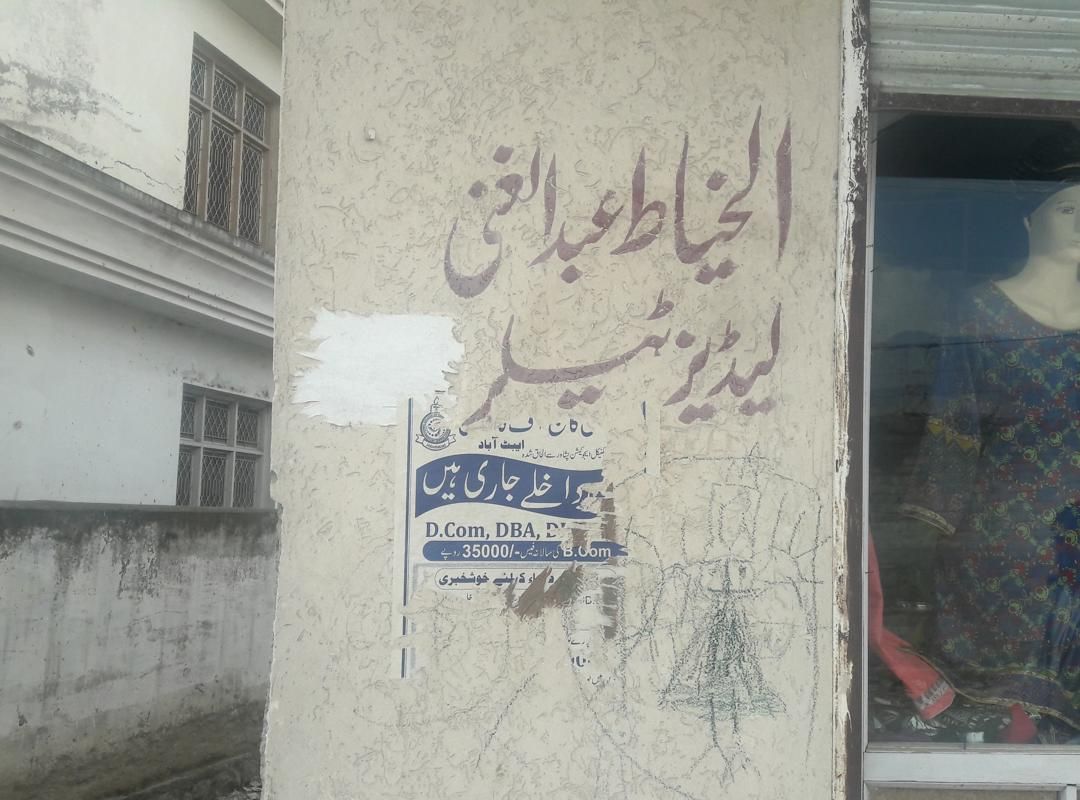

Supplement: Supplemental Information 2 [file peerj-cs-07-717-s002.zip › Testing Dataset/05.jpg]

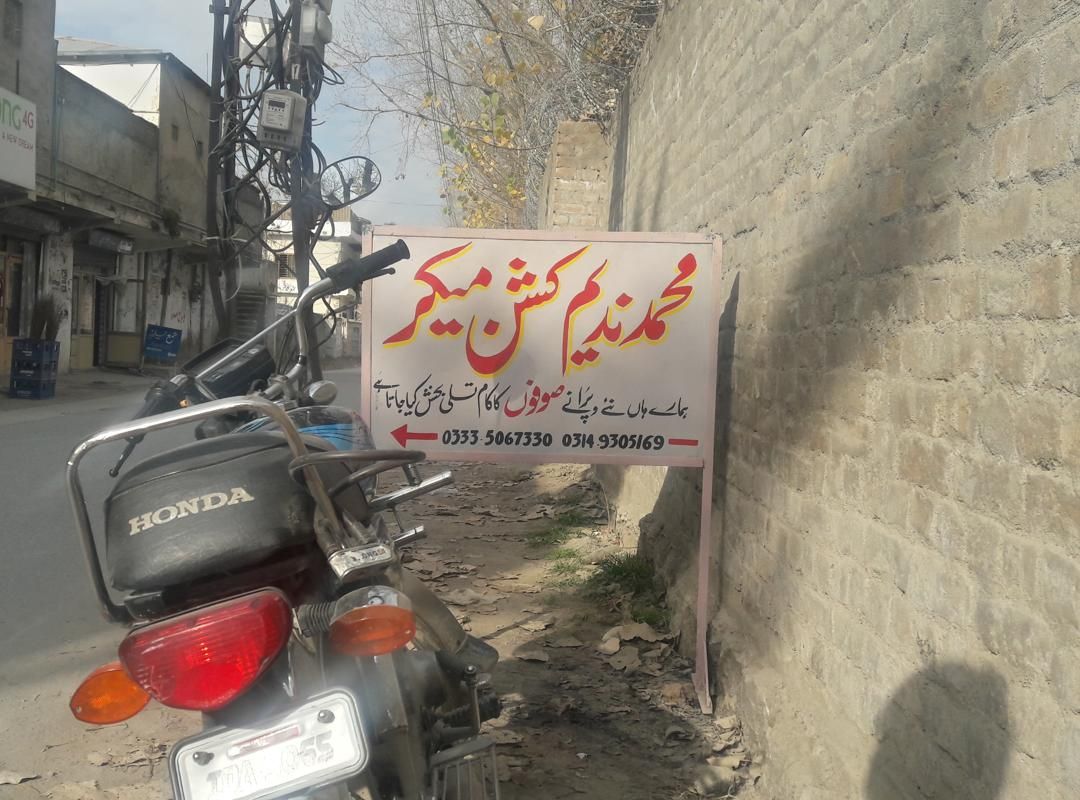

Supplement: Supplemental Information 2 [file peerj-cs-07-717-s002.zip › Testing Dataset/06.jpg]

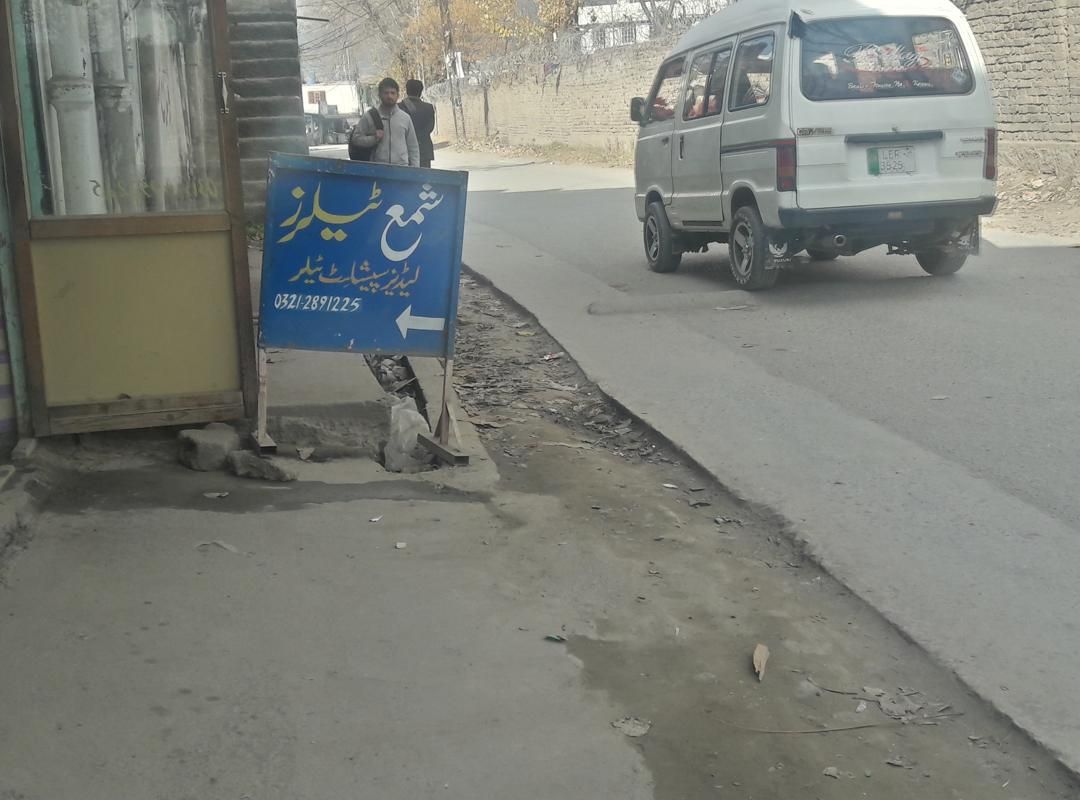

Supplement: Supplemental Information 2 [file peerj-cs-07-717-s002.zip › Testing Dataset/07.jpg]

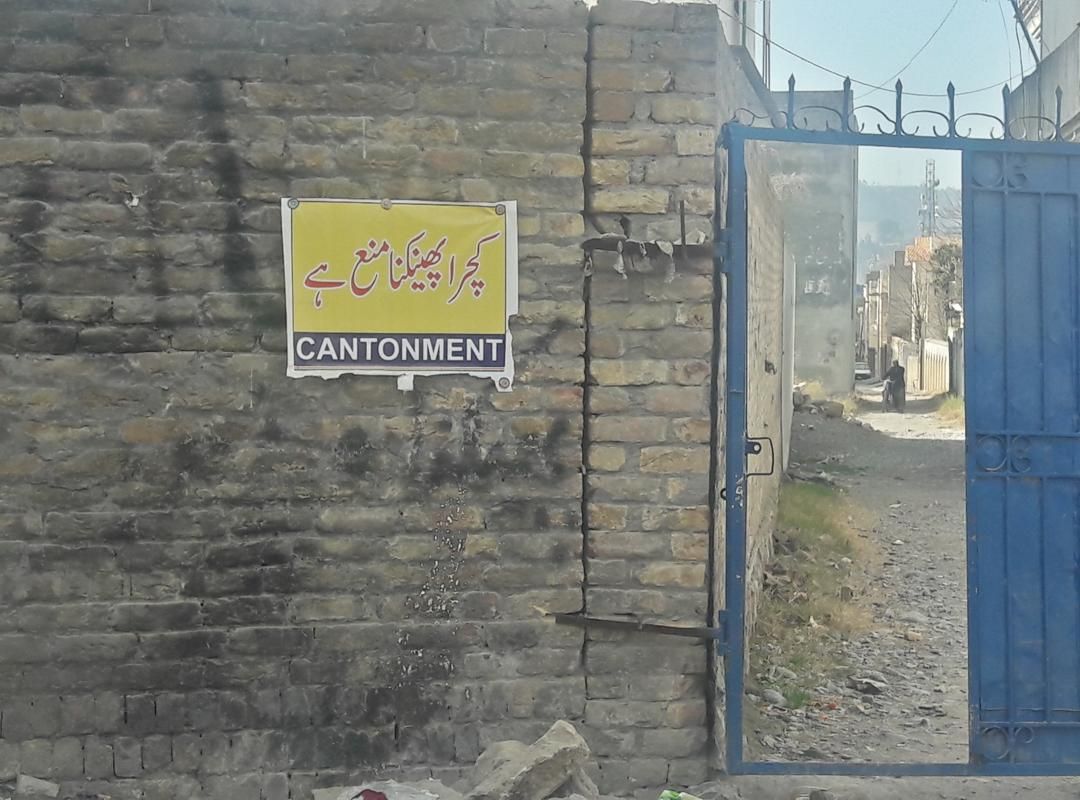

Supplement: Supplemental Information 2 [file peerj-cs-07-717-s002.zip › Testing Dataset/08.jpg]

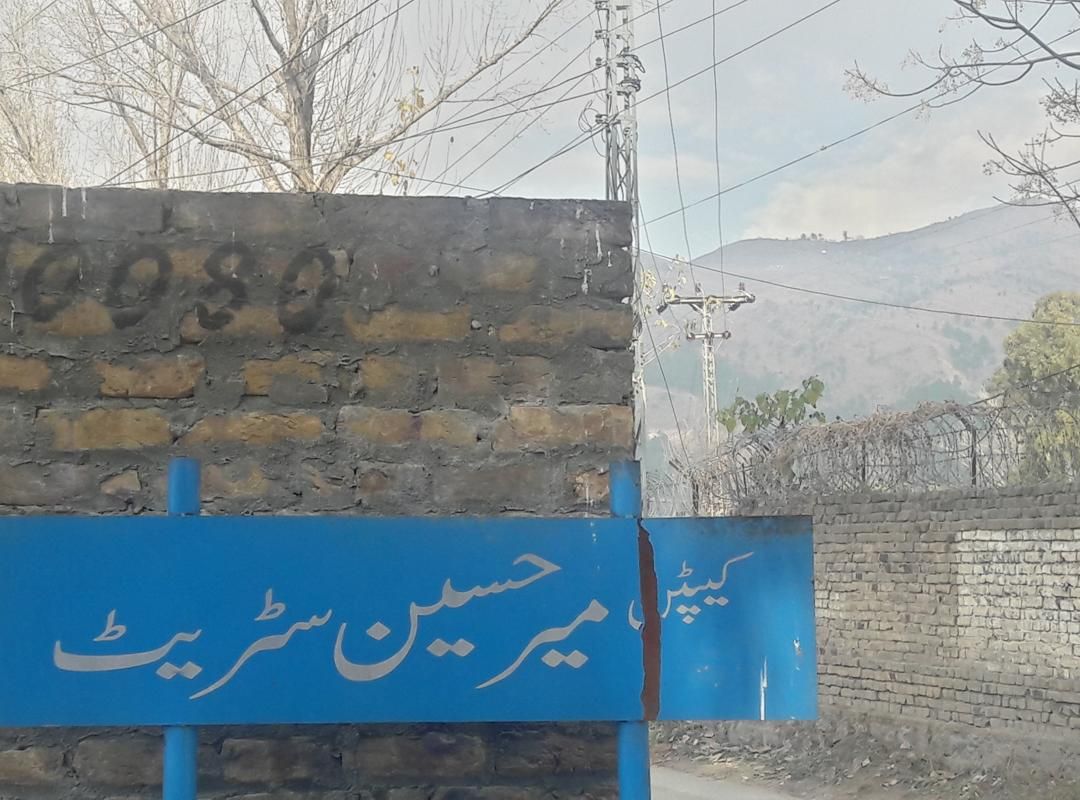

Supplement: Supplemental Information 2 [file peerj-cs-07-717-s002.zip › Testing Dataset/09.jpg]

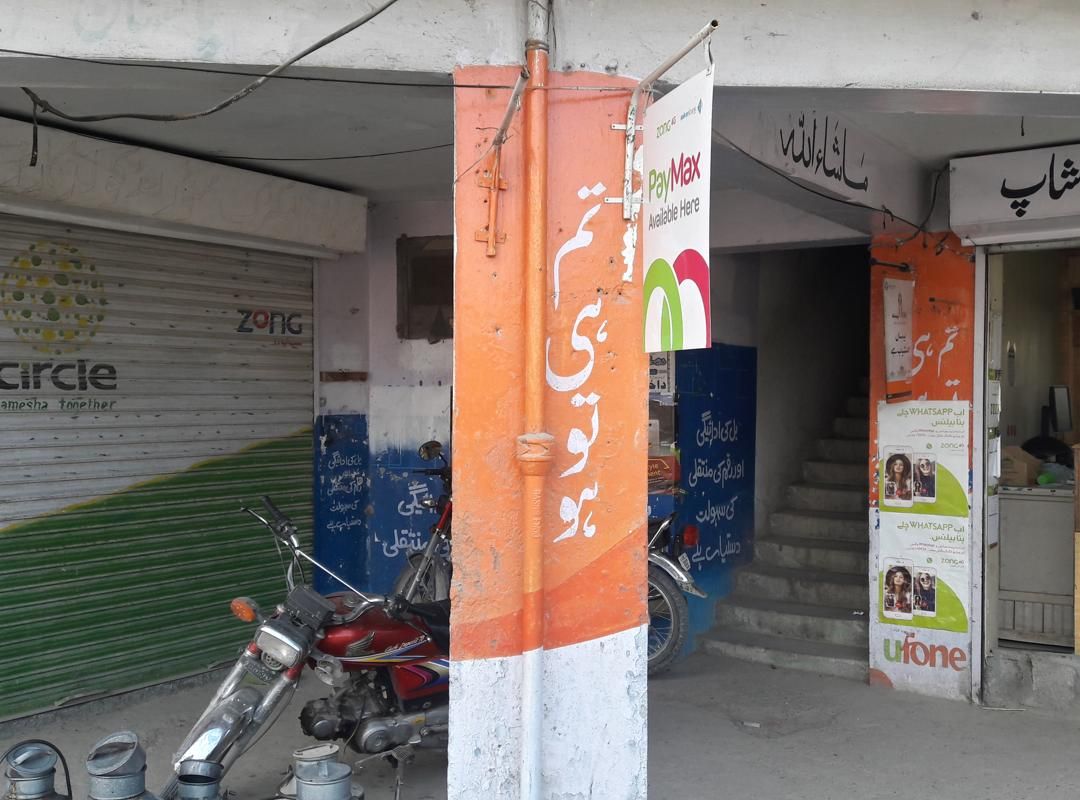

Supplement: Supplemental Information 2 [file peerj-cs-07-717-s002.zip › Testing Dataset/10.jpg]

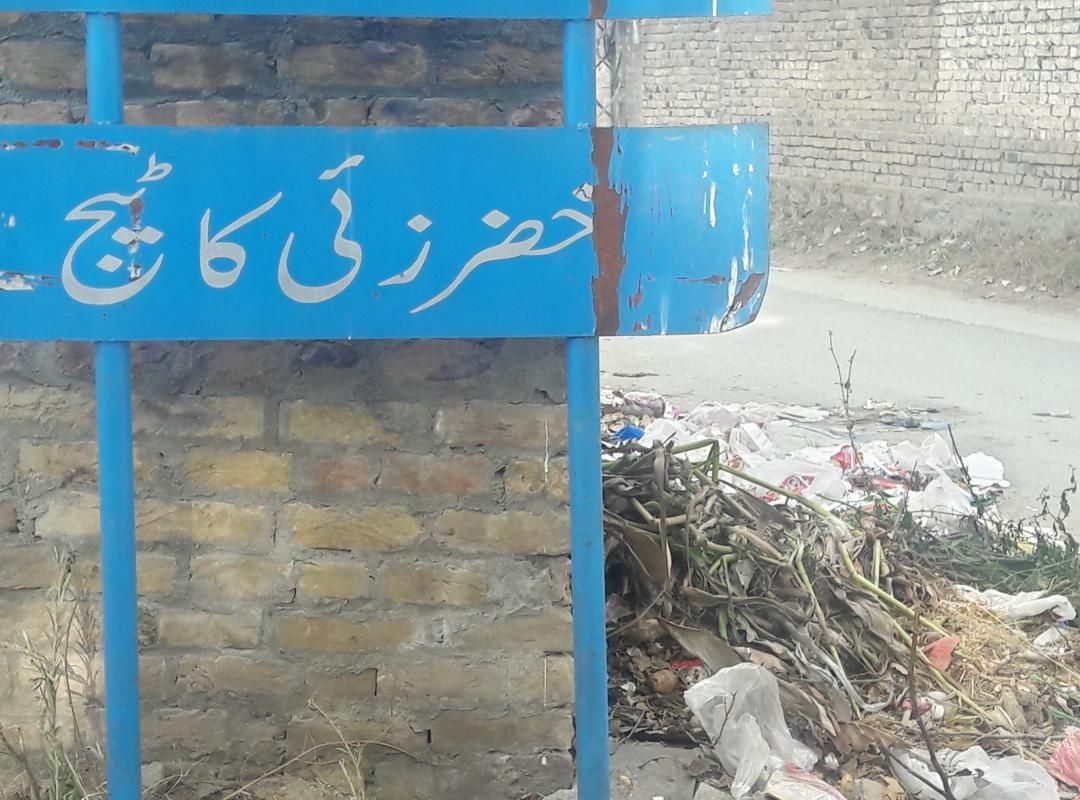

Supplement: Supplemental Information 2 [file peerj-cs-07-717-s002.zip › Testing Dataset/11.jpg]

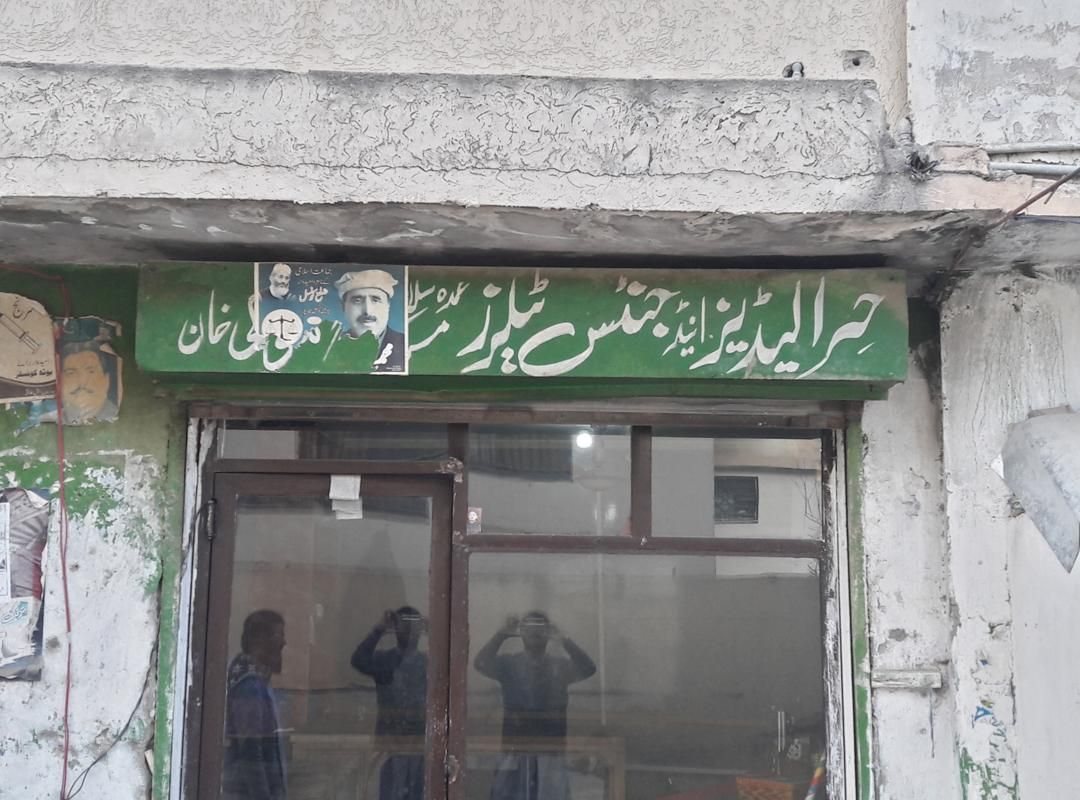

Supplement: Supplemental Information 2 [file peerj-cs-07-717-s002.zip › Testing Dataset/12.jpg]

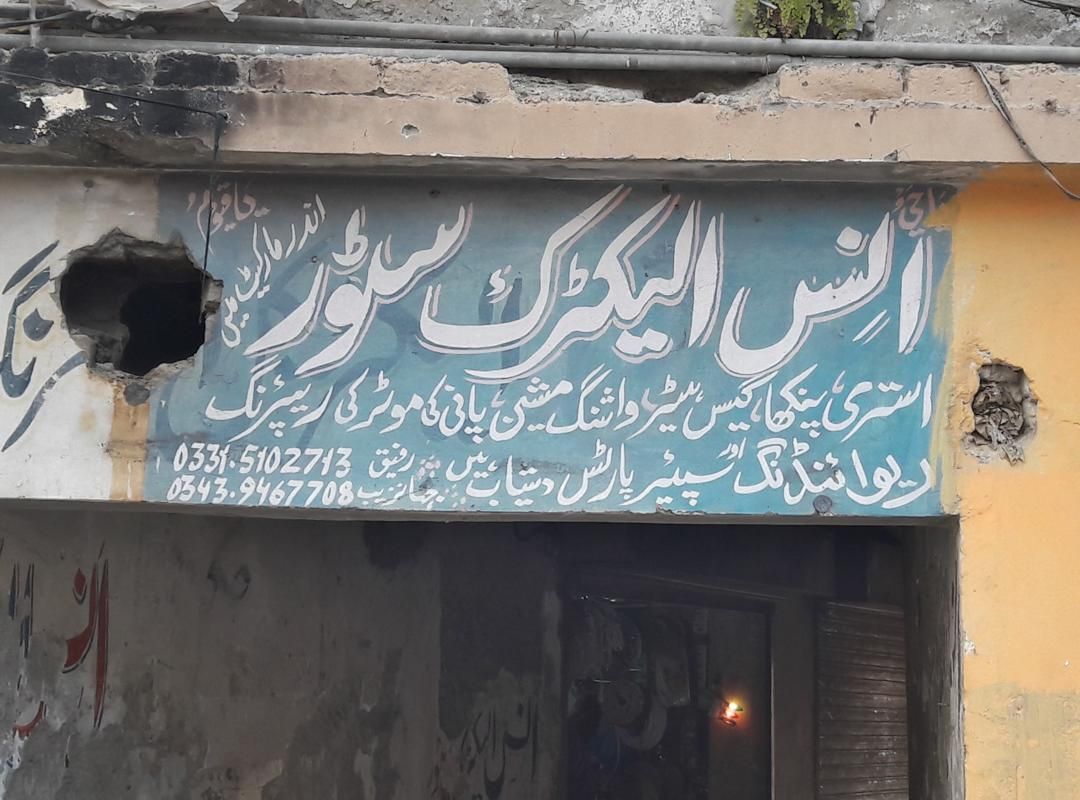

Supplement: Supplemental Information 2 [file peerj-cs-07-717-s002.zip › Testing Dataset/13.jpg]

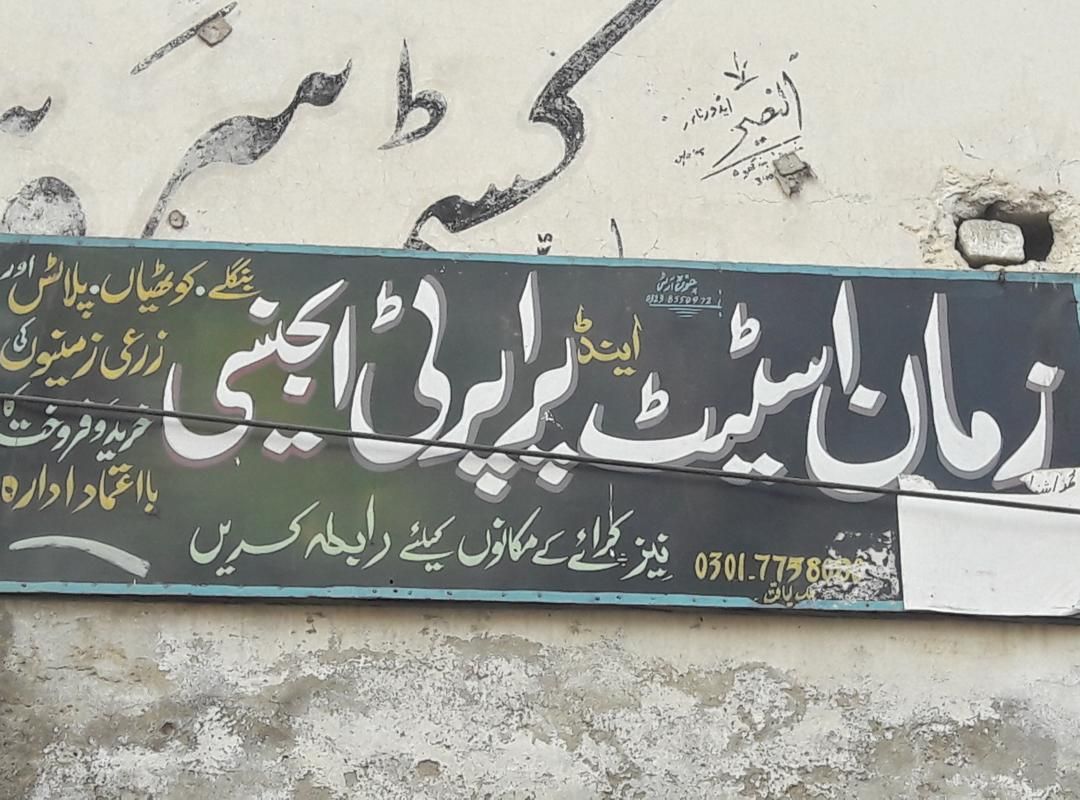

Supplement: Supplemental Information 2 [file peerj-cs-07-717-s002.zip › Testing Dataset/14.jpg]

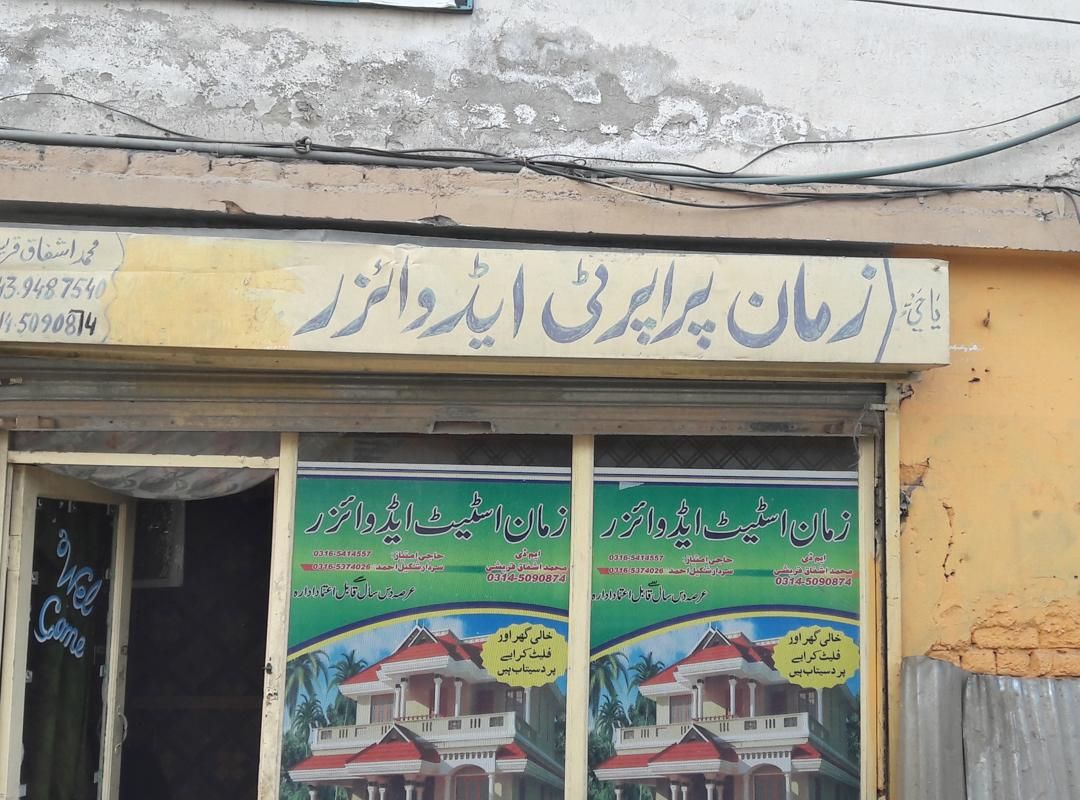

Supplement: Supplemental Information 2 [file peerj-cs-07-717-s002.zip › Testing Dataset/15.jpg]

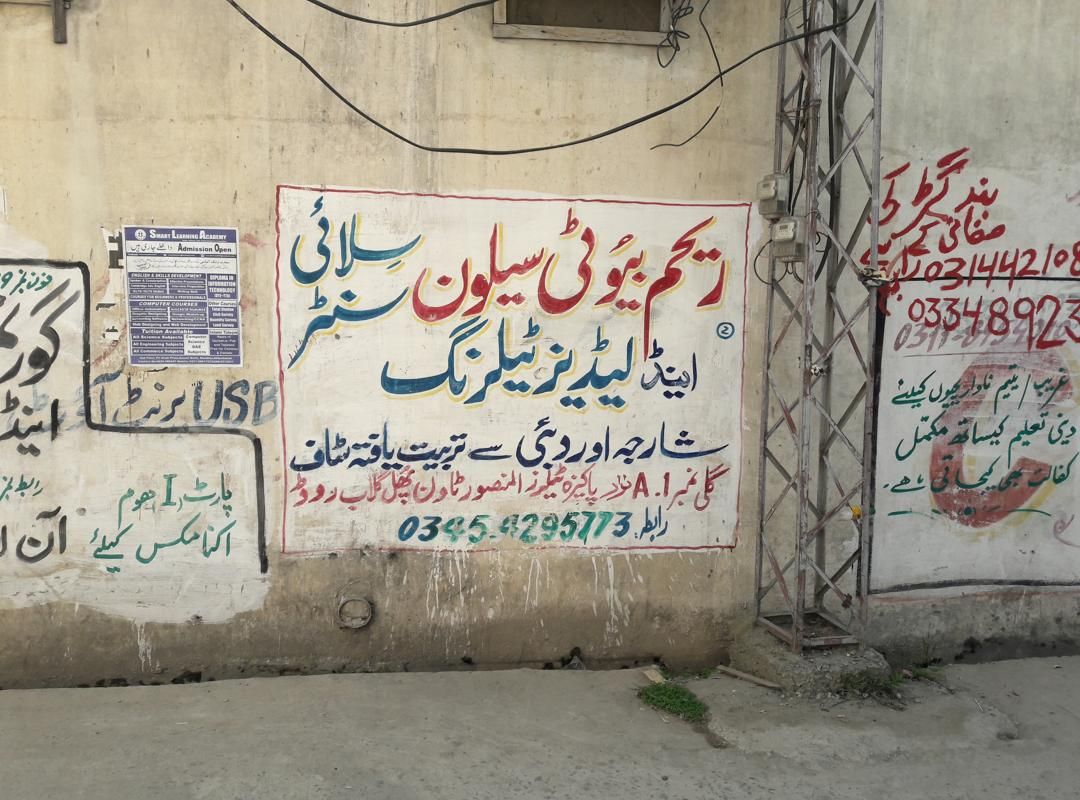

Supplement: Supplemental Information 2 [file peerj-cs-07-717-s002.zip › Testing Dataset/16.jpg]

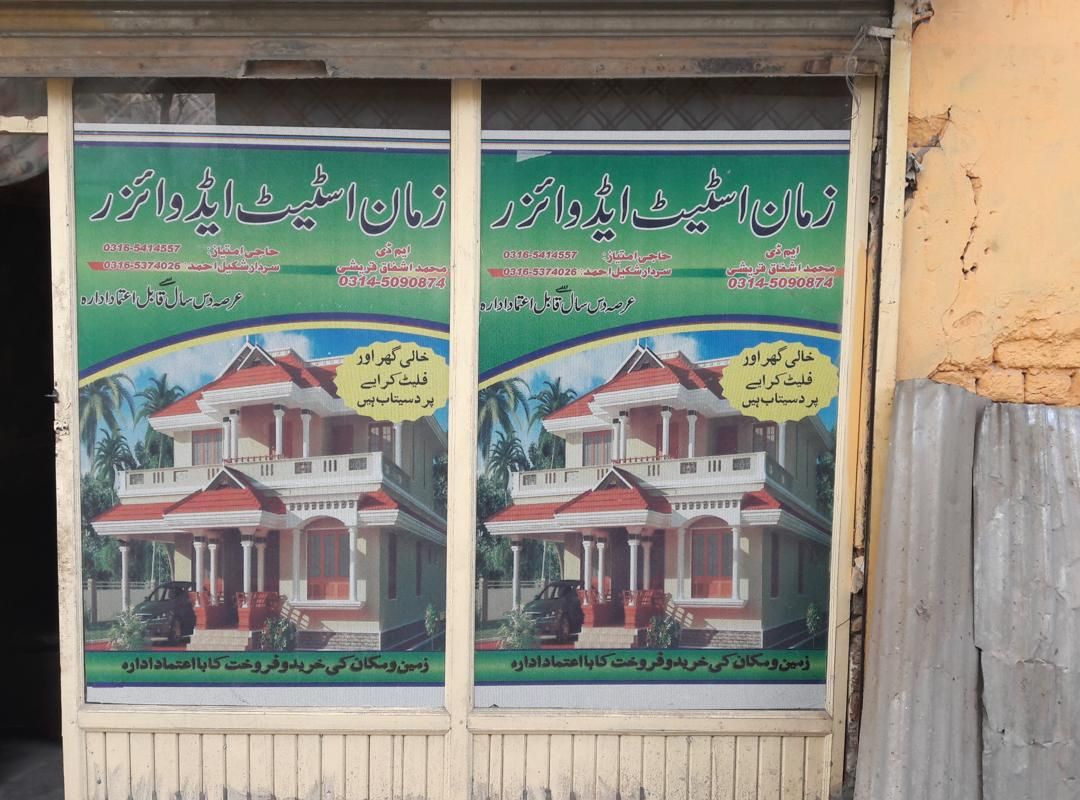

Supplement: Supplemental Information 2 [file peerj-cs-07-717-s002.zip › Testing Dataset/17.jpg]

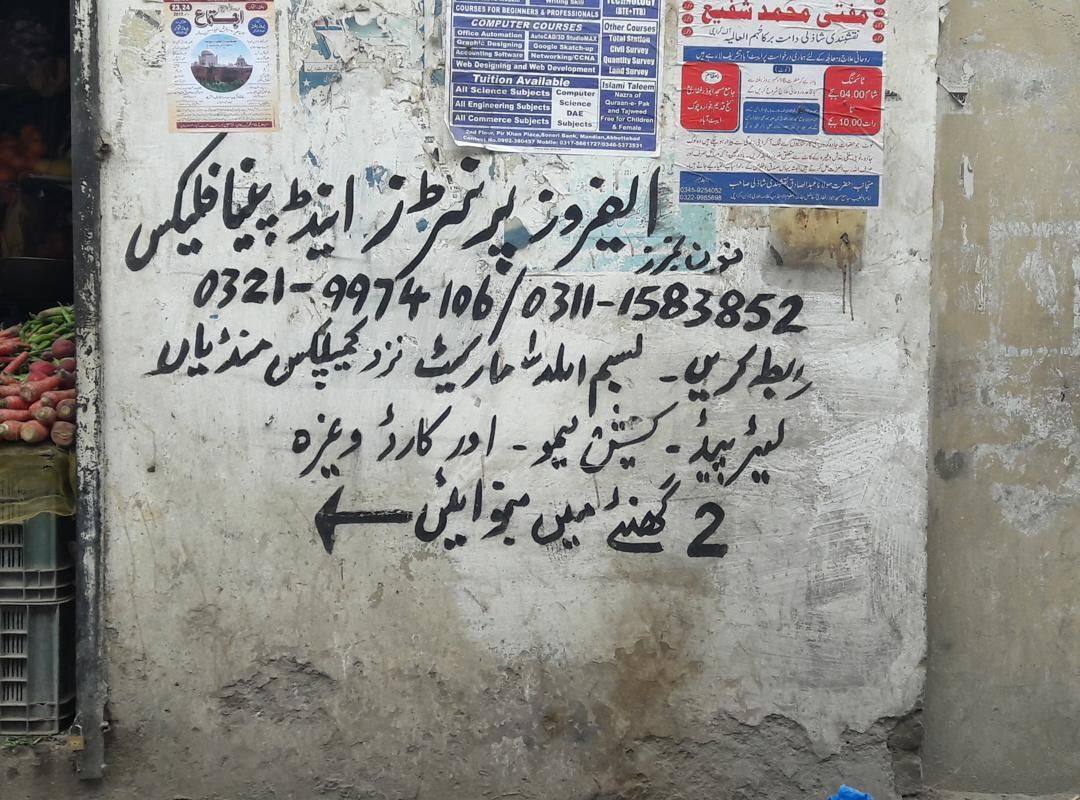

Supplement: Supplemental Information 2 [file peerj-cs-07-717-s002.zip › Testing Dataset/18.jpg]

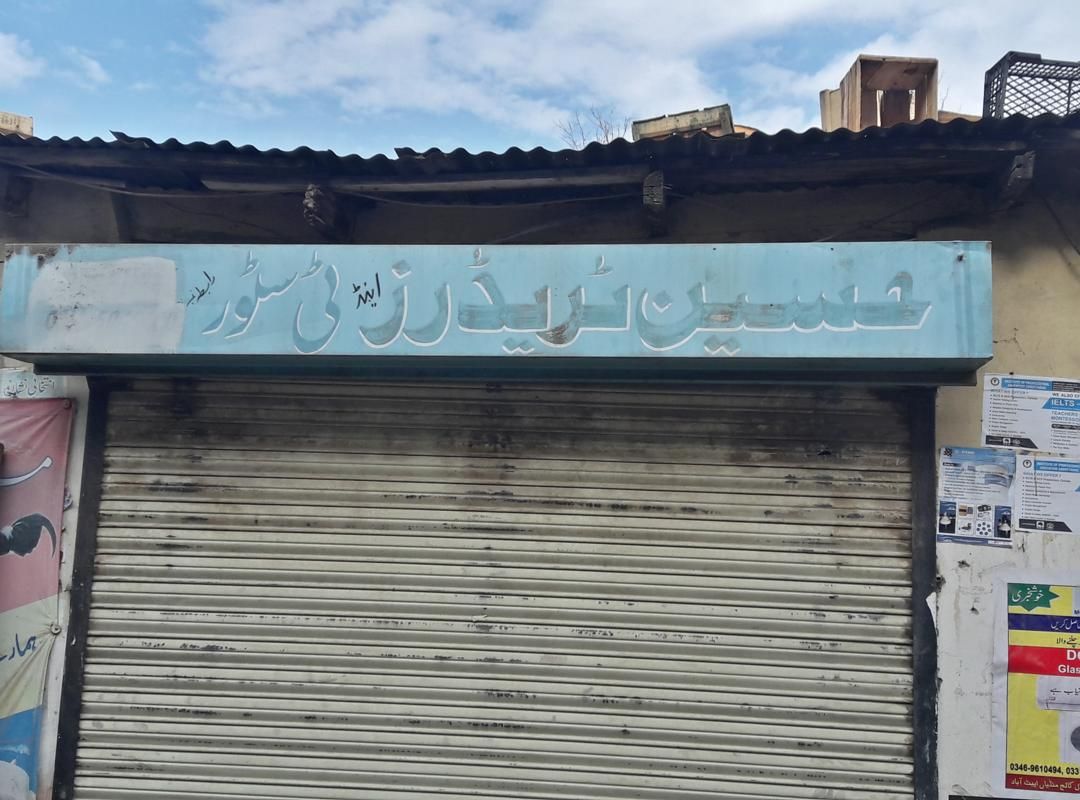

Supplement: Supplemental Information 2 [file peerj-cs-07-717-s002.zip › Testing Dataset/19.jpg]

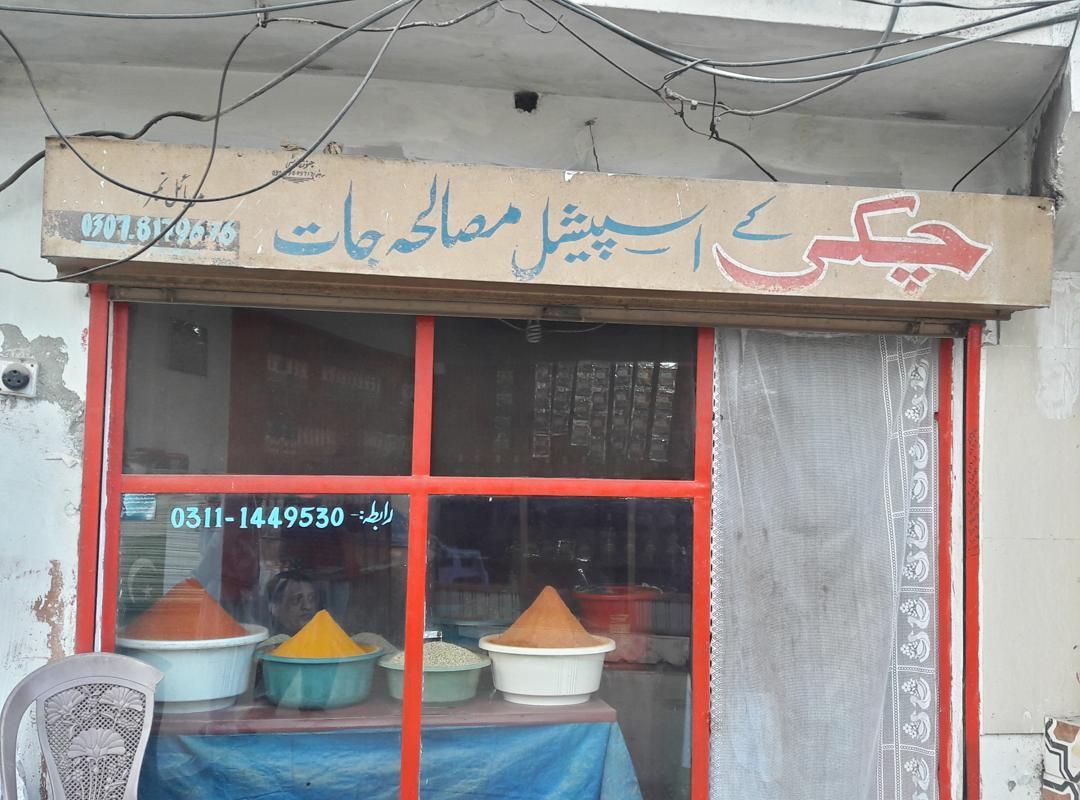

Supplement: Supplemental Information 2 [file peerj-cs-07-717-s002.zip › Testing Dataset/20.jpg]

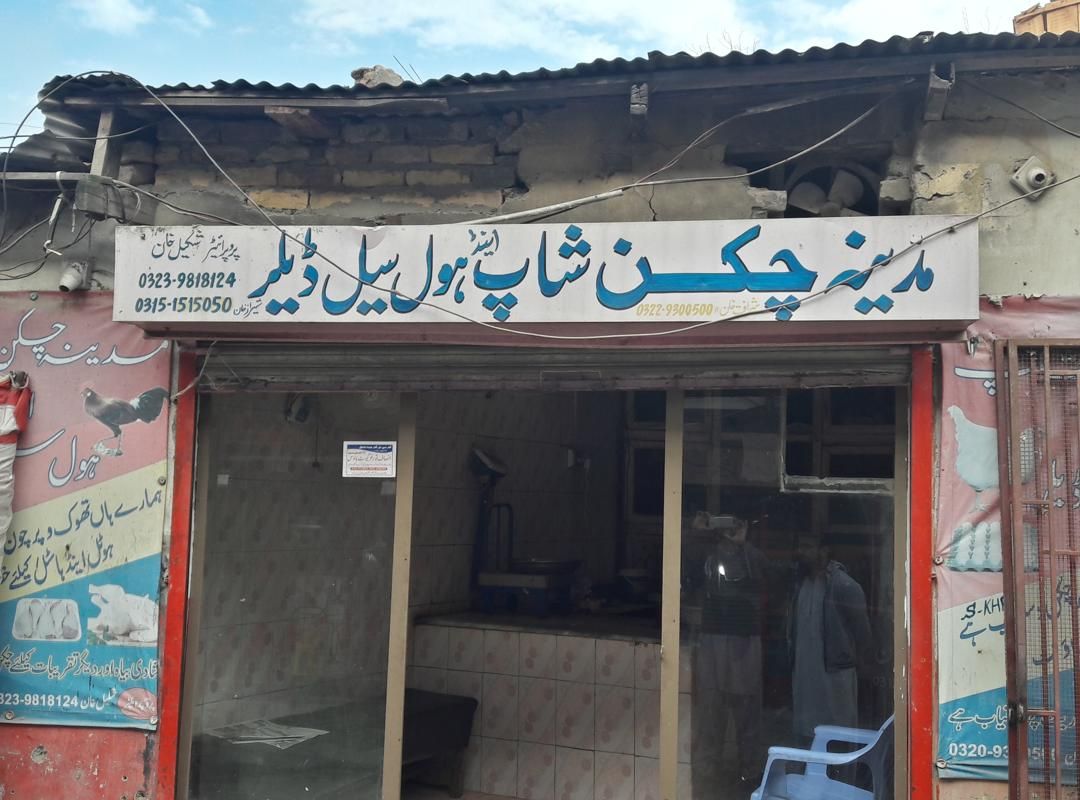

Supplement: Supplemental Information 2 [file peerj-cs-07-717-s002.zip › Testing Dataset/21.jpg]

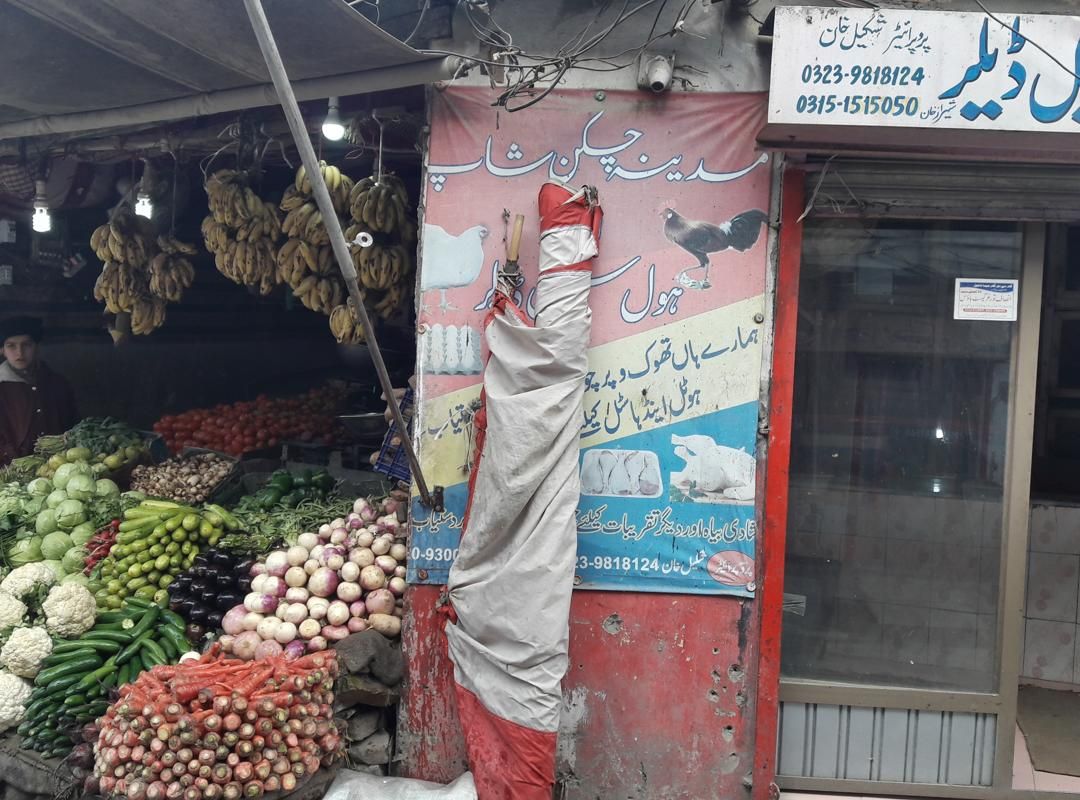

Supplement: Supplemental Information 2 [file peerj-cs-07-717-s002.zip › Testing Dataset/22.jpg]

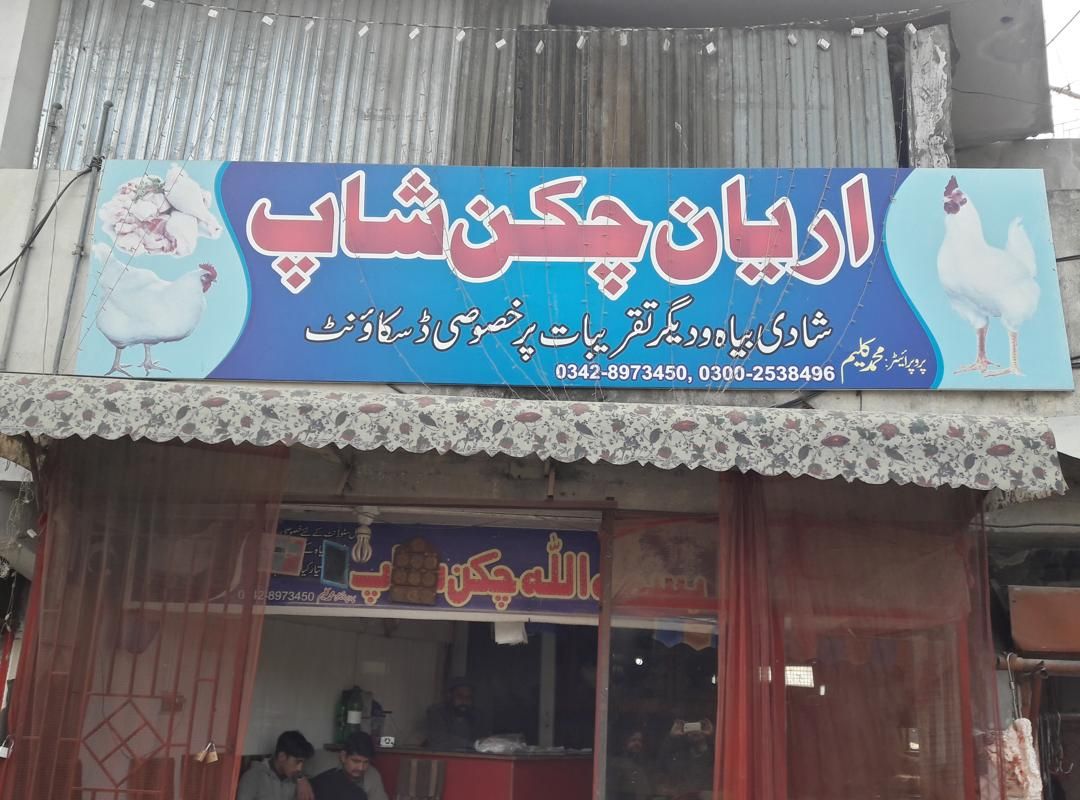

Supplement: Supplemental Information 2 [file peerj-cs-07-717-s002.zip › Testing Dataset/23.jpg]

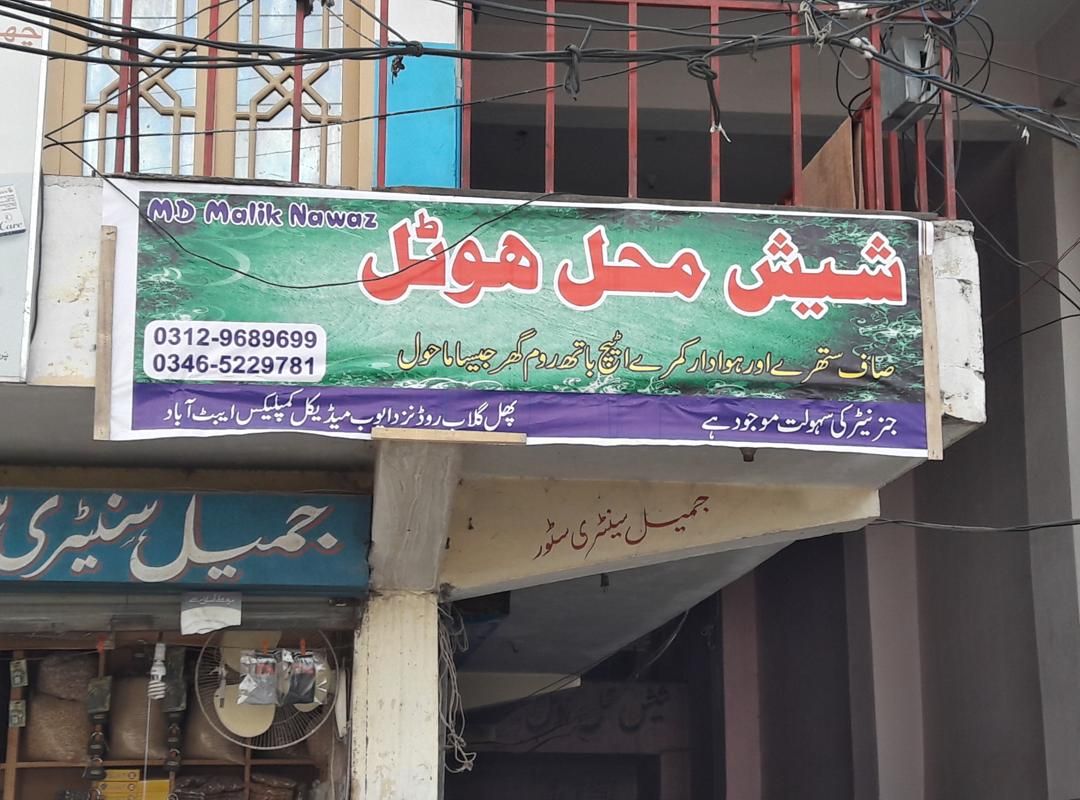

Supplement: Supplemental Information 2 [file peerj-cs-07-717-s002.zip › Testing Dataset/24.jpg]

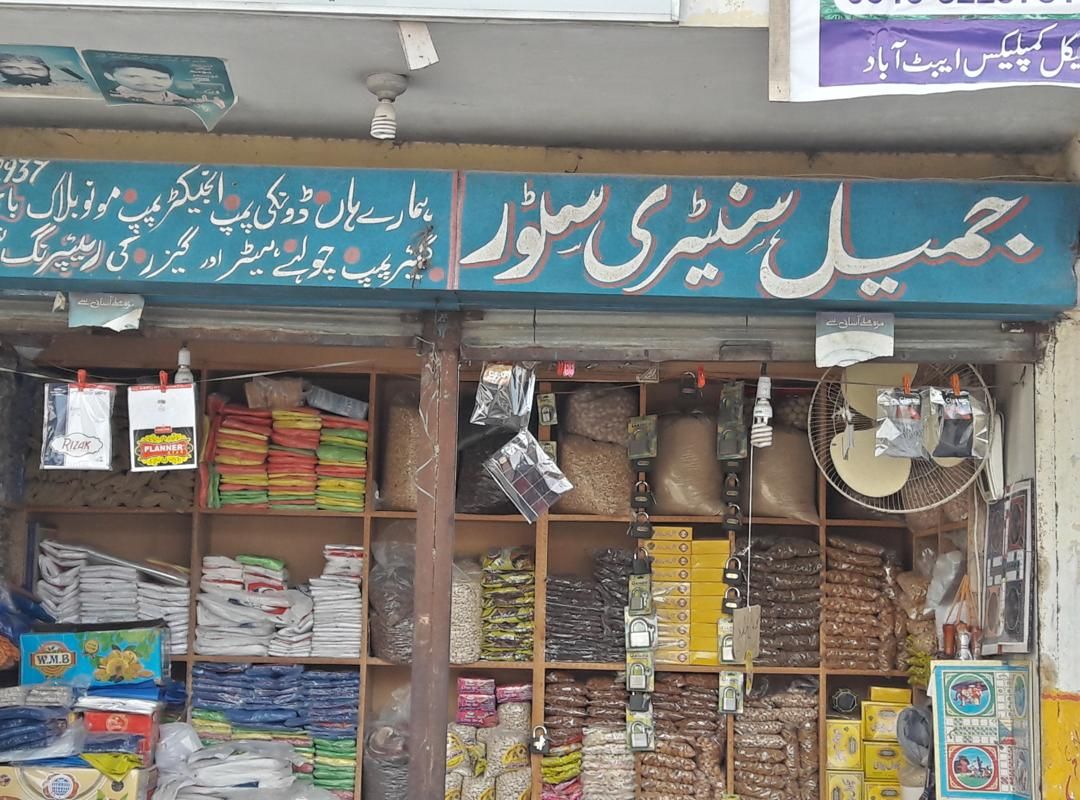

Supplement: Supplemental Information 2 [file peerj-cs-07-717-s002.zip › Testing Dataset/25.jpg]

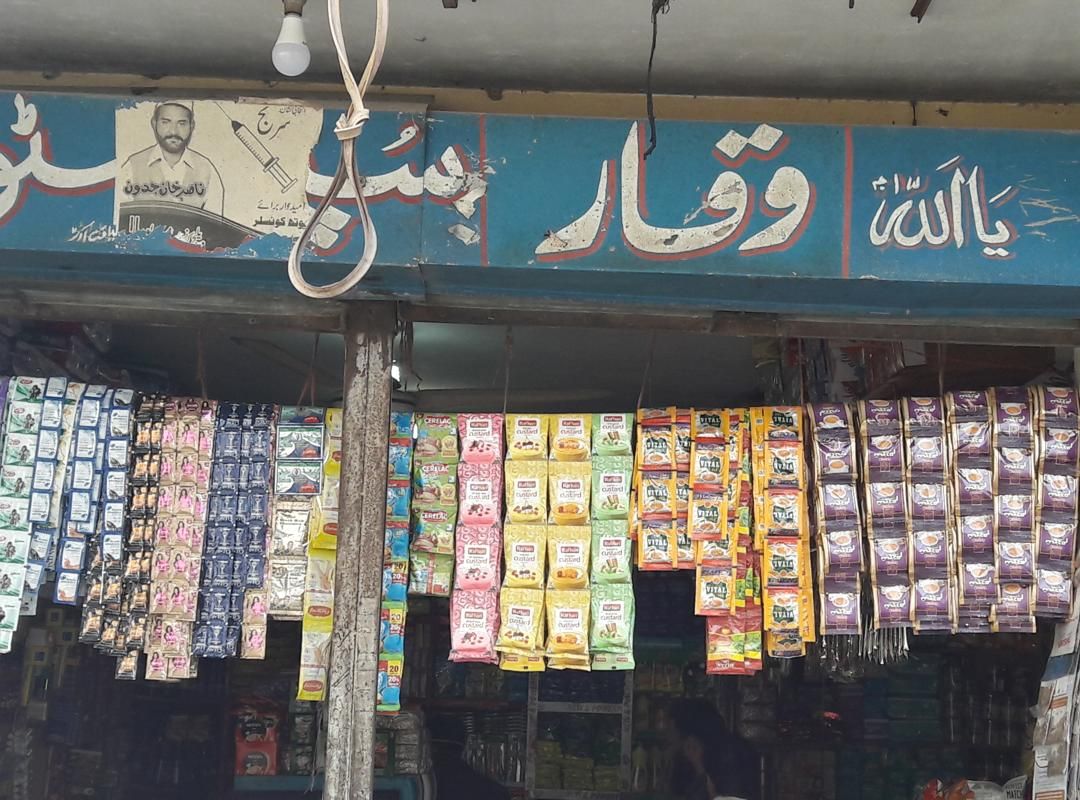

Supplement: Supplemental Information 2 [file peerj-cs-07-717-s002.zip › Testing Dataset/26.jpg]

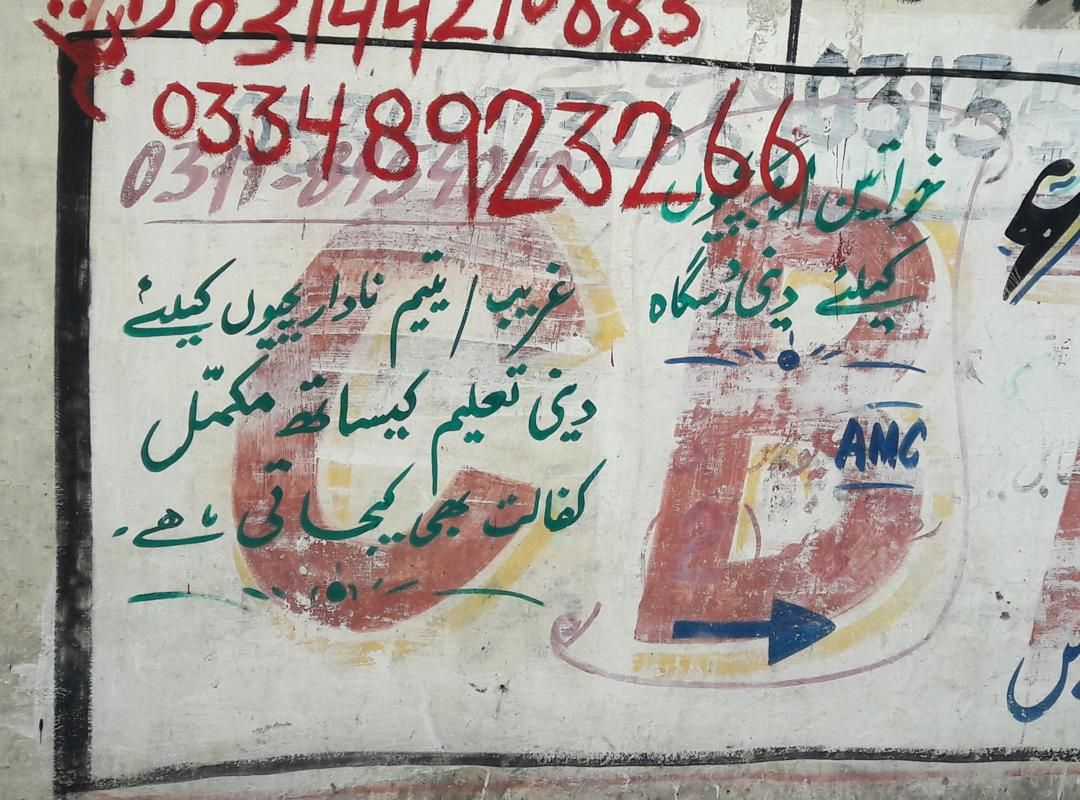

Supplement: Supplemental Information 2 [file peerj-cs-07-717-s002.zip › Testing Dataset/27.jpg]

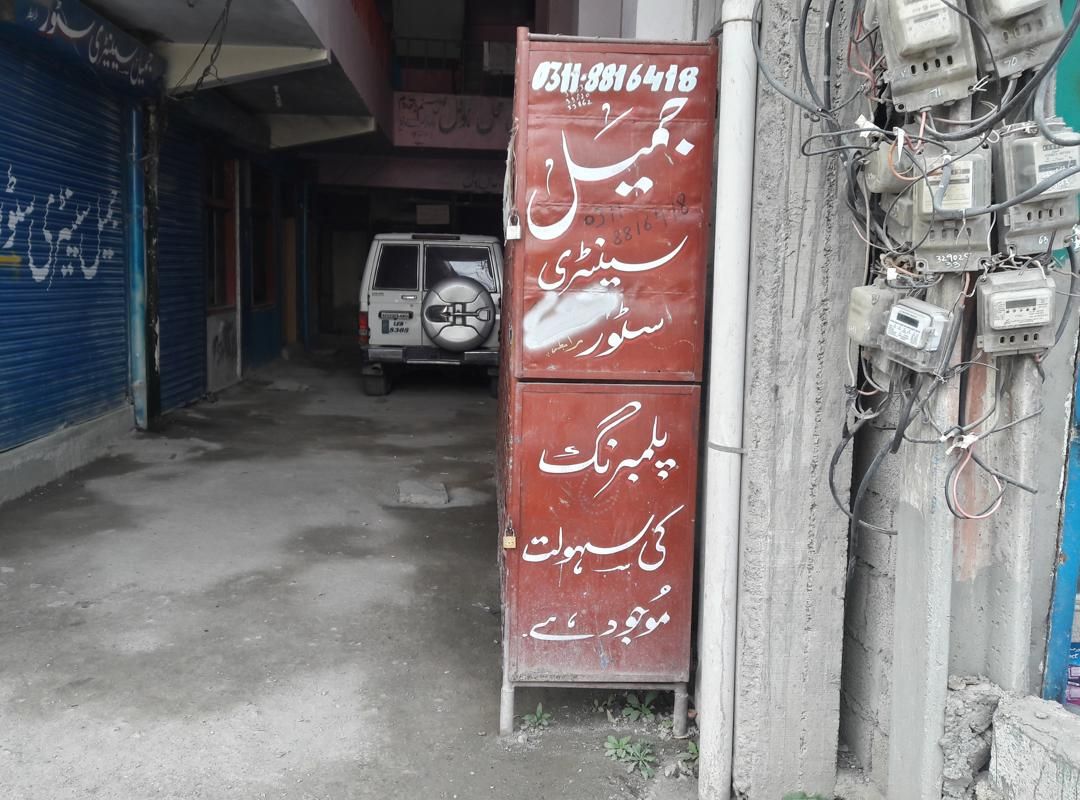

Supplement: Supplemental Information 2 [file peerj-cs-07-717-s002.zip › Testing Dataset/28.jpg]

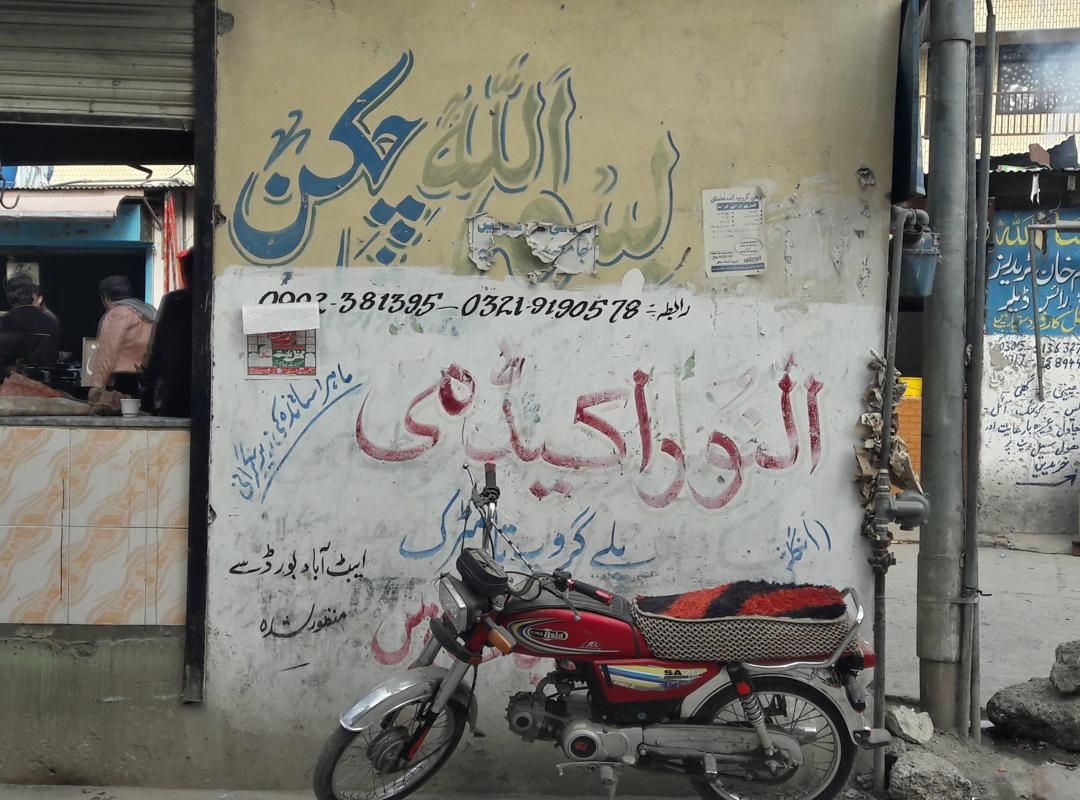

Supplement: Supplemental Information 2 [file peerj-cs-07-717-s002.zip › Testing Dataset/29.jpg]

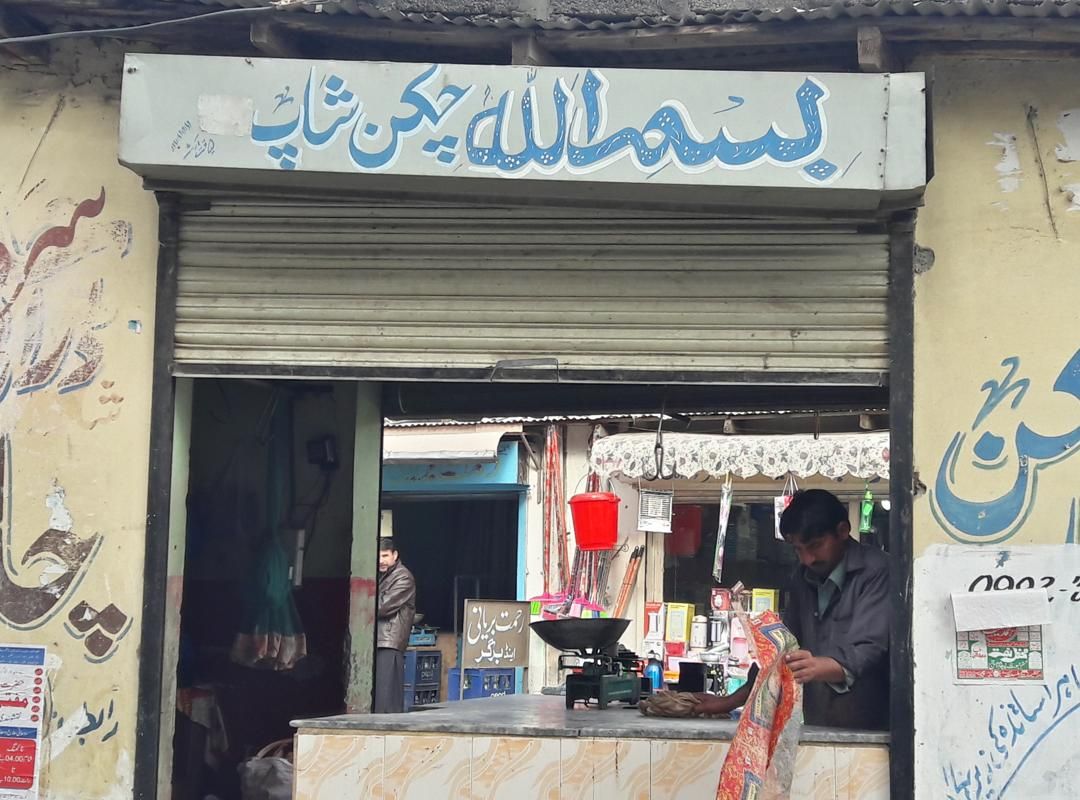

Supplement: Supplemental Information 2 [file peerj-cs-07-717-s002.zip › Testing Dataset/30.jpg]

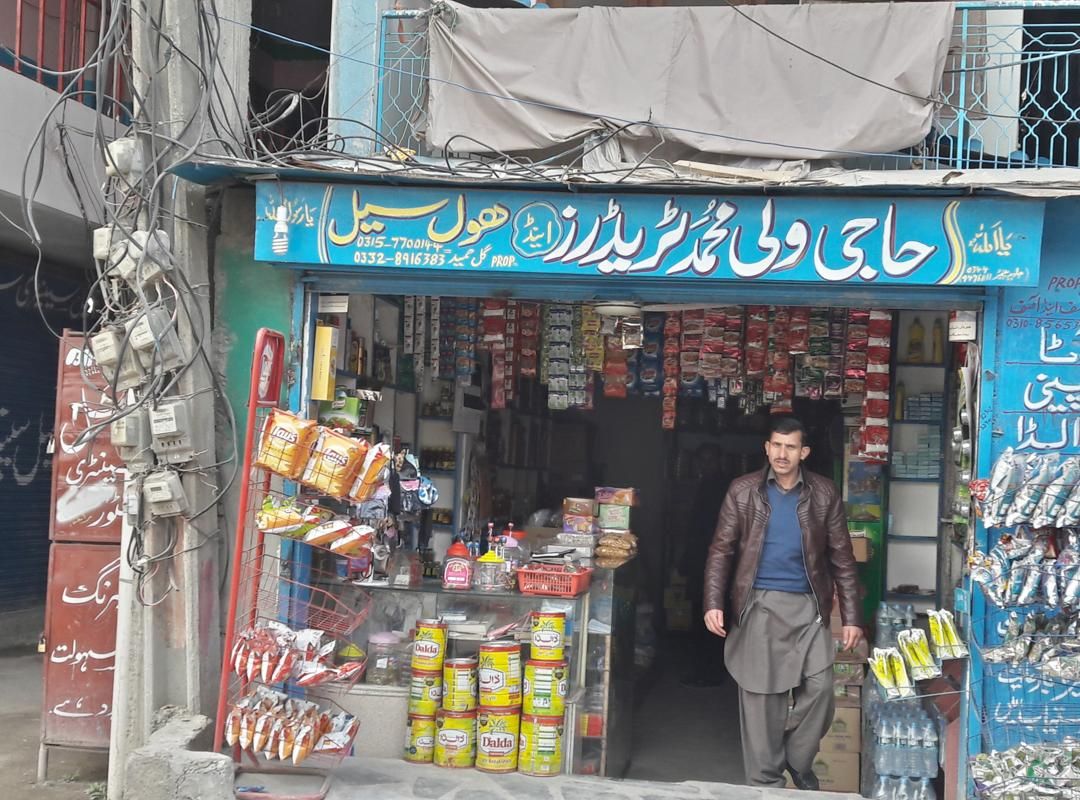

Supplement: Supplemental Information 2 [file peerj-cs-07-717-s002.zip › Testing Dataset/31.jpg]

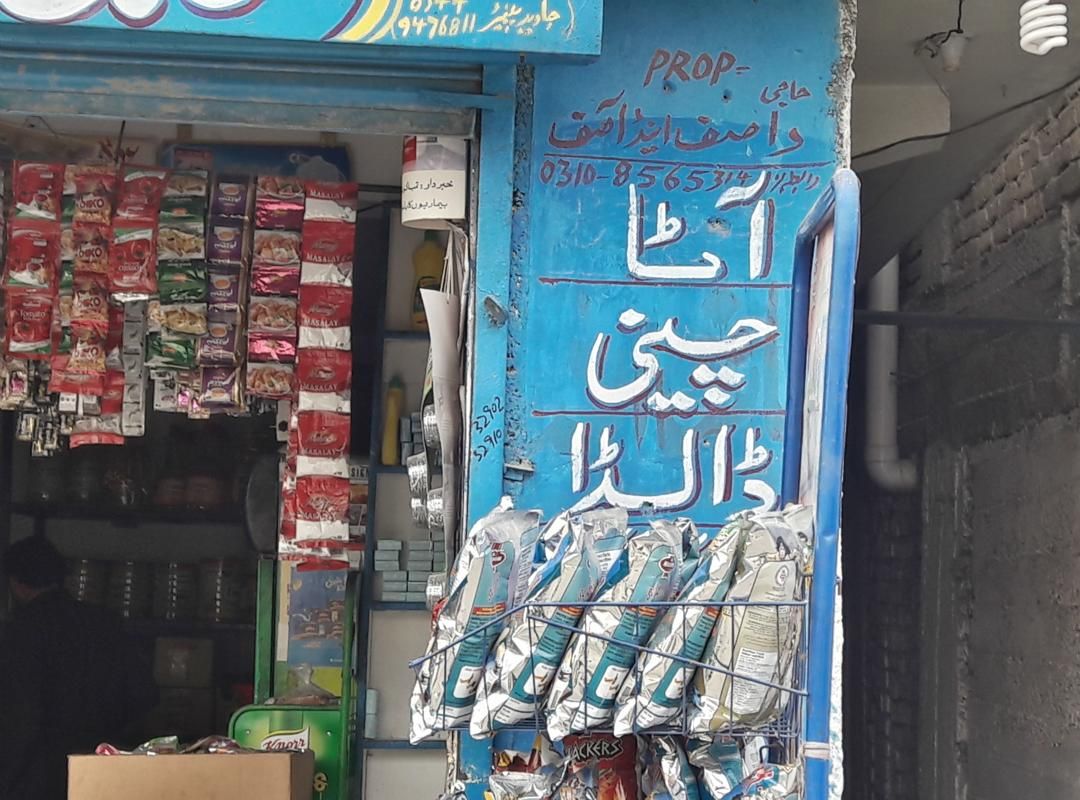

Supplement: Supplemental Information 2 [file peerj-cs-07-717-s002.zip › Testing Dataset/32.jpg]

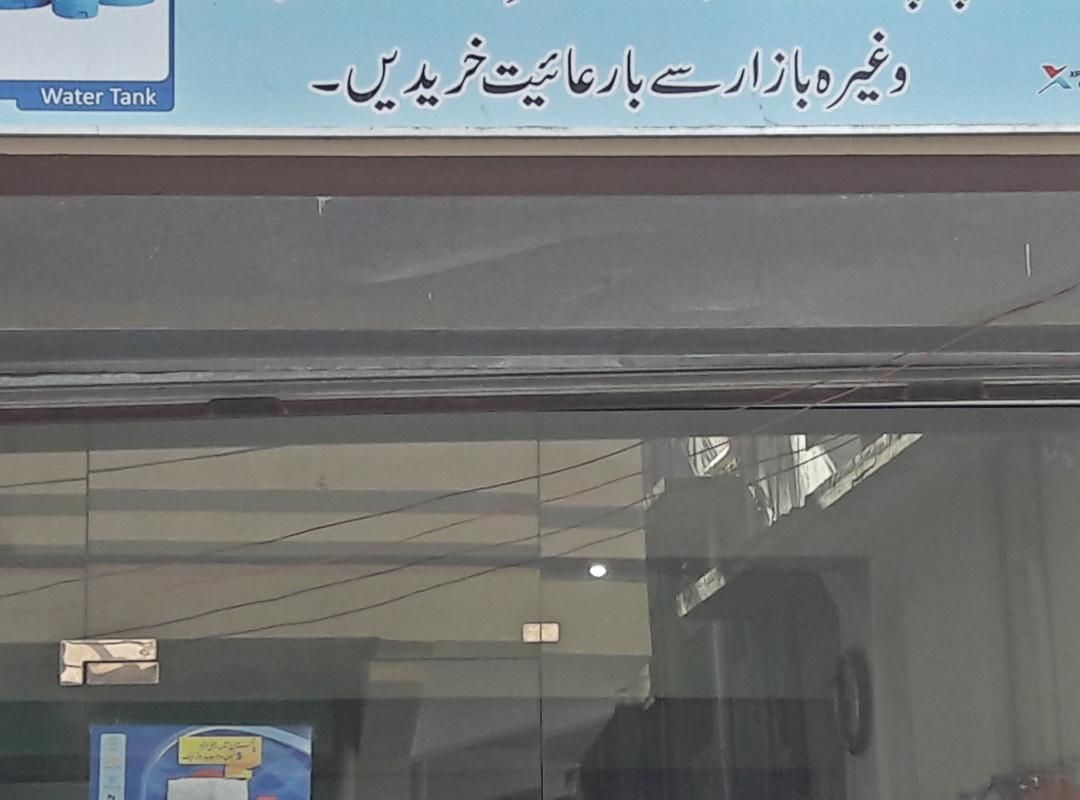

Supplement: Supplemental Information 2 [file peerj-cs-07-717-s002.zip › Testing Dataset/33.jpg]

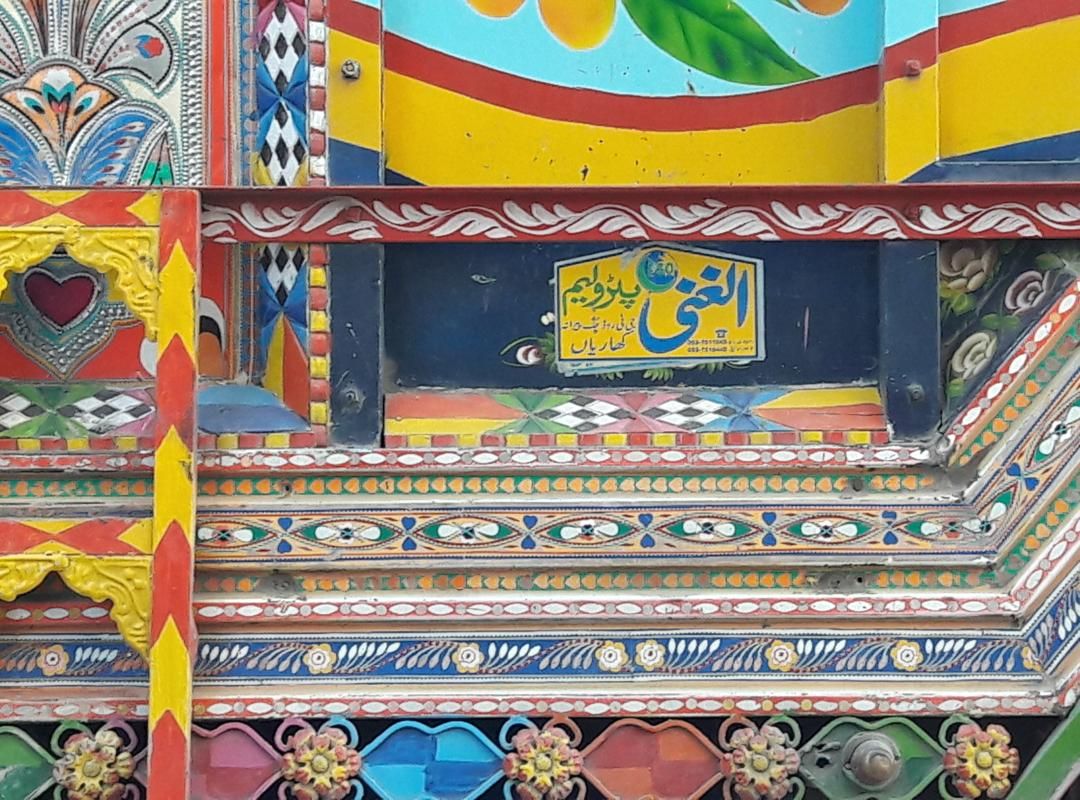

Supplement: Supplemental Information 2 [file peerj-cs-07-717-s002.zip › Testing Dataset/34.jpg]

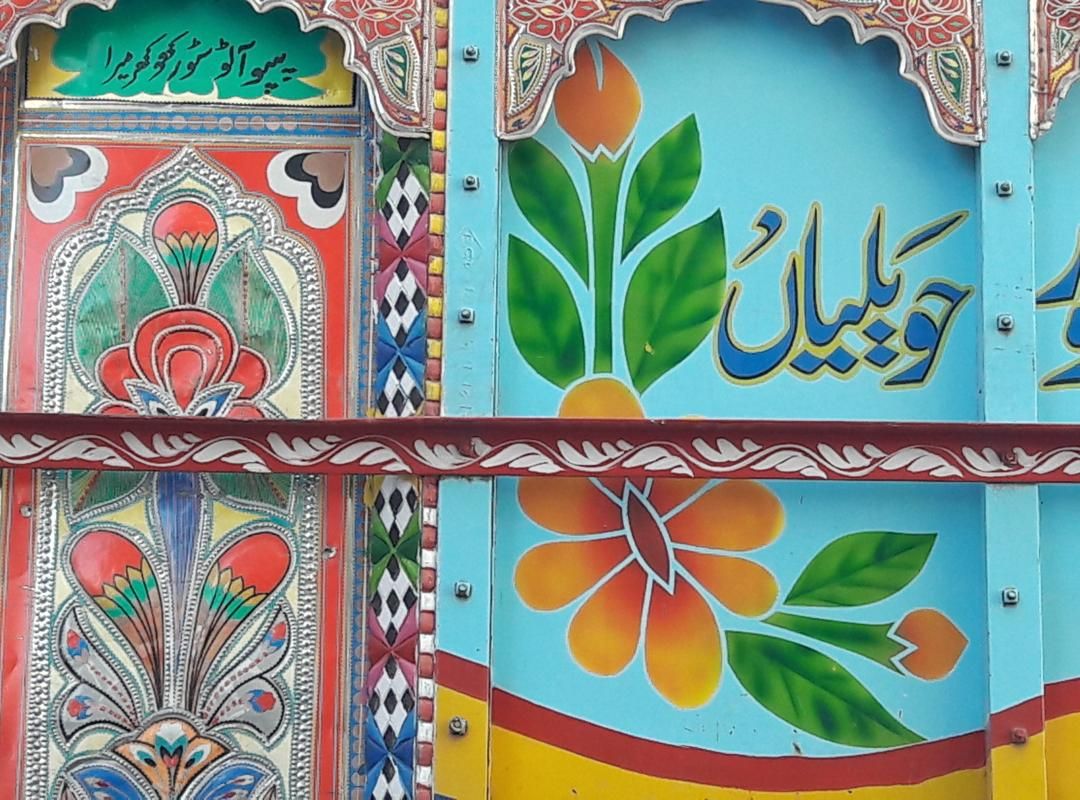

Supplement: Supplemental Information 2 [file peerj-cs-07-717-s002.zip › Testing Dataset/35.jpg]

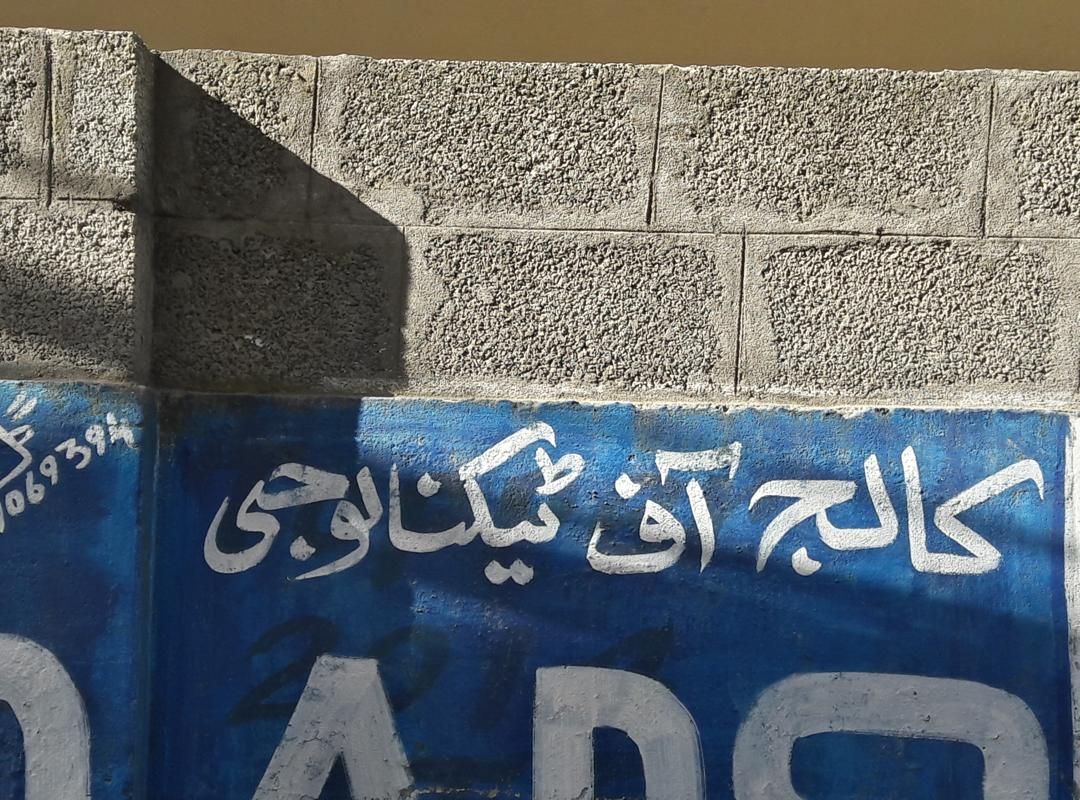

Supplement: Supplemental Information 2 [file peerj-cs-07-717-s002.zip › Testing Dataset/36.jpg]

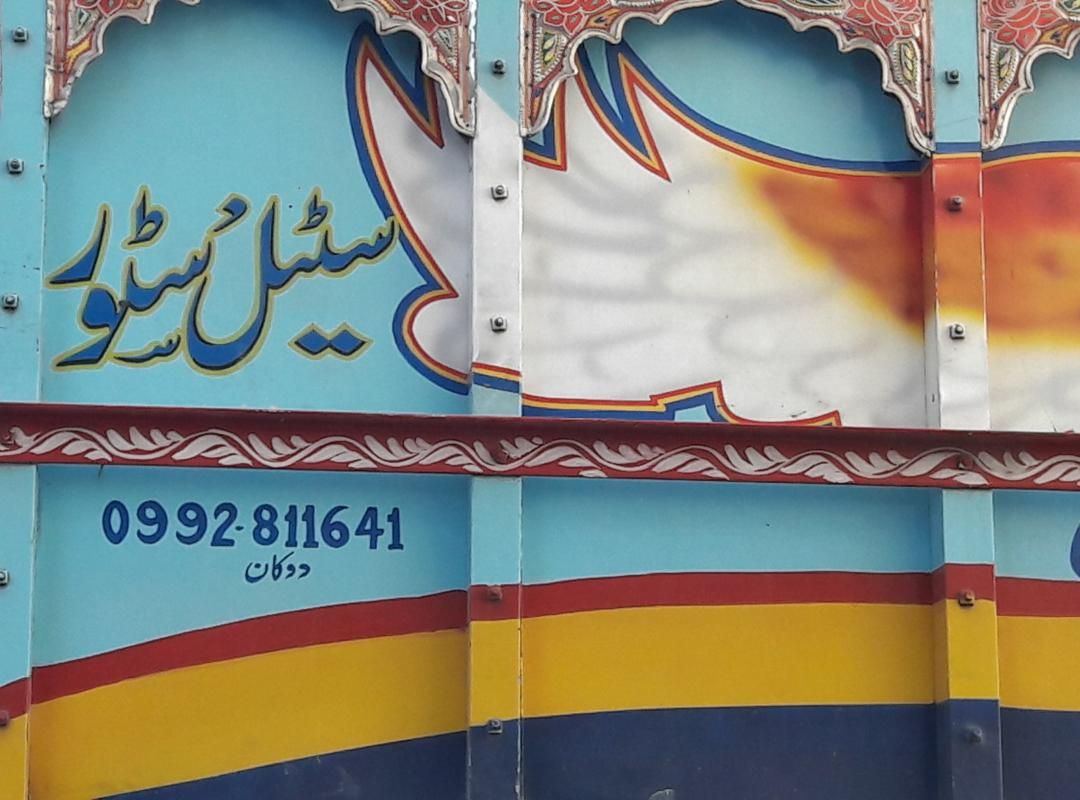

Supplement: Supplemental Information 2 [file peerj-cs-07-717-s002.zip › Testing Dataset/37.jpg]

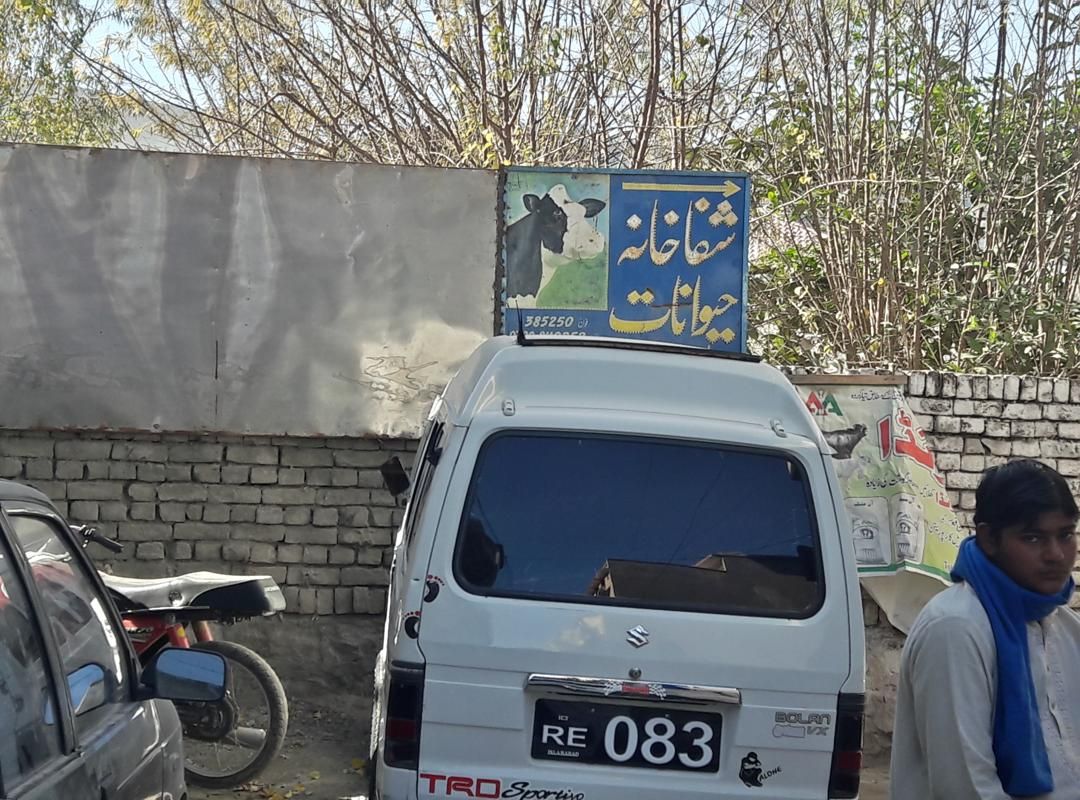

Supplement: Supplemental Information 2 [file peerj-cs-07-717-s002.zip › Testing Dataset/38.jpg]

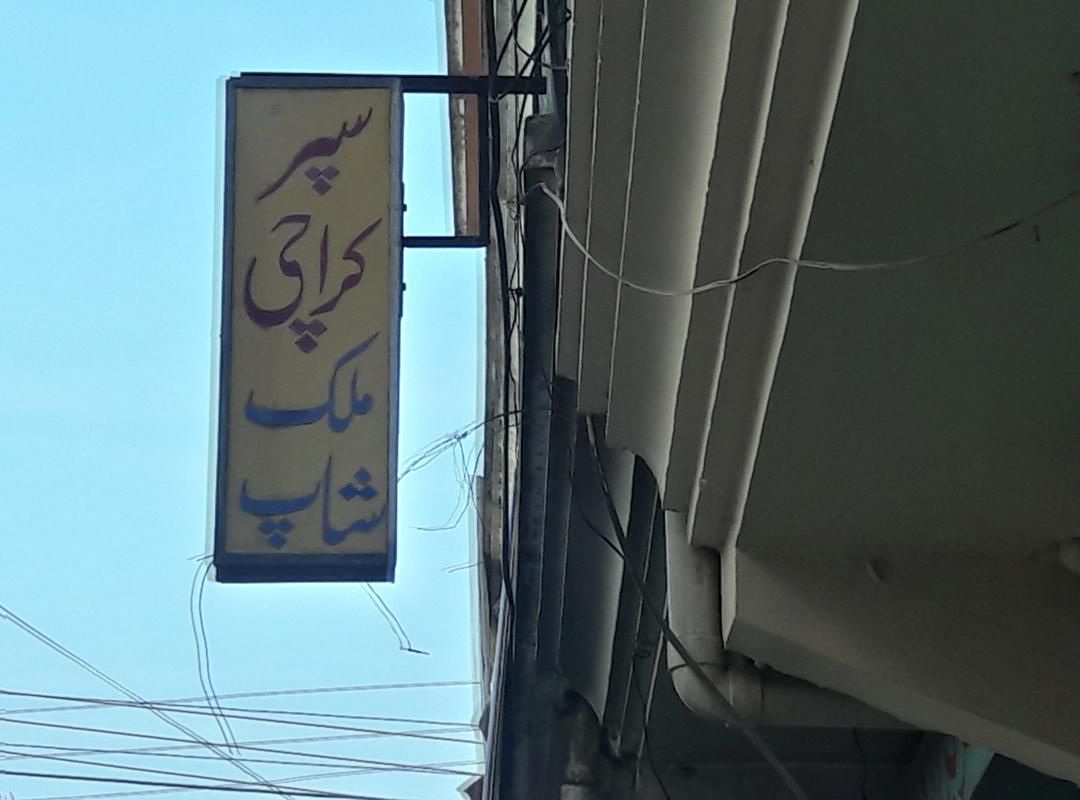

Supplement: Supplemental Information 2 [file peerj-cs-07-717-s002.zip › Testing Dataset/39.jpg]

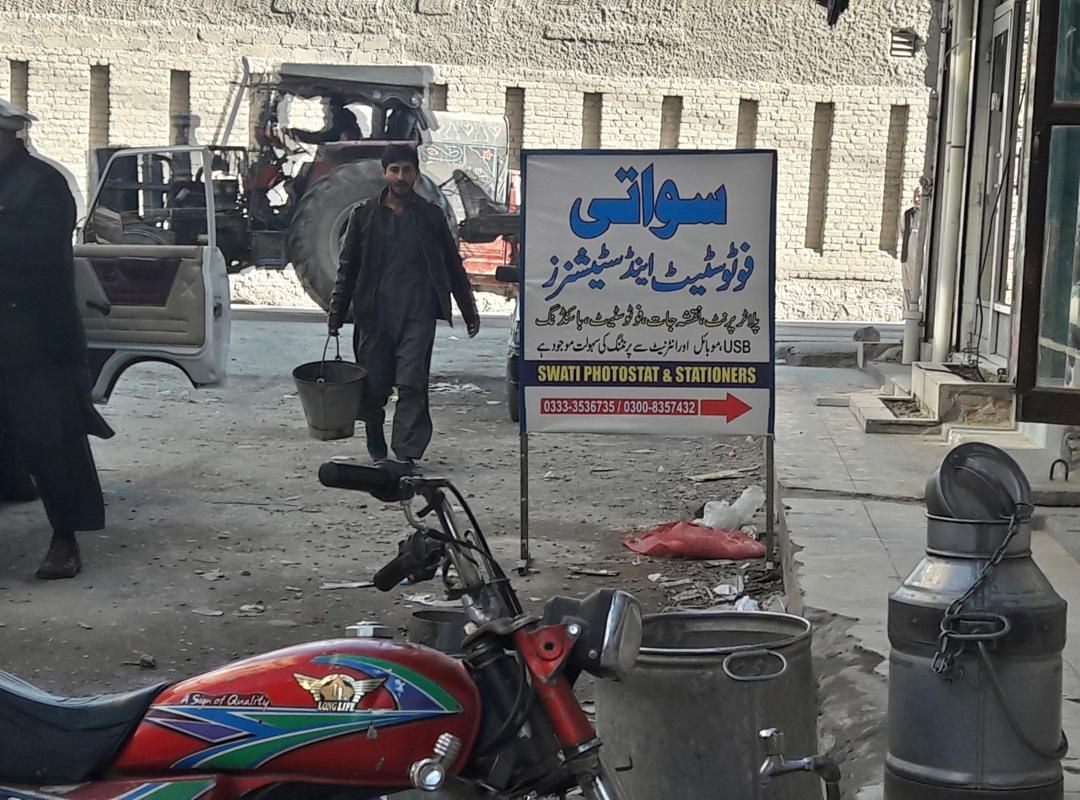

Supplement: Supplemental Information 2 [file peerj-cs-07-717-s002.zip › Testing Dataset/40.jpg]

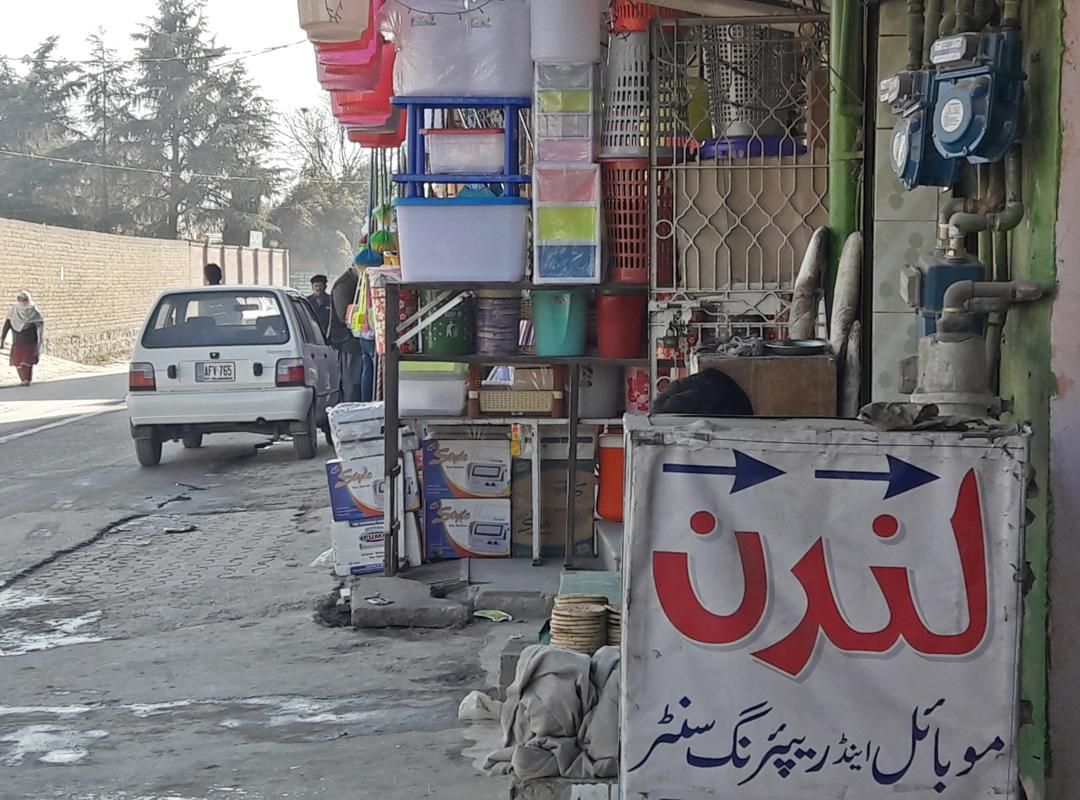

Supplement: Supplemental Information 2 [file peerj-cs-07-717-s002.zip › Testing Dataset/41.jpg]

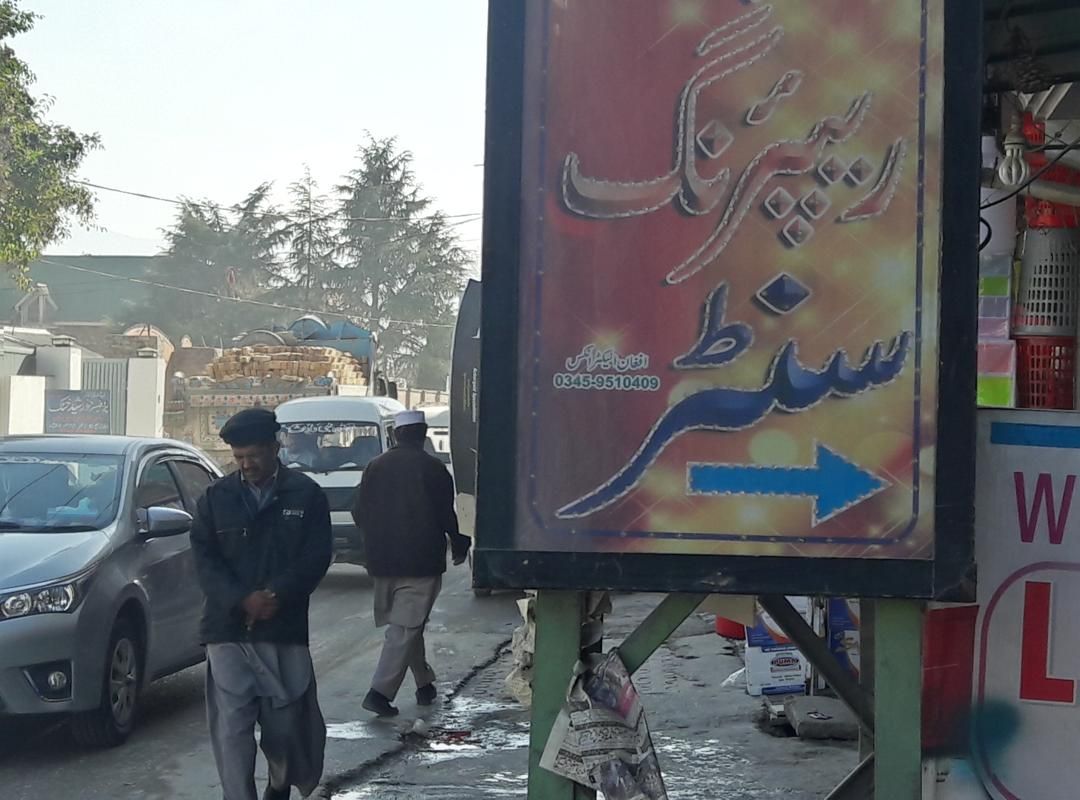

Supplement: Supplemental Information 2 [file peerj-cs-07-717-s002.zip › Testing Dataset/42.jpg]

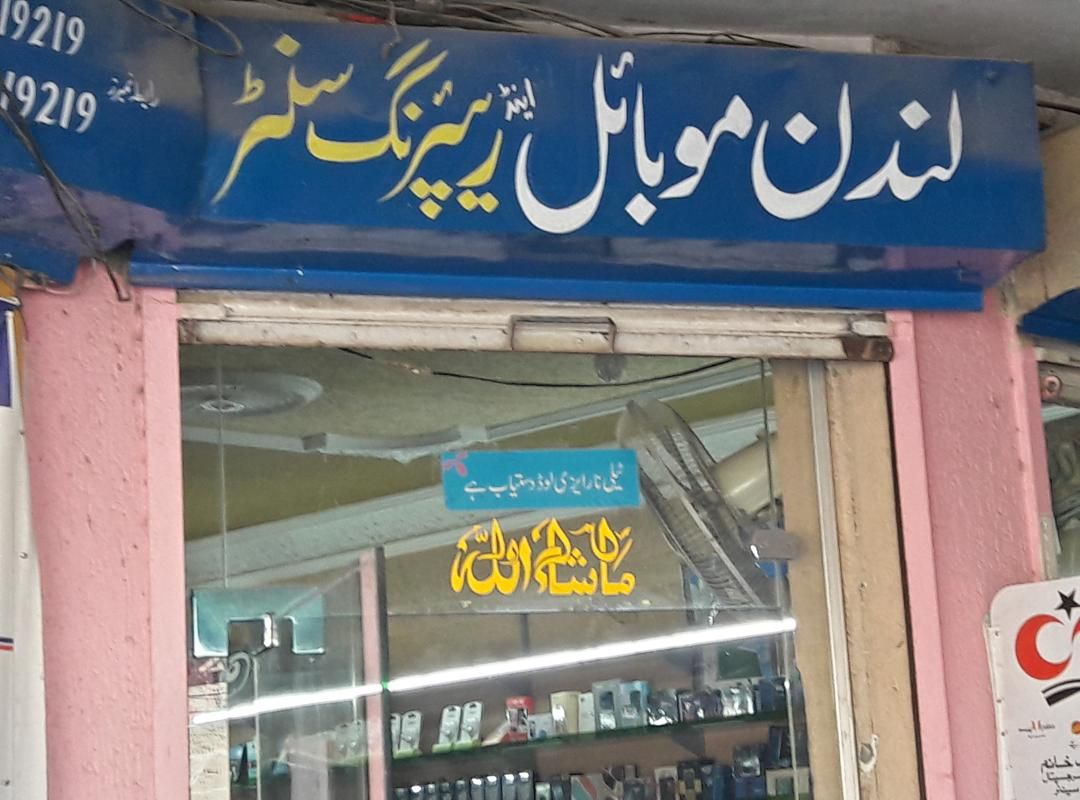

Supplement: Supplemental Information 2 [file peerj-cs-07-717-s002.zip › Testing Dataset/43.jpg]

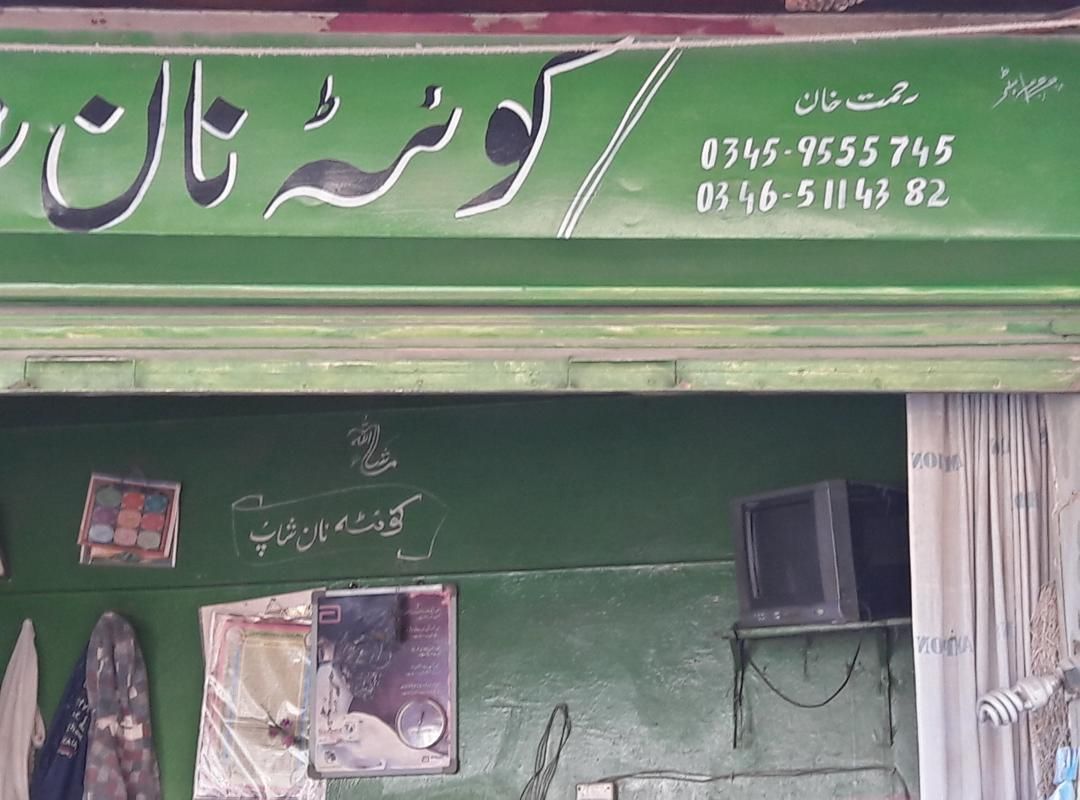

Supplement: Supplemental Information 2 [file peerj-cs-07-717-s002.zip › Testing Dataset/44.jpg]

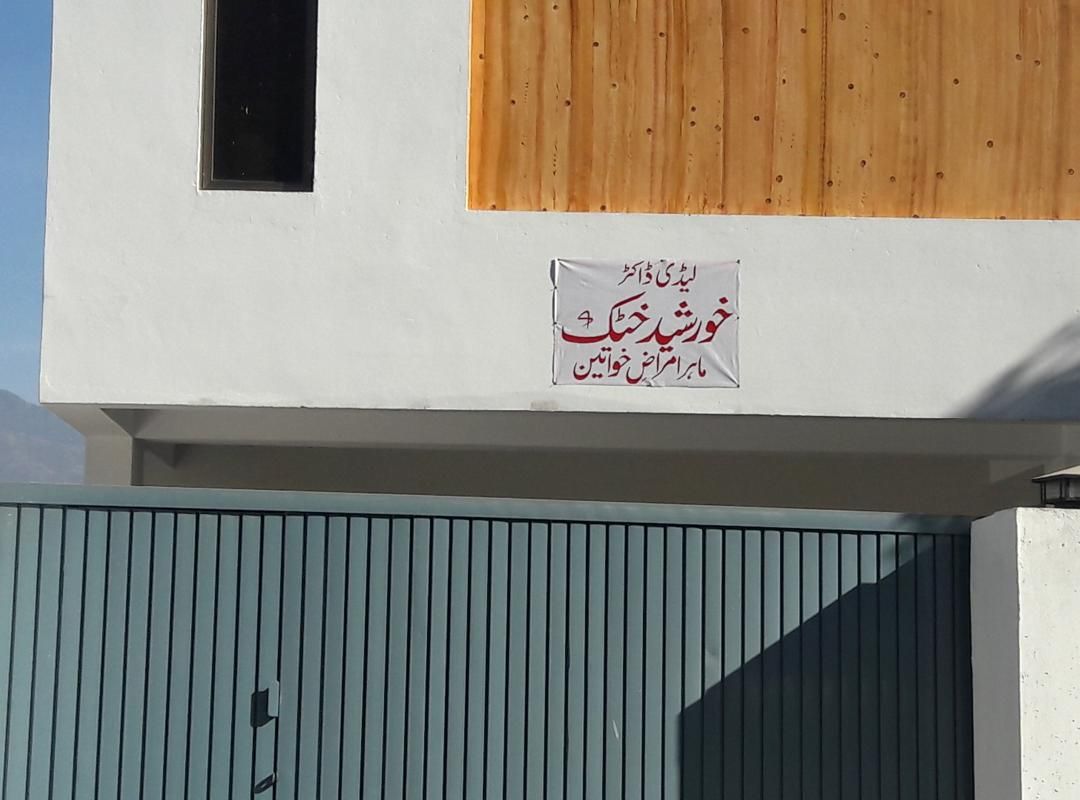

Supplement: Supplemental Information 2 [file peerj-cs-07-717-s002.zip › Testing Dataset/45.jpg]

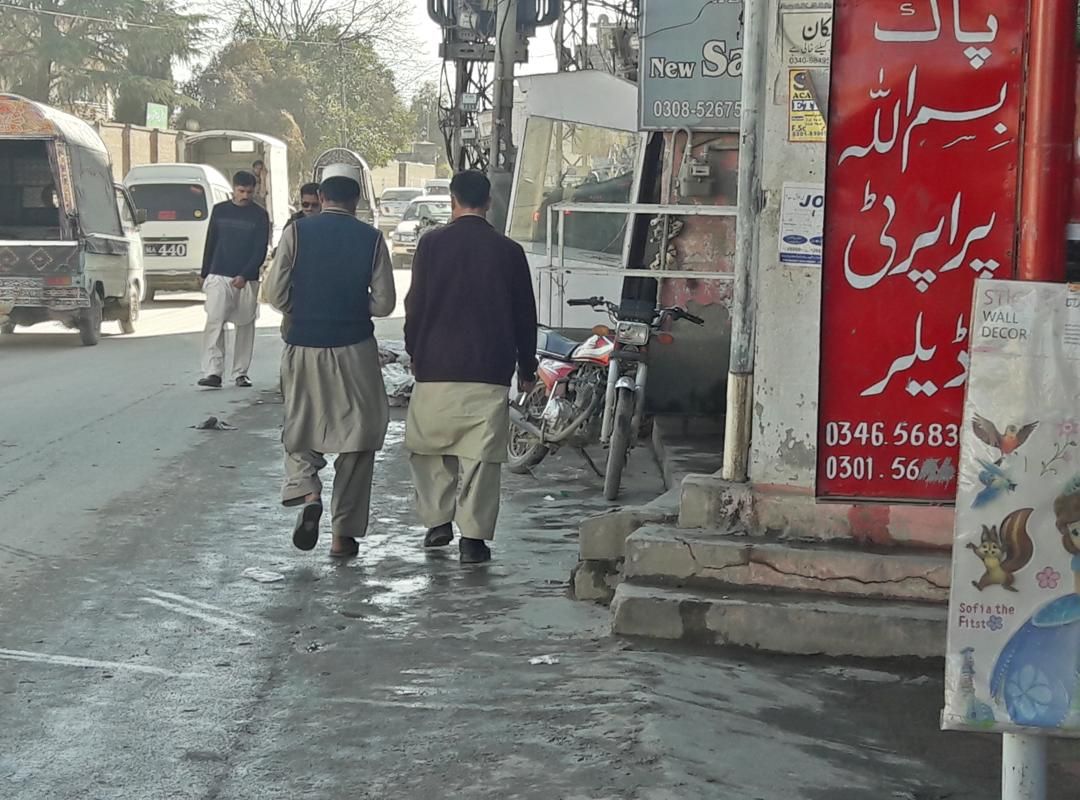

Supplement: Supplemental Information 2 [file peerj-cs-07-717-s002.zip › Testing Dataset/46.jpg]

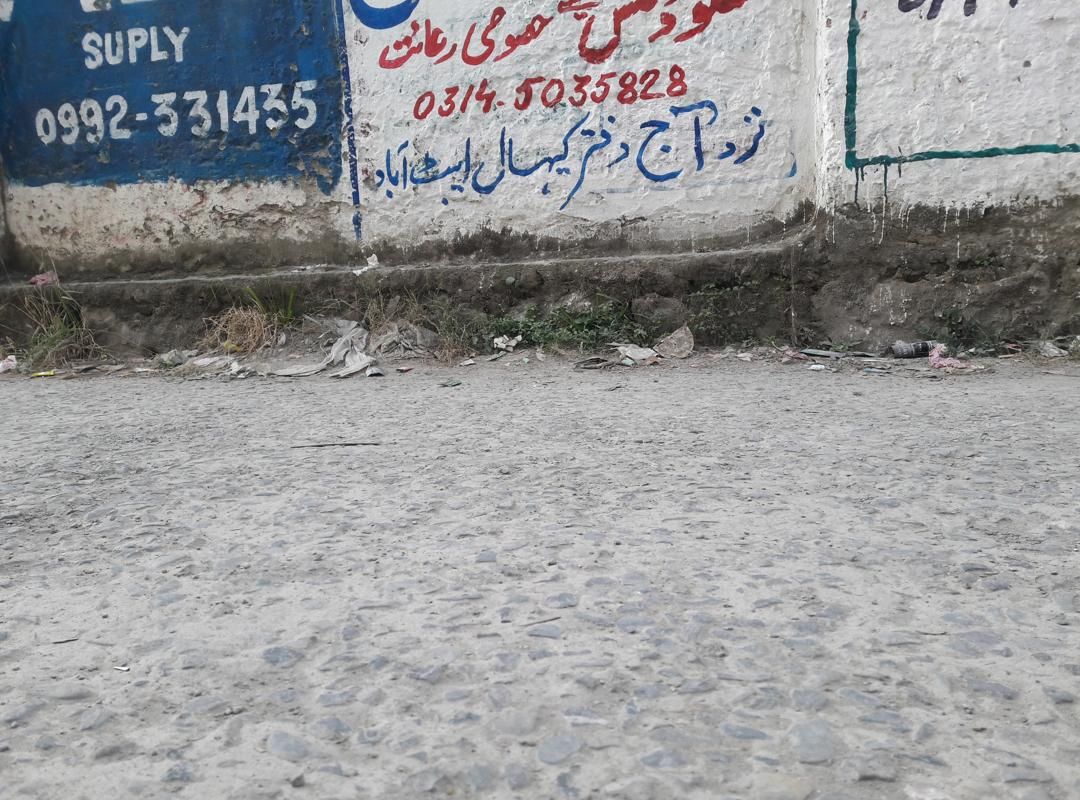

Supplement: Supplemental Information 2 [file peerj-cs-07-717-s002.zip › Testing Dataset/47.jpg]

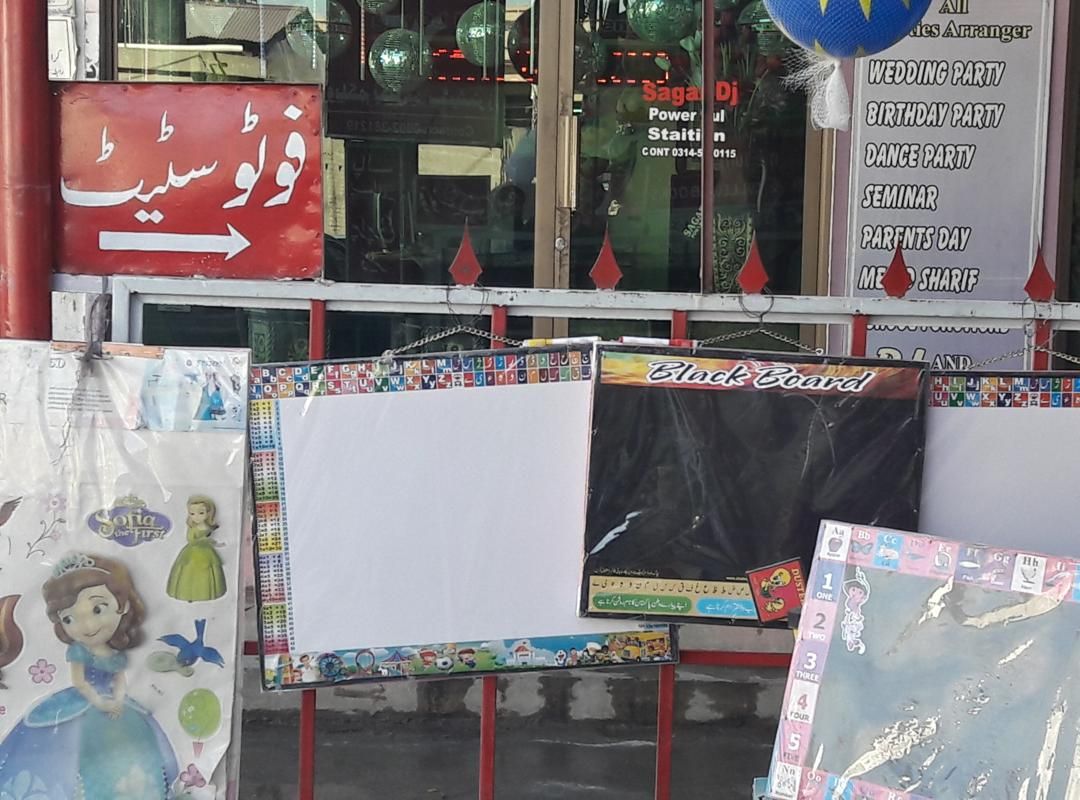

Supplement: Supplemental Information 2 [file peerj-cs-07-717-s002.zip › Testing Dataset/48.jpg]

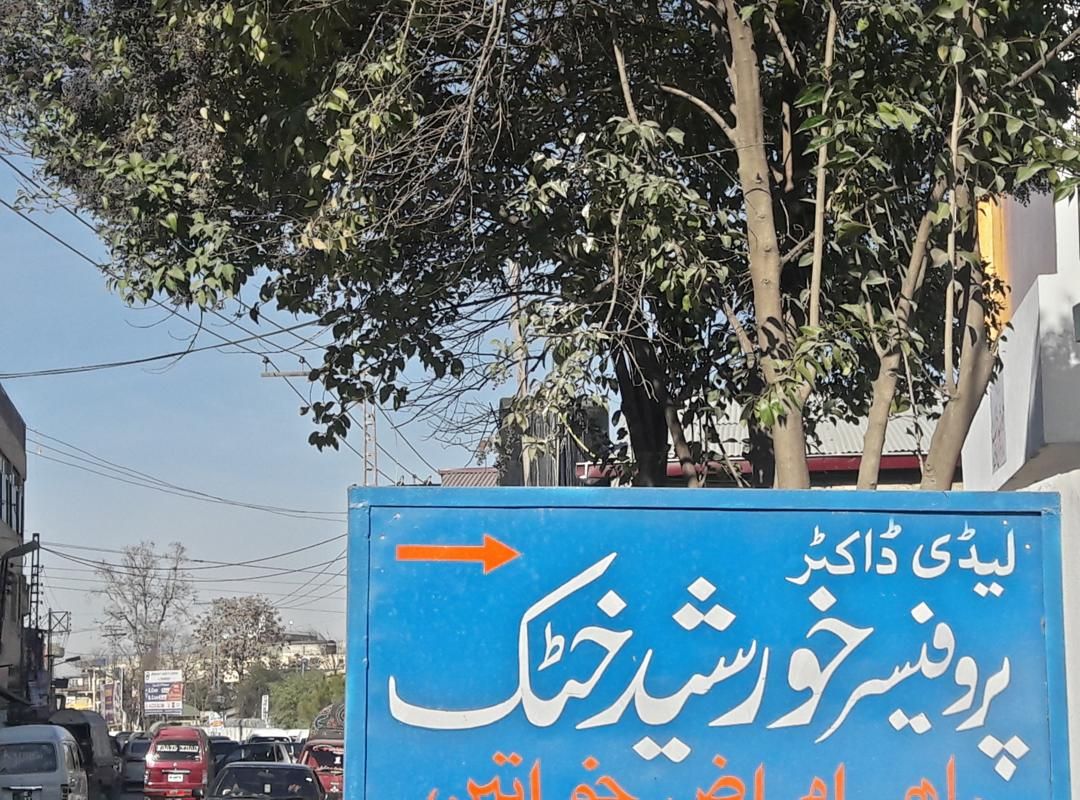

Supplement: Supplemental Information 2 [file peerj-cs-07-717-s002.zip › Testing Dataset/49.jpg]

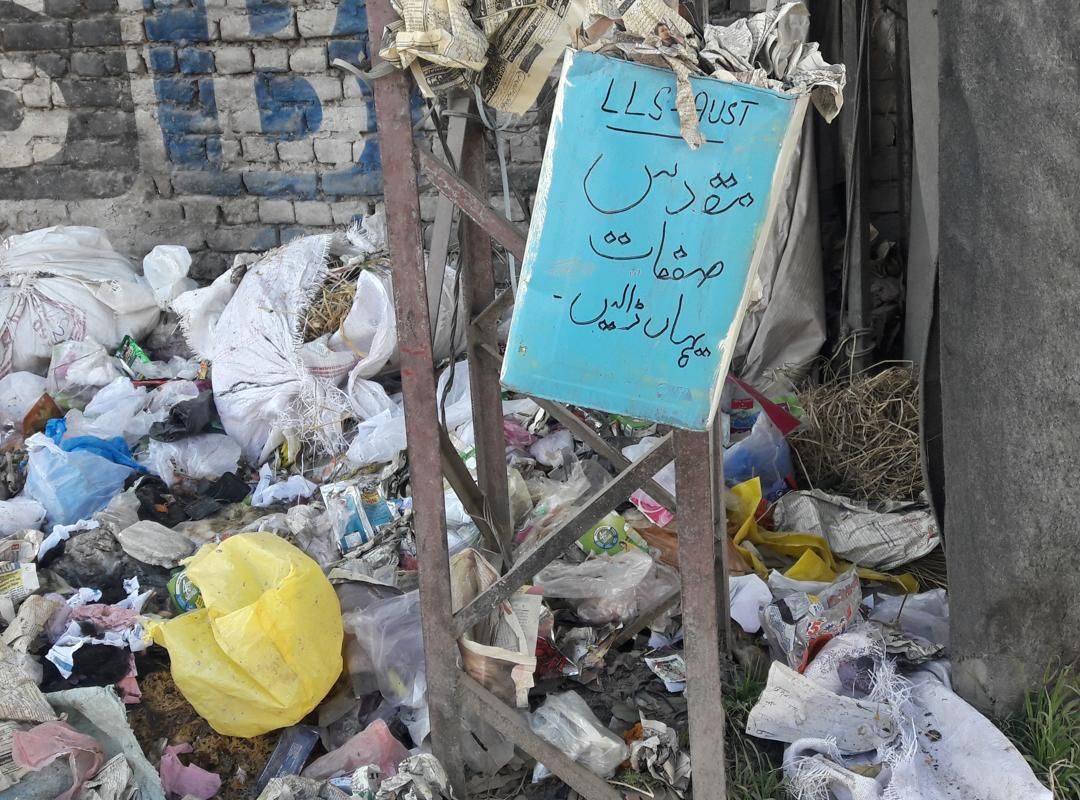

Supplement: Supplemental Information 2 [file peerj-cs-07-717-s002.zip › Testing Dataset/50.jpg]

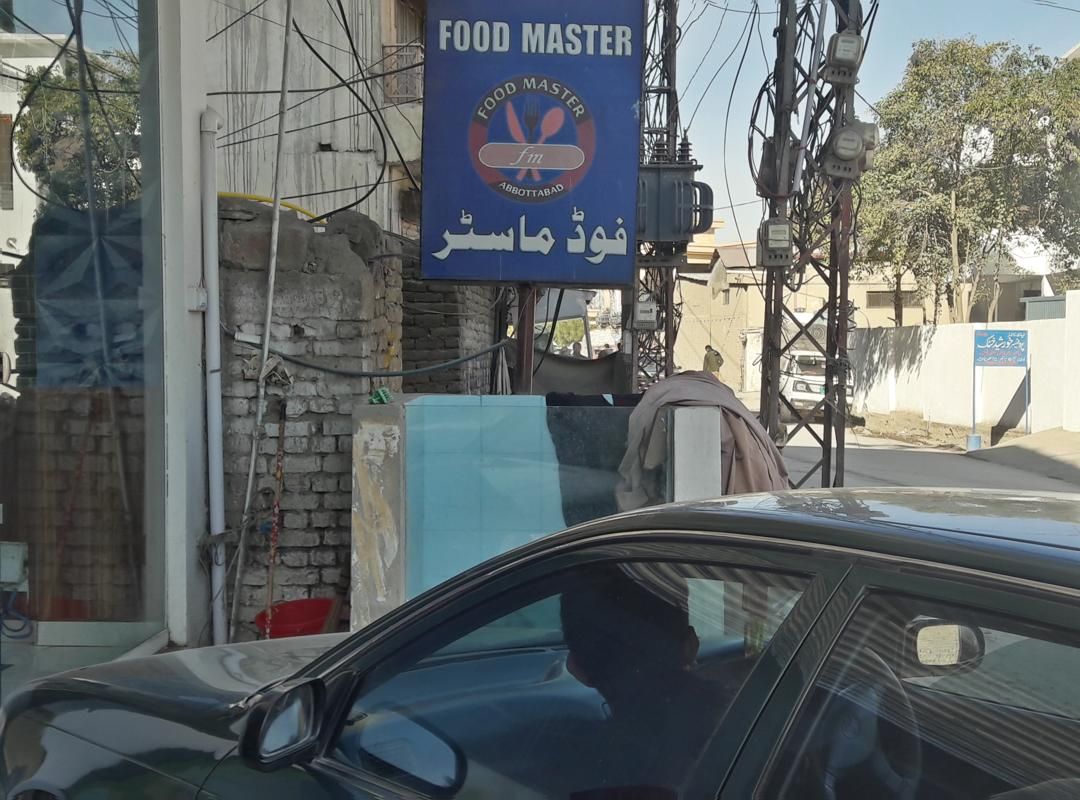

Supplement: Supplemental Information 2 [file peerj-cs-07-717-s002.zip › Testing Dataset/51.jpg]

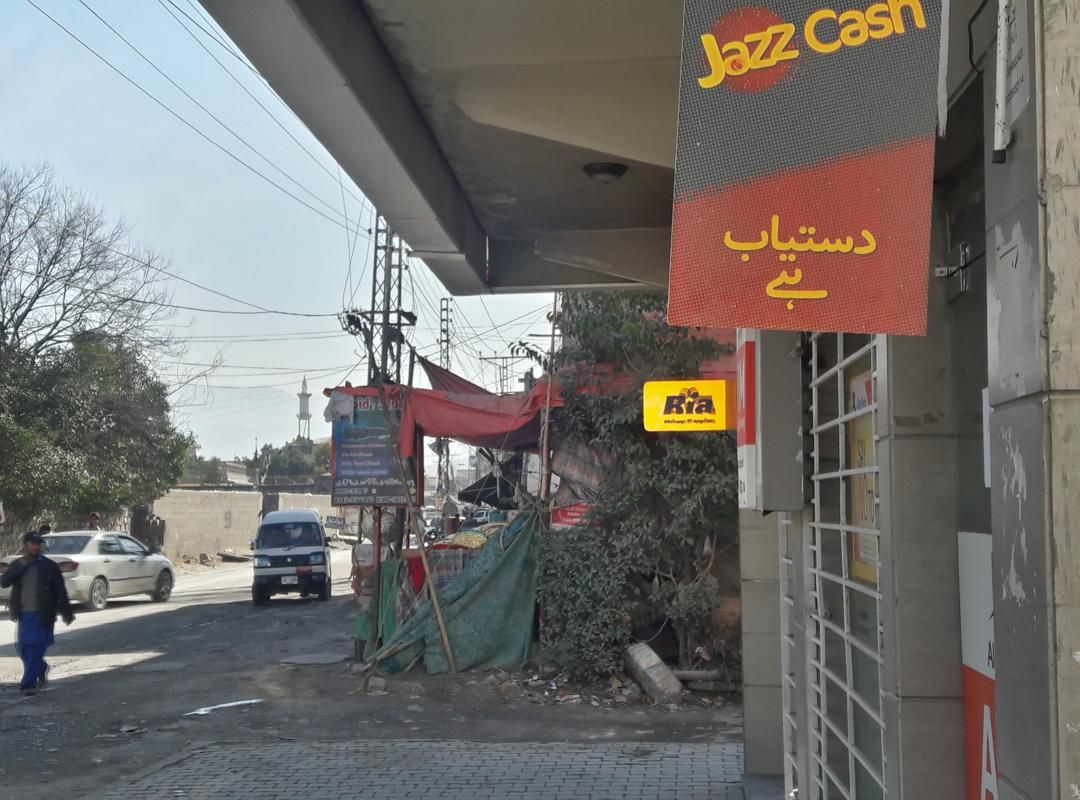

Supplement: Supplemental Information 2 [file peerj-cs-07-717-s002.zip › Testing Dataset/52.jpg]

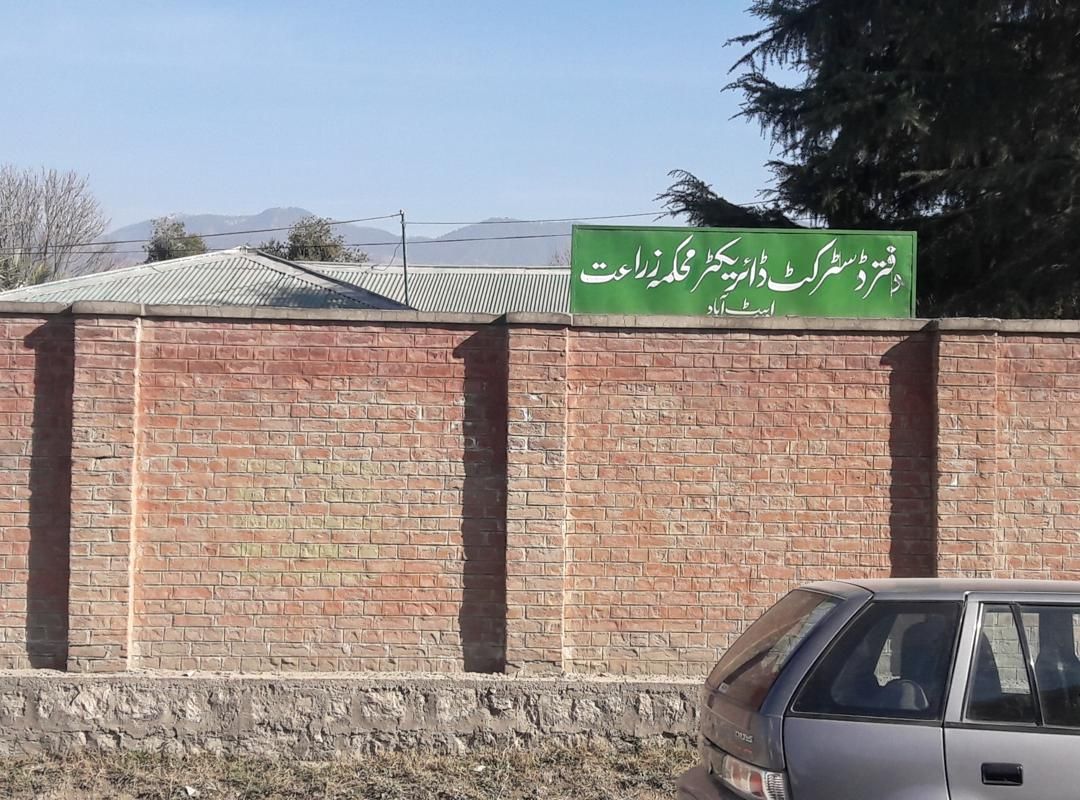

Supplement: Supplemental Information 2 [file peerj-cs-07-717-s002.zip › Testing Dataset/53.jpg]

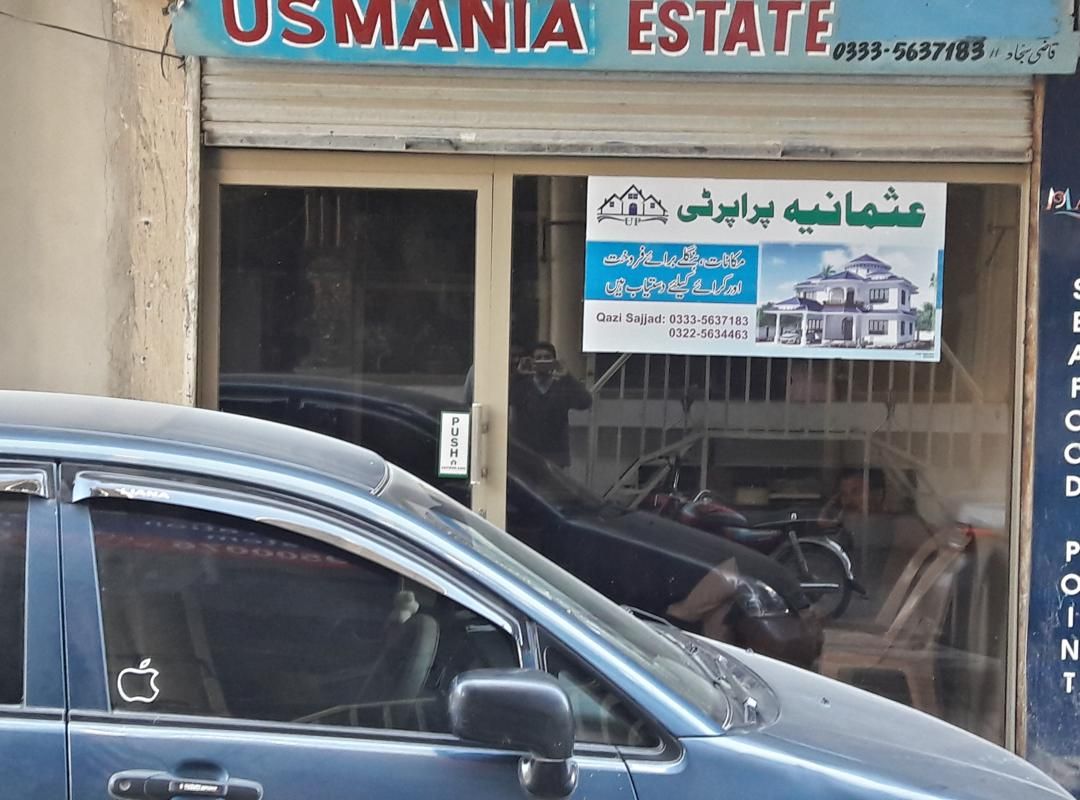

Supplement: Supplemental Information 2 [file peerj-cs-07-717-s002.zip › Testing Dataset/54.jpg]

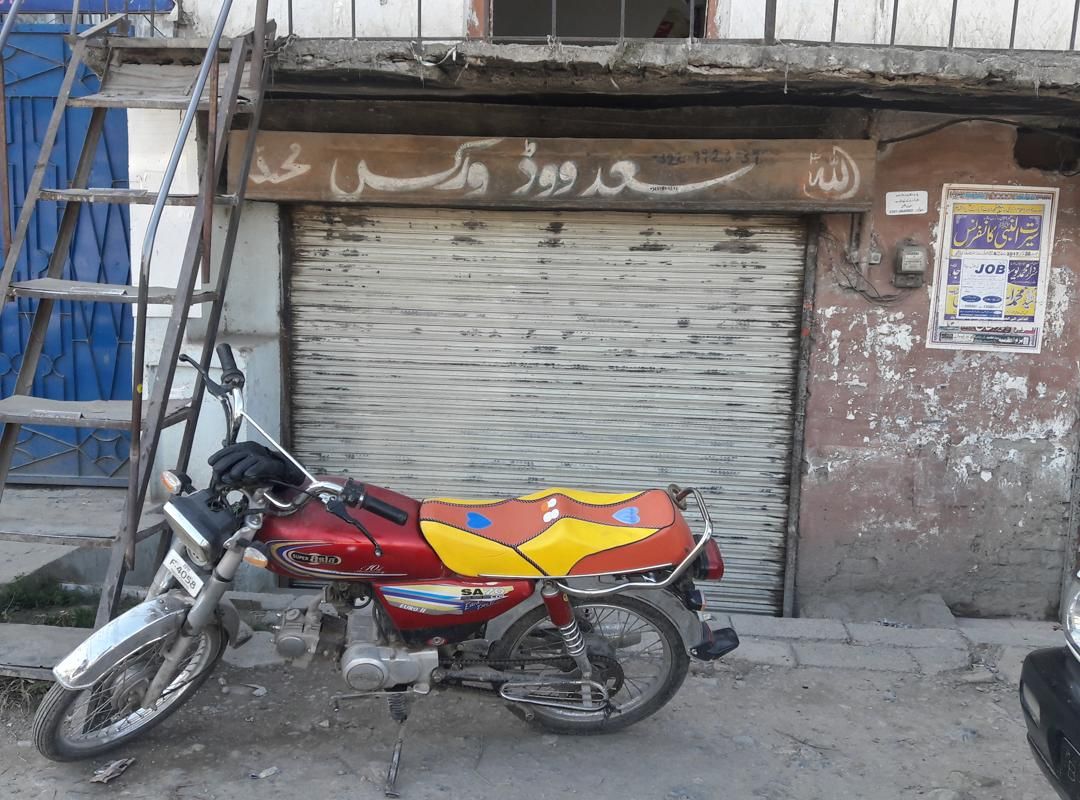

Supplement: Supplemental Information 2 [file peerj-cs-07-717-s002.zip › Testing Dataset/55.jpg]

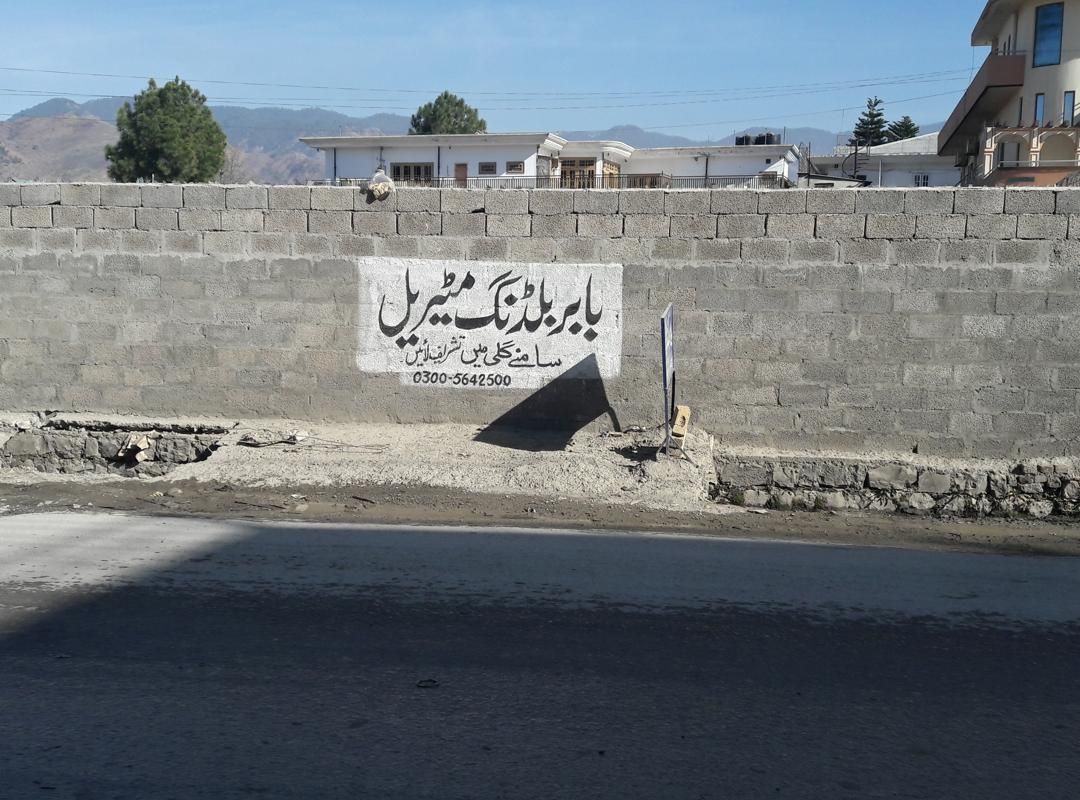

Supplement: Supplemental Information 2 [file peerj-cs-07-717-s002.zip › Testing Dataset/56.jpg]

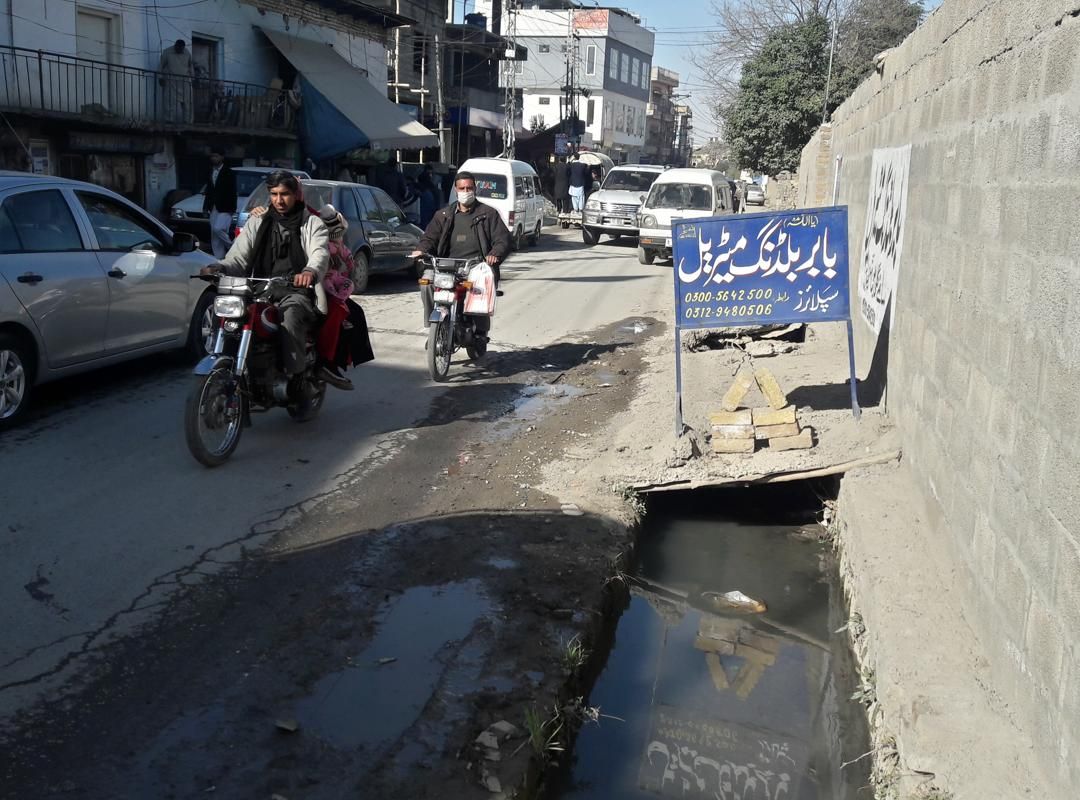

Supplement: Supplemental Information 2 [file peerj-cs-07-717-s002.zip › Testing Dataset/57.jpg]

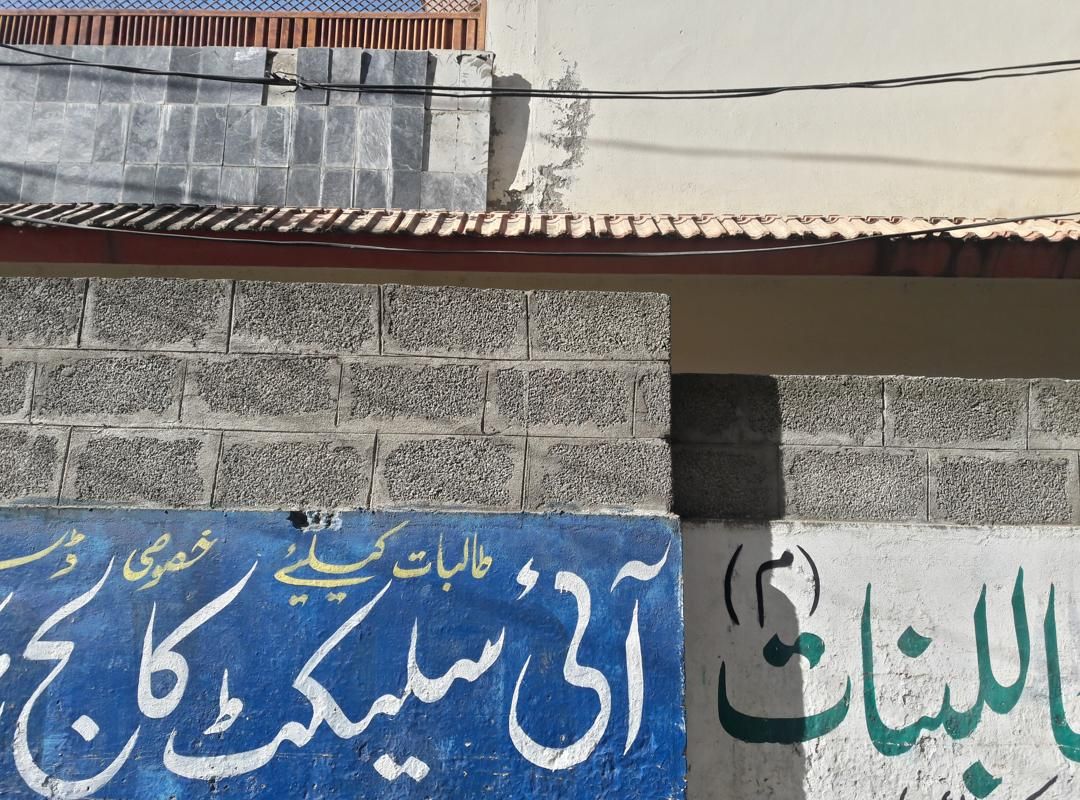

Supplement: Supplemental Information 2 [file peerj-cs-07-717-s002.zip › Testing Dataset/58.jpg]

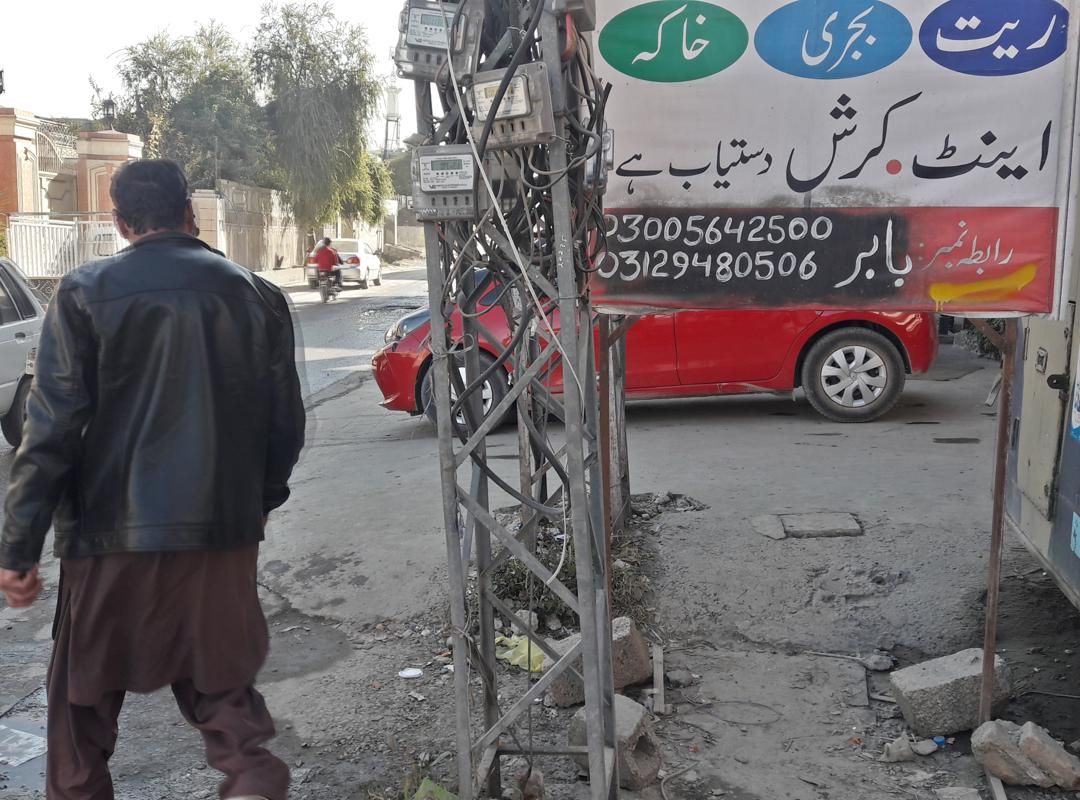

Supplement: Supplemental Information 2 [file peerj-cs-07-717-s002.zip › Testing Dataset/59.jpg]

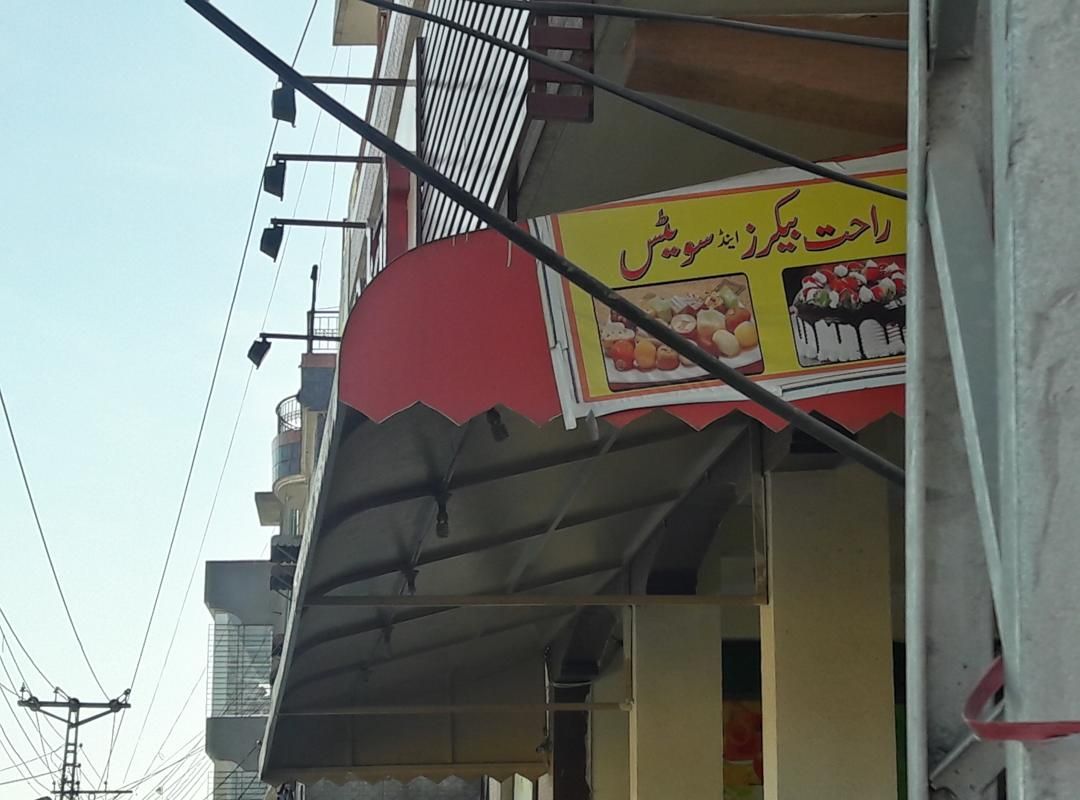

Supplement: Supplemental Information 2 [file peerj-cs-07-717-s002.zip › Testing Dataset/60.jpg]

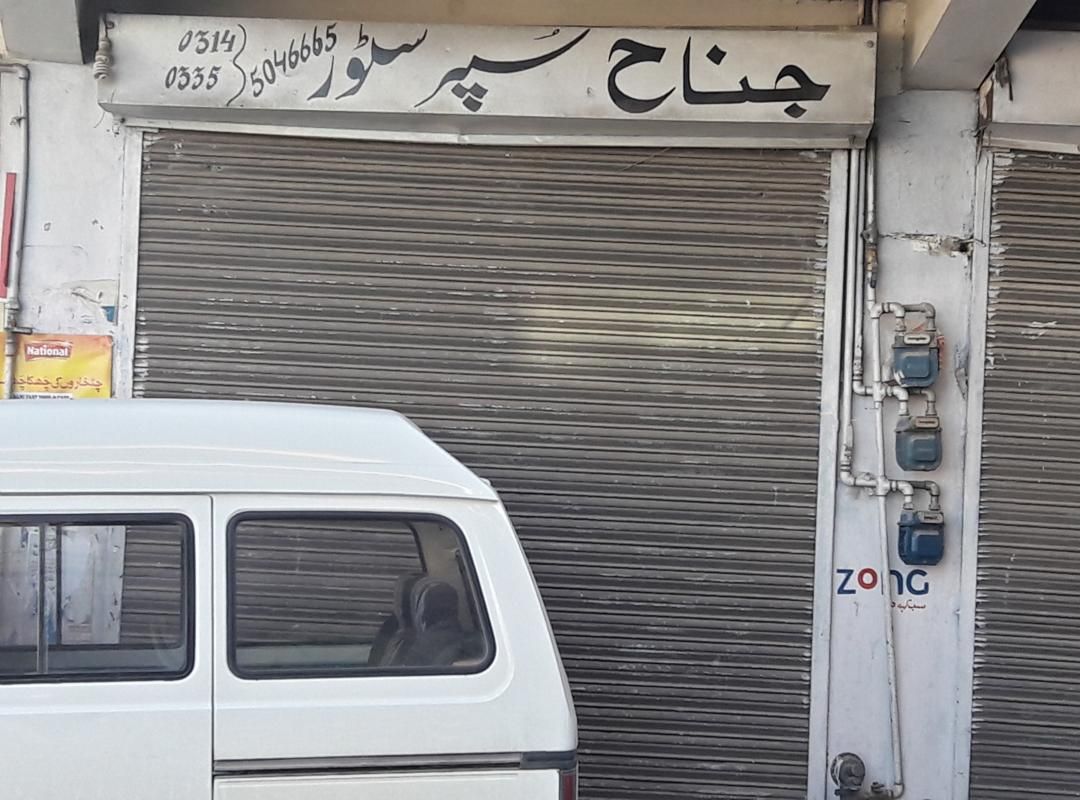

Supplement: Supplemental Information 2 [file peerj-cs-07-717-s002.zip › Testing Dataset/61.jpg]

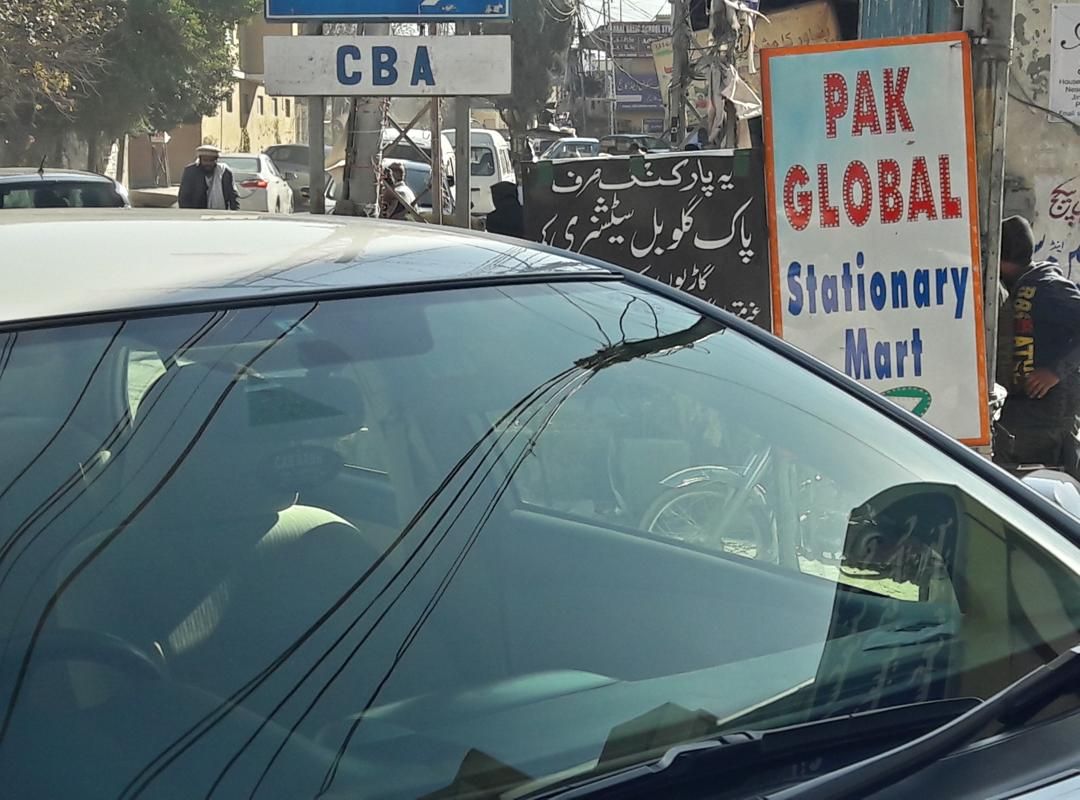

Supplement: Supplemental Information 2 [file peerj-cs-07-717-s002.zip › Testing Dataset/62.jpg]

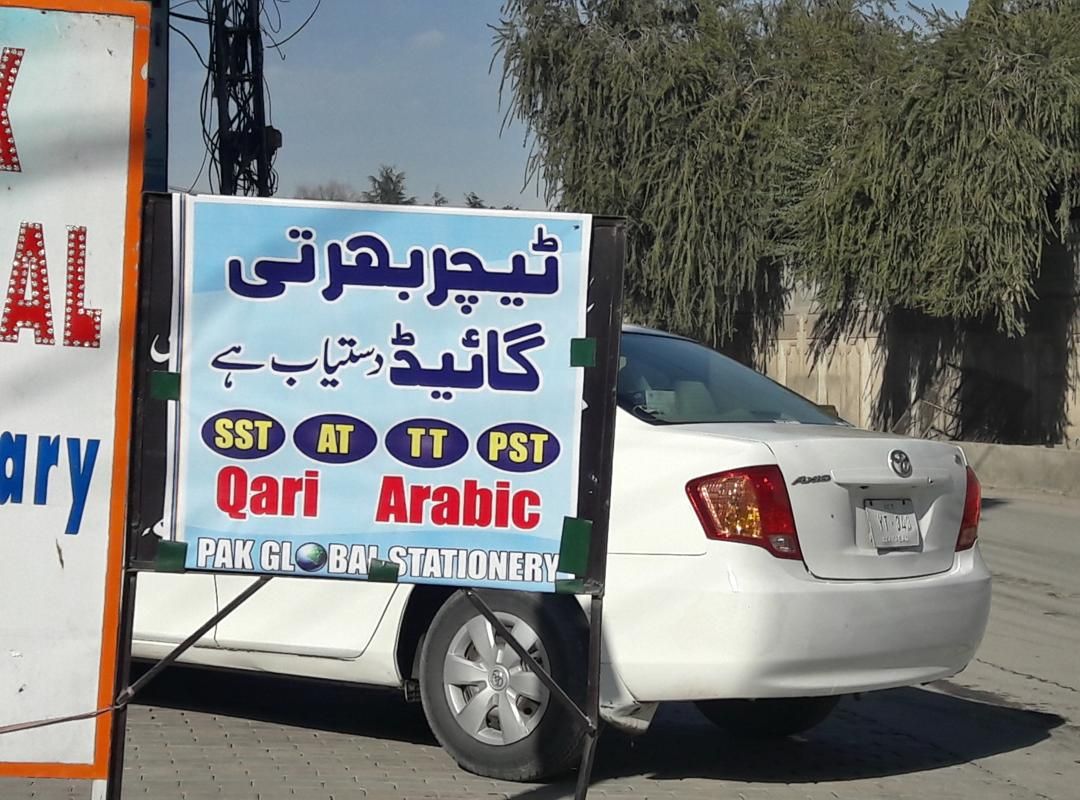

Supplement: Supplemental Information 2 [file peerj-cs-07-717-s002.zip › Testing Dataset/63.jpg]

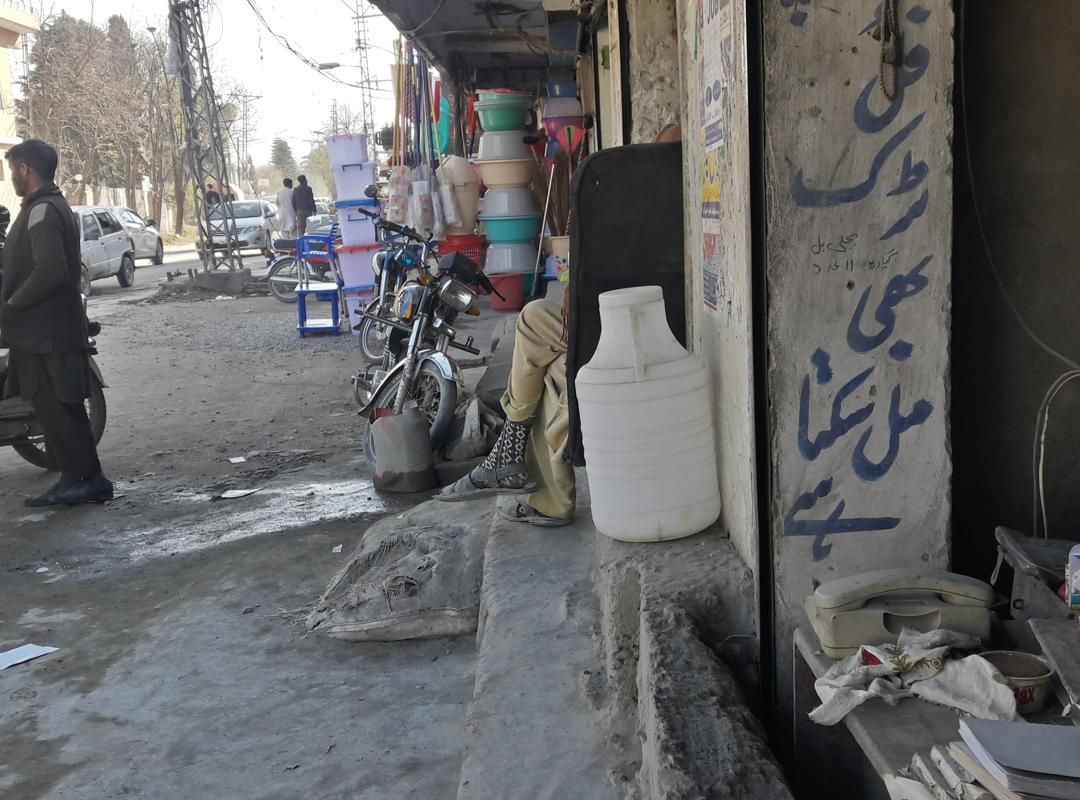

Supplement: Supplemental Information 2 [file peerj-cs-07-717-s002.zip › Testing Dataset/64.jpg]

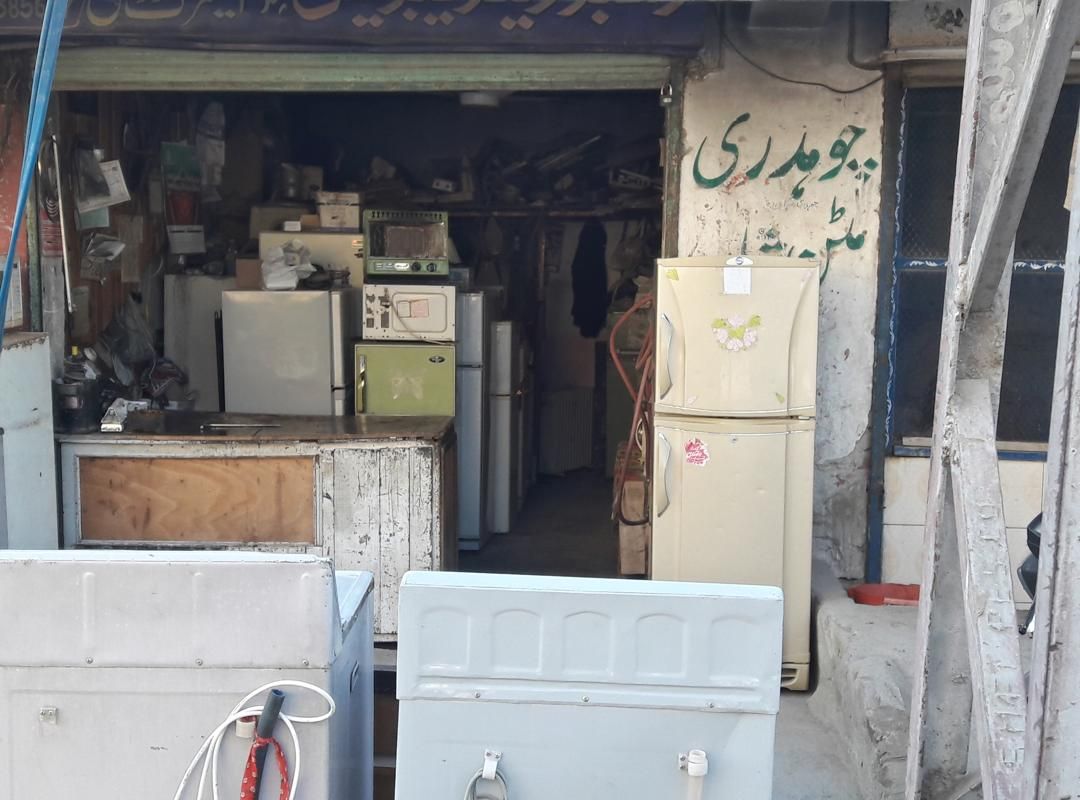

Supplement: Supplemental Information 2 [file peerj-cs-07-717-s002.zip › Testing Dataset/65.jpg]

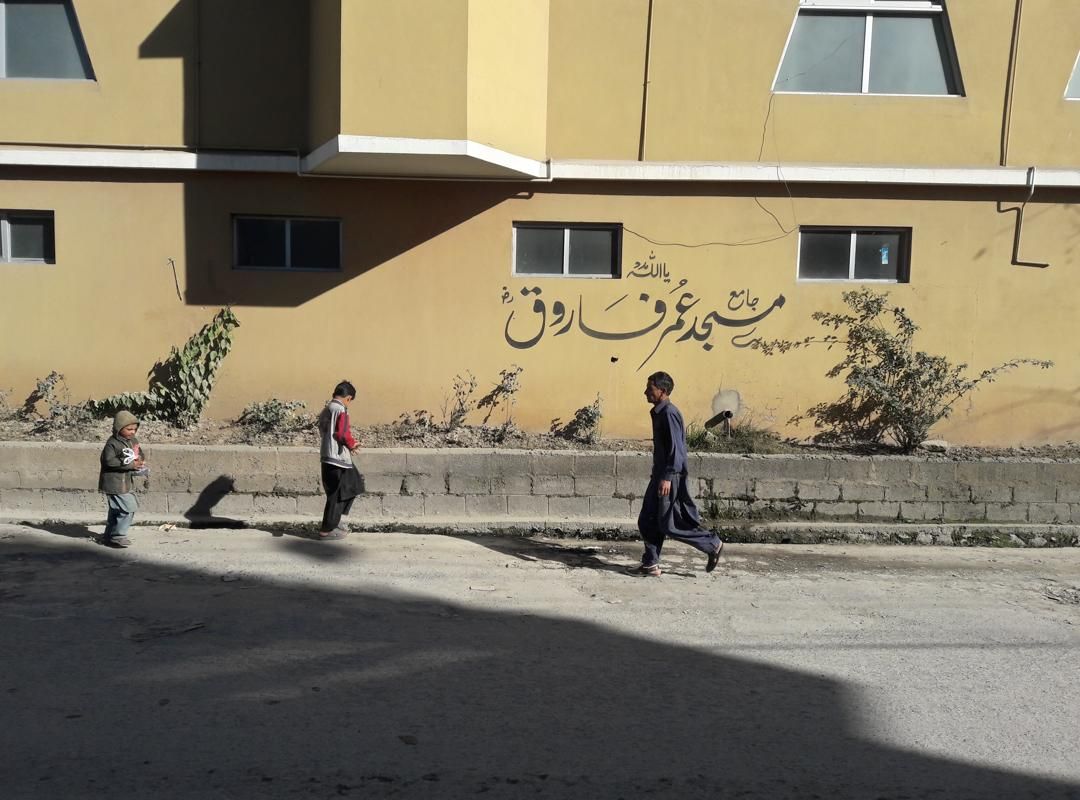

Supplement: Supplemental Information 2 [file peerj-cs-07-717-s002.zip › Testing Dataset/66.jpg]

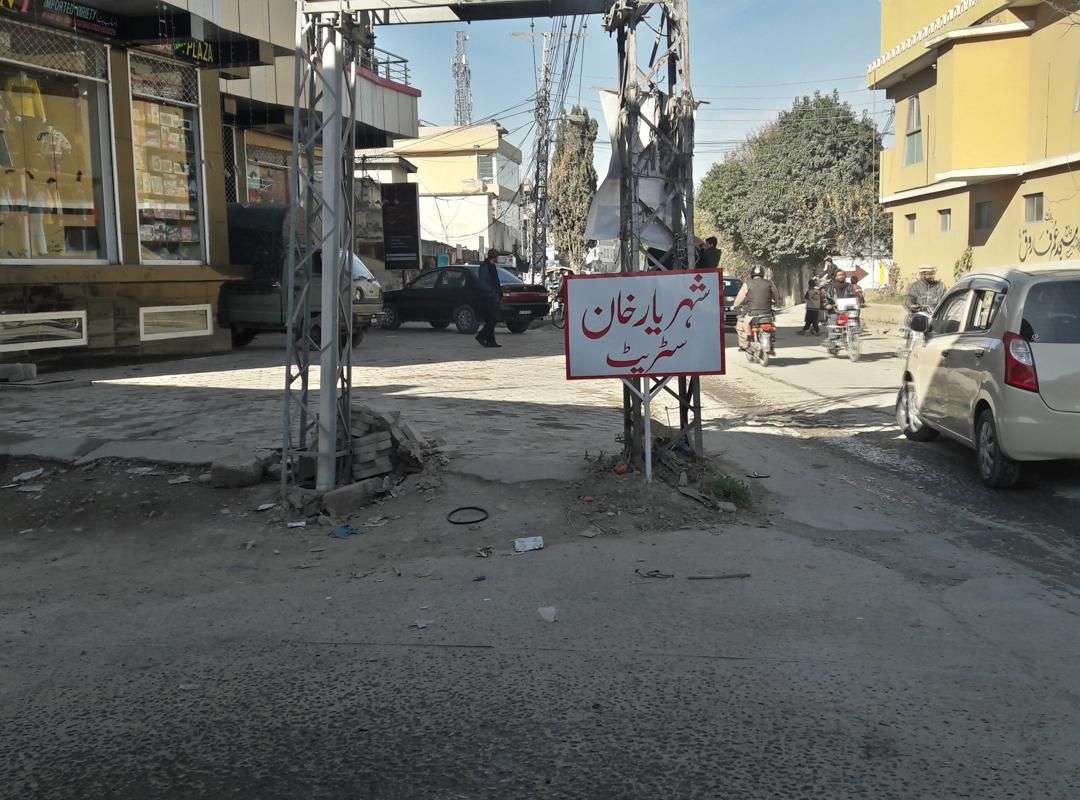

Supplement: Supplemental Information 2 [file peerj-cs-07-717-s002.zip › Testing Dataset/67.jpg]

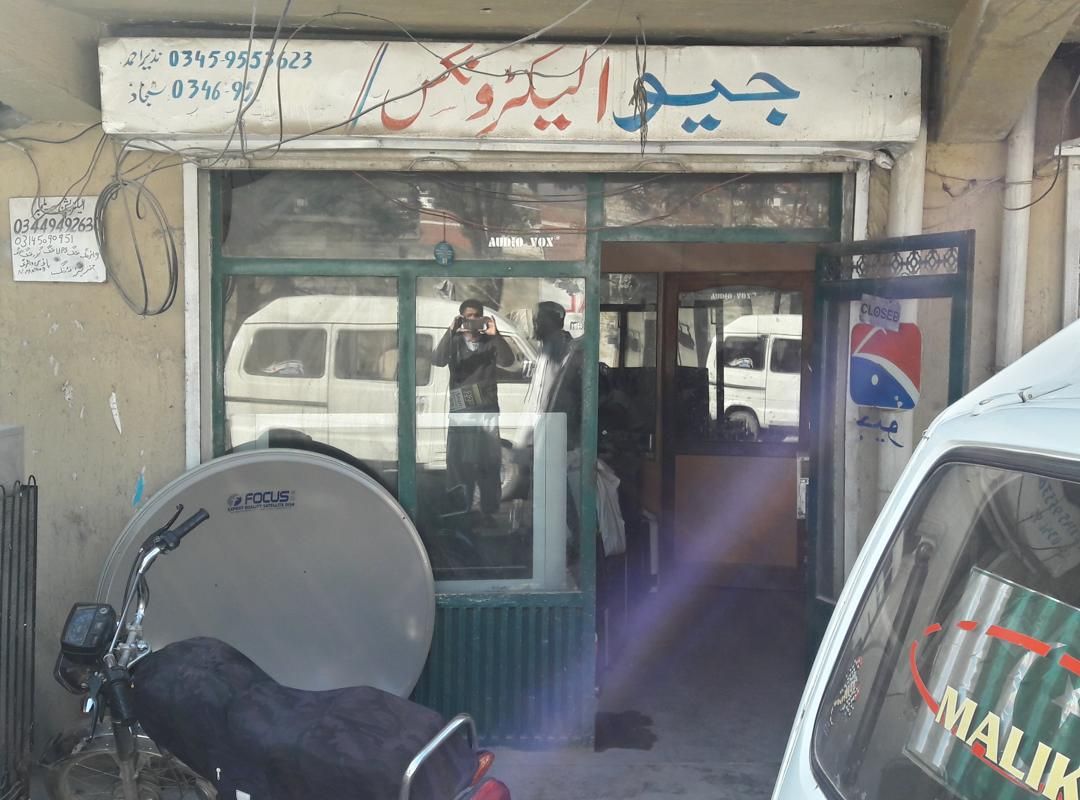

Supplement: Supplemental Information 2 [file peerj-cs-07-717-s002.zip › Testing Dataset/68.jpg]

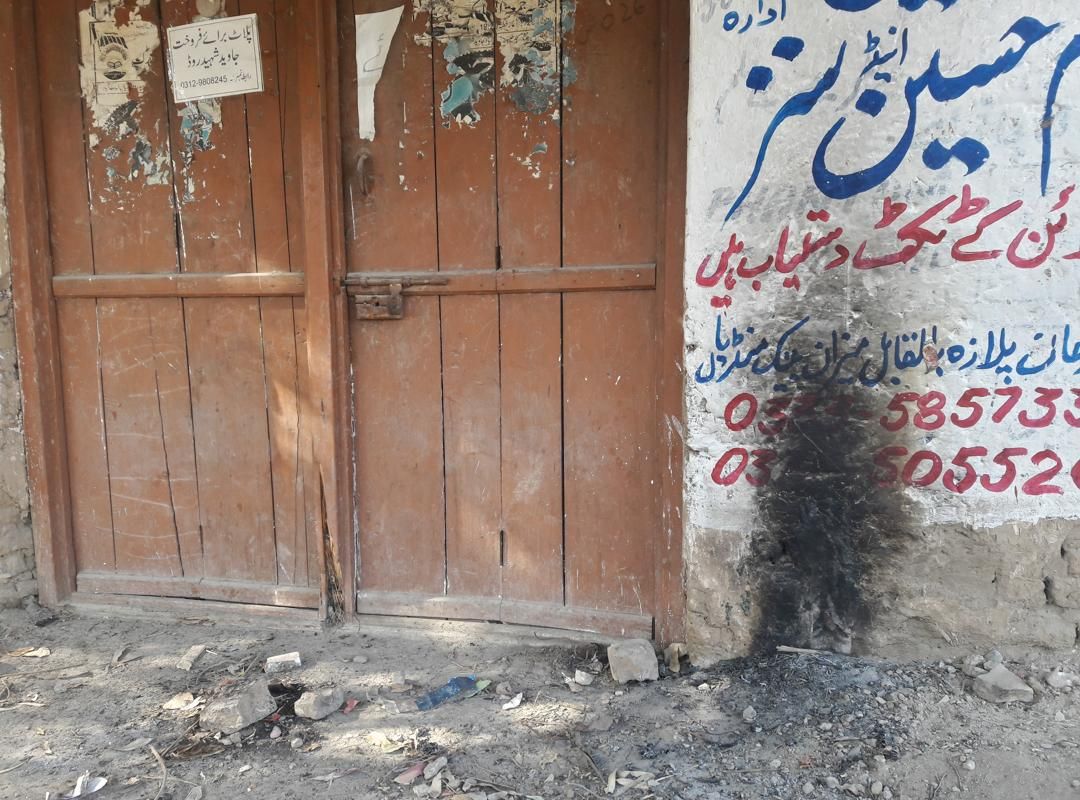

Supplement: Supplemental Information 2 [file peerj-cs-07-717-s002.zip › Testing Dataset/69.jpg]

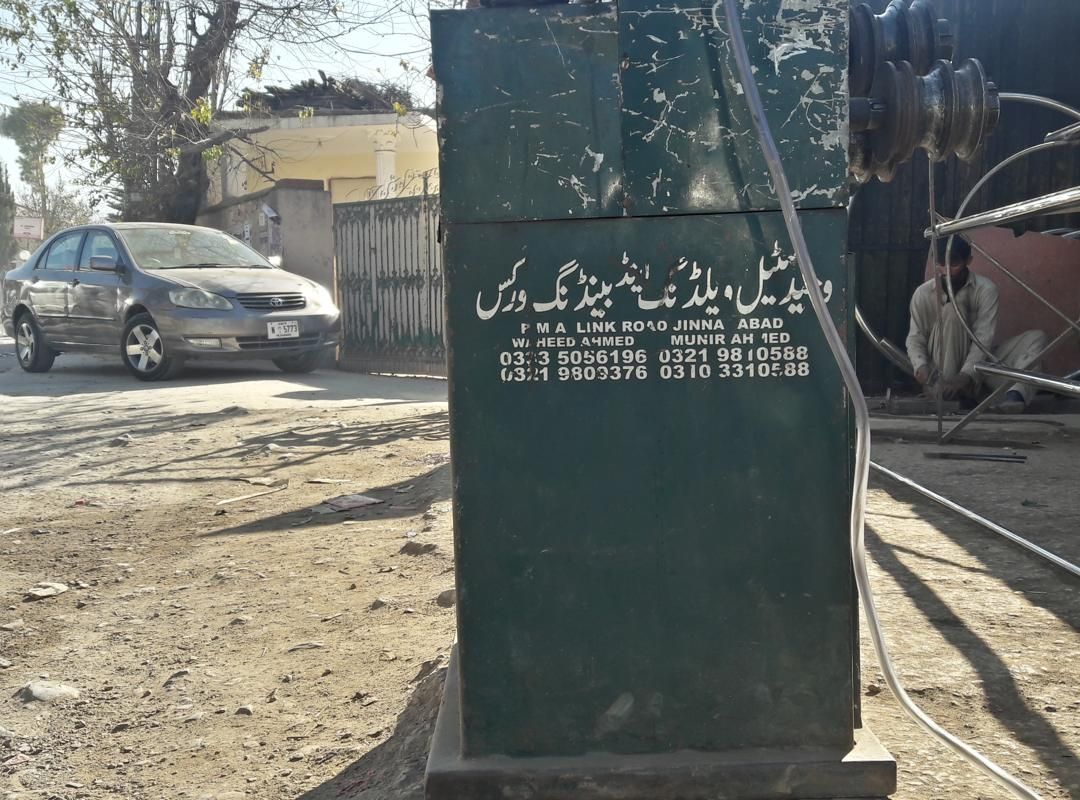

Supplement: Supplemental Information 2 [file peerj-cs-07-717-s002.zip › Testing Dataset/70.jpg]

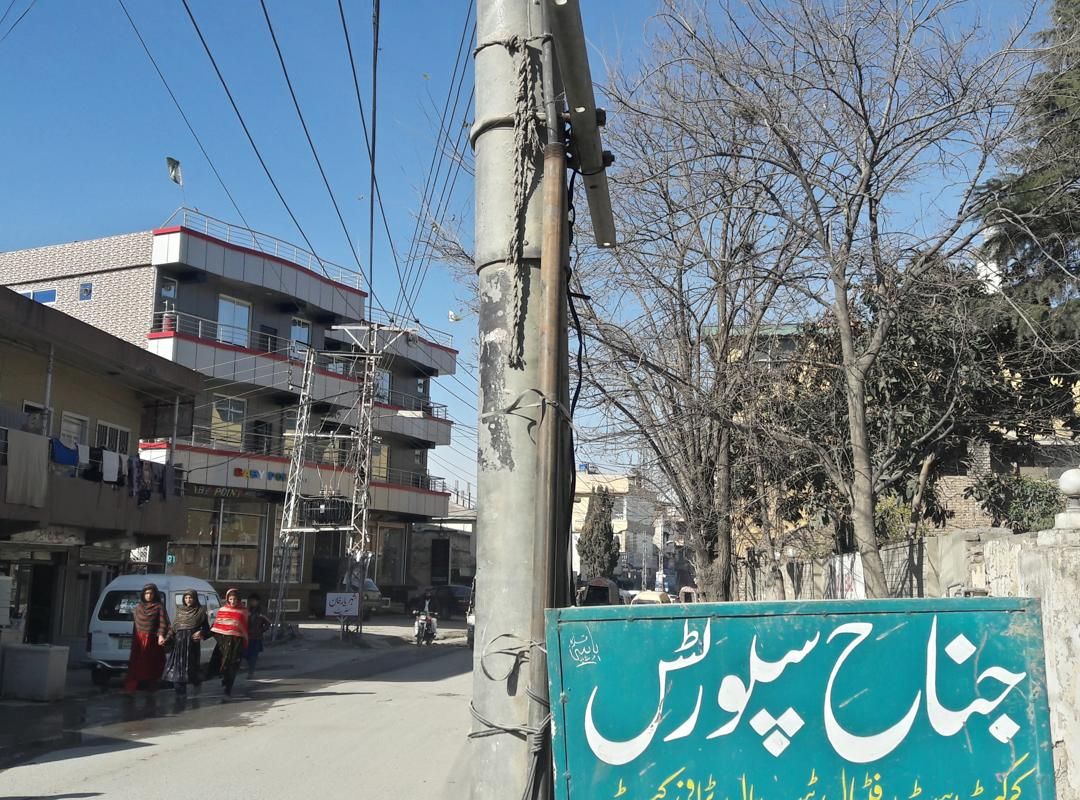

Supplement: Supplemental Information 2 [file peerj-cs-07-717-s002.zip › Testing Dataset/71.jpg]

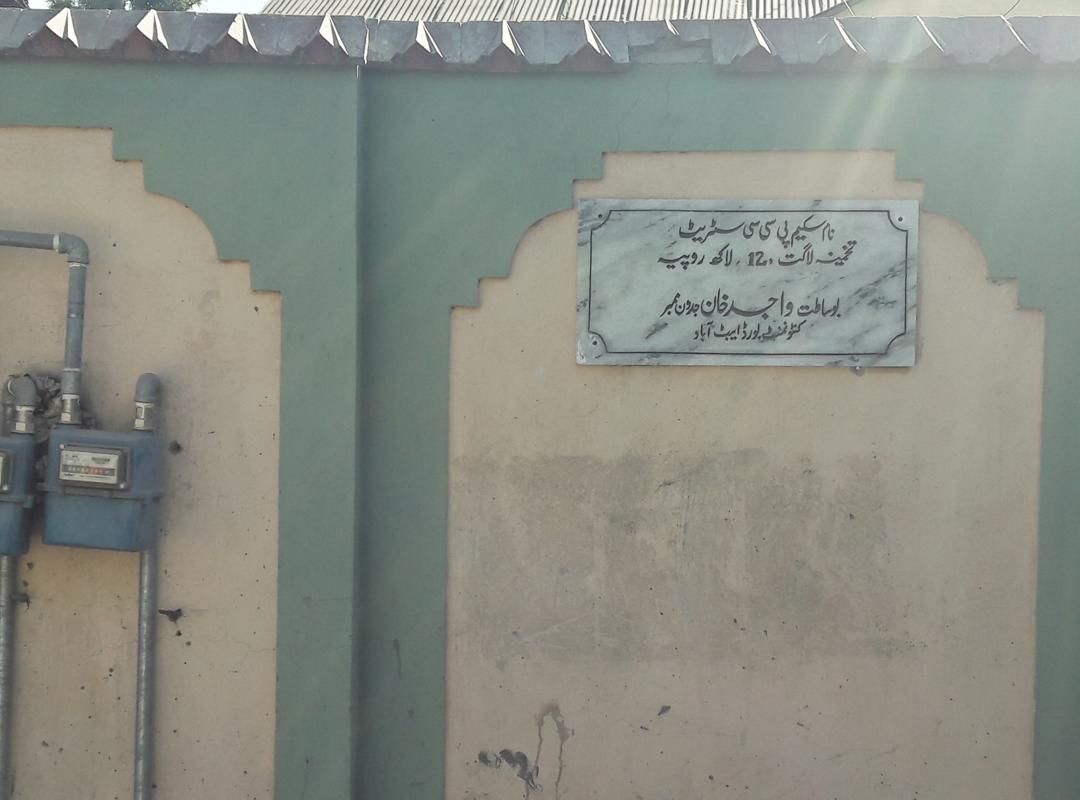

Supplement: Supplemental Information 2 [file peerj-cs-07-717-s002.zip › Testing Dataset/72.jpg]

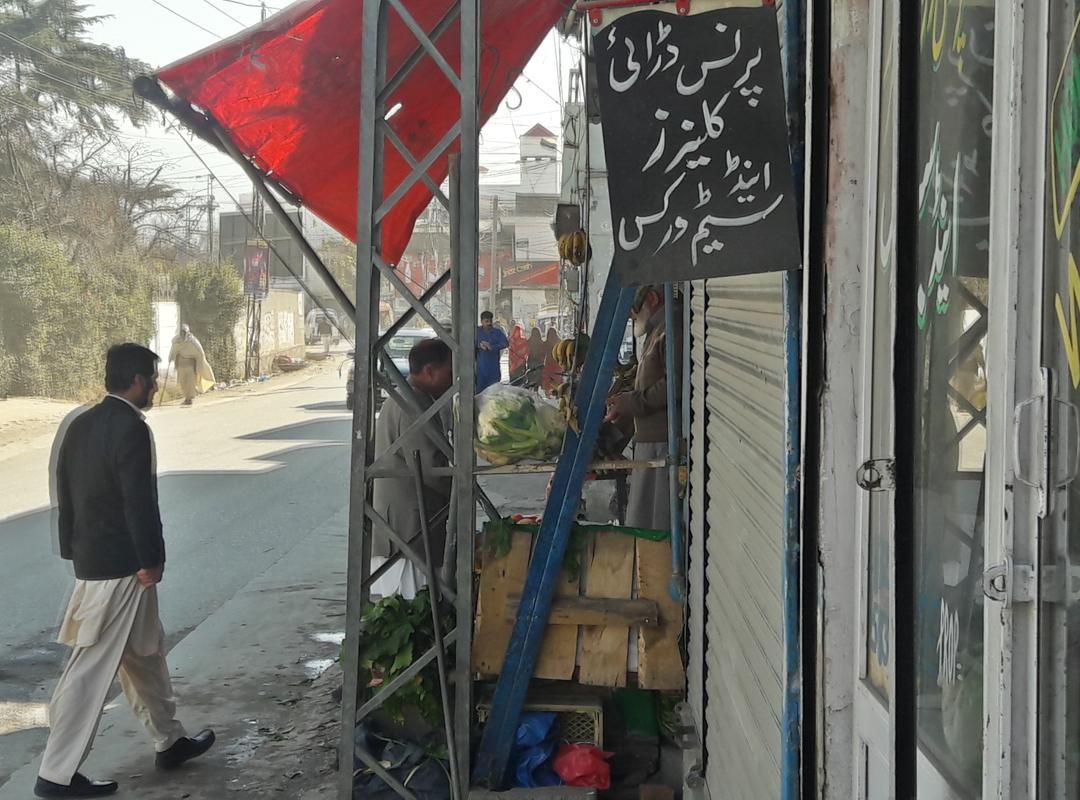

Supplement: Supplemental Information 2 [file peerj-cs-07-717-s002.zip › Testing Dataset/73.jpg]

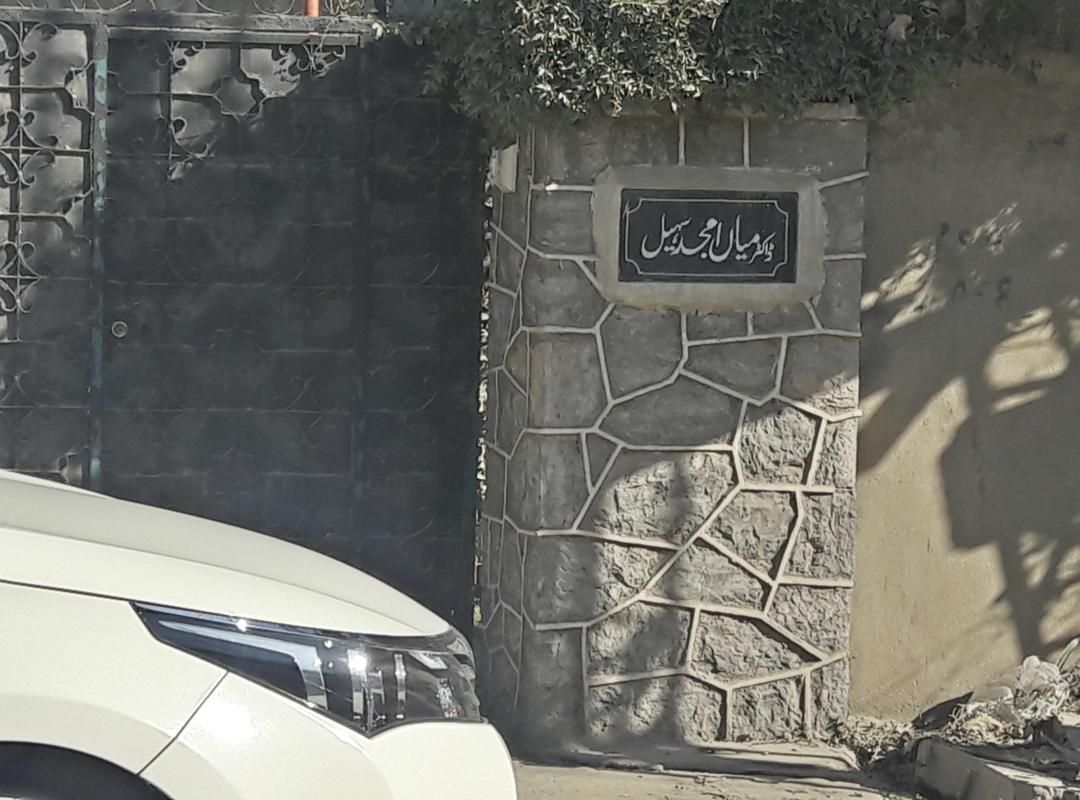

Supplement: Supplemental Information 2 [file peerj-cs-07-717-s002.zip › Testing Dataset/74.jpg]

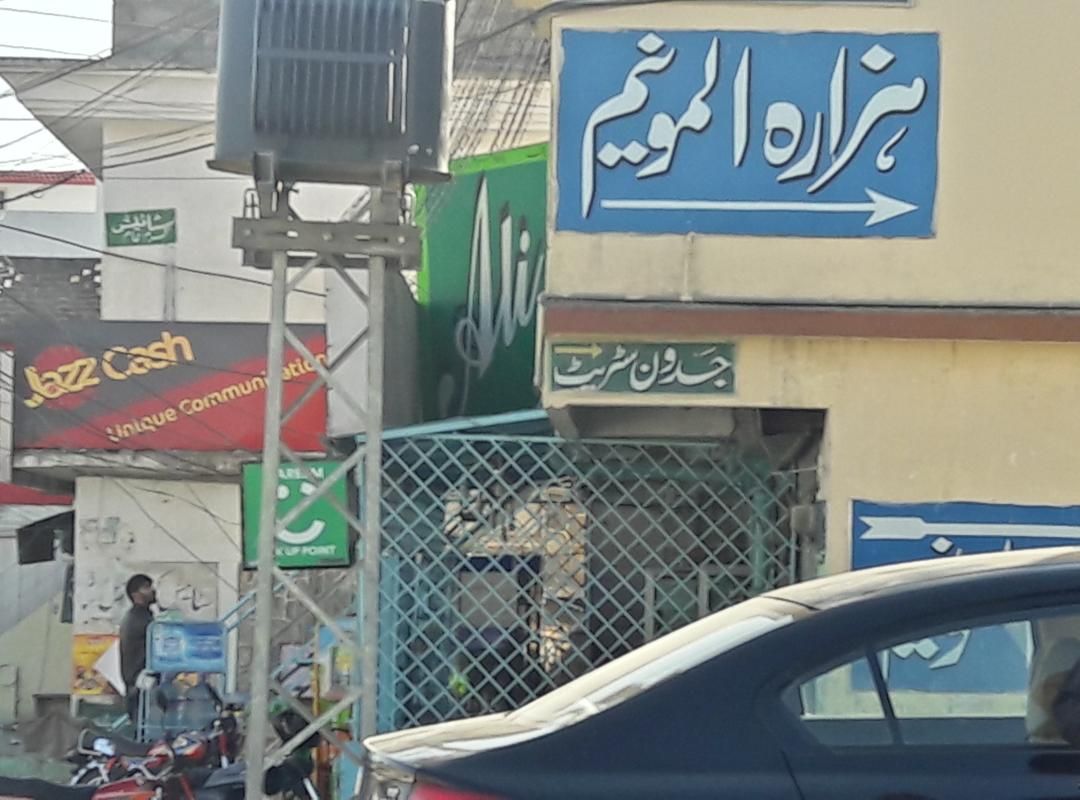

Supplement: Supplemental Information 2 [file peerj-cs-07-717-s002.zip › Testing Dataset/75.jpg]

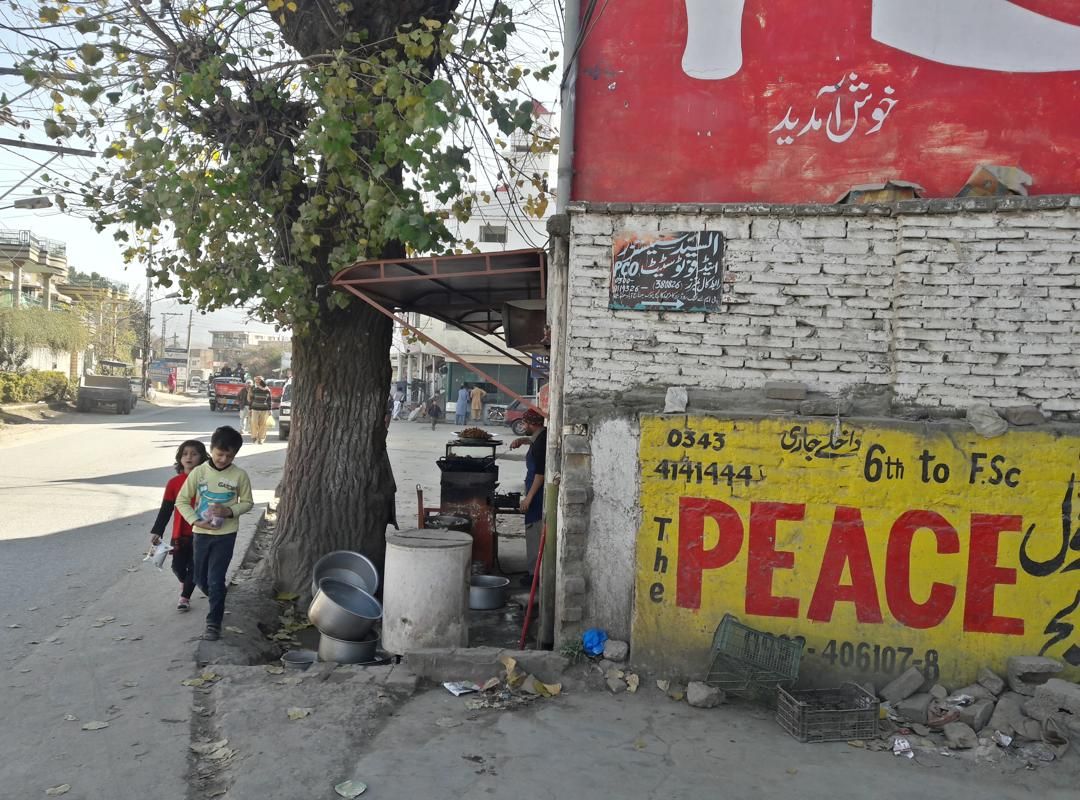

Supplement: Supplemental Information 2 [file peerj-cs-07-717-s002.zip › Testing Dataset/76.jpg]

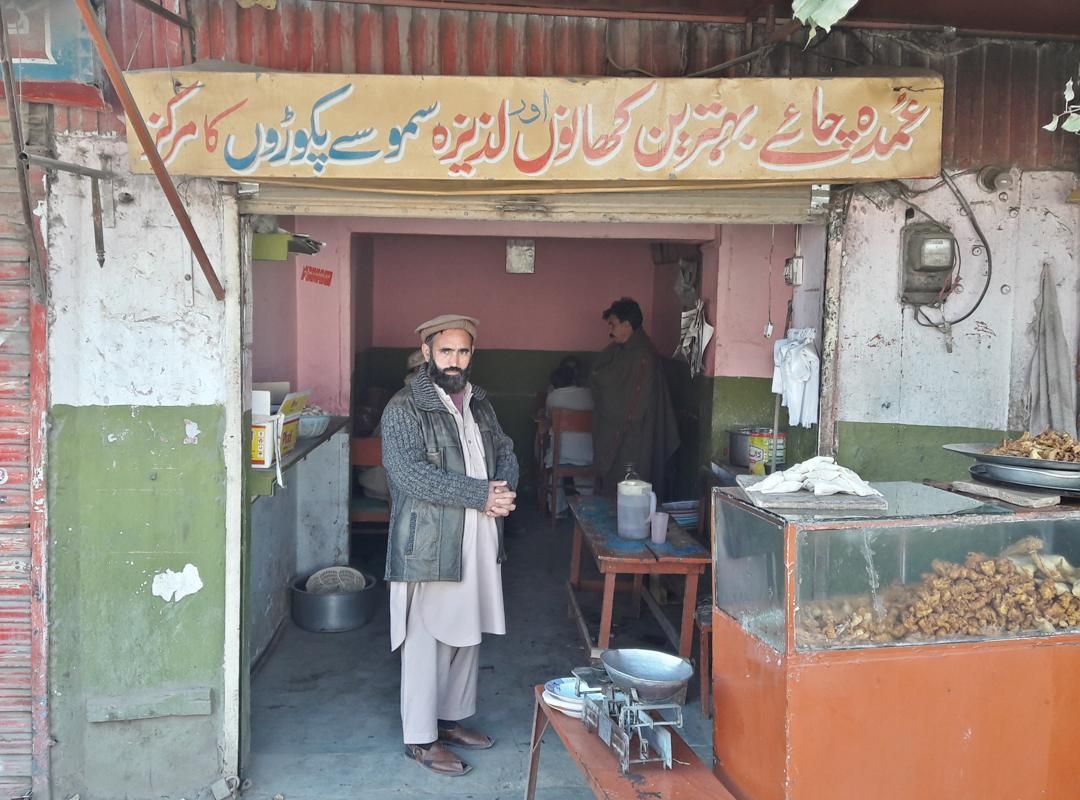

Supplement: Supplemental Information 2 [file peerj-cs-07-717-s002.zip › Testing Dataset/77.jpg]

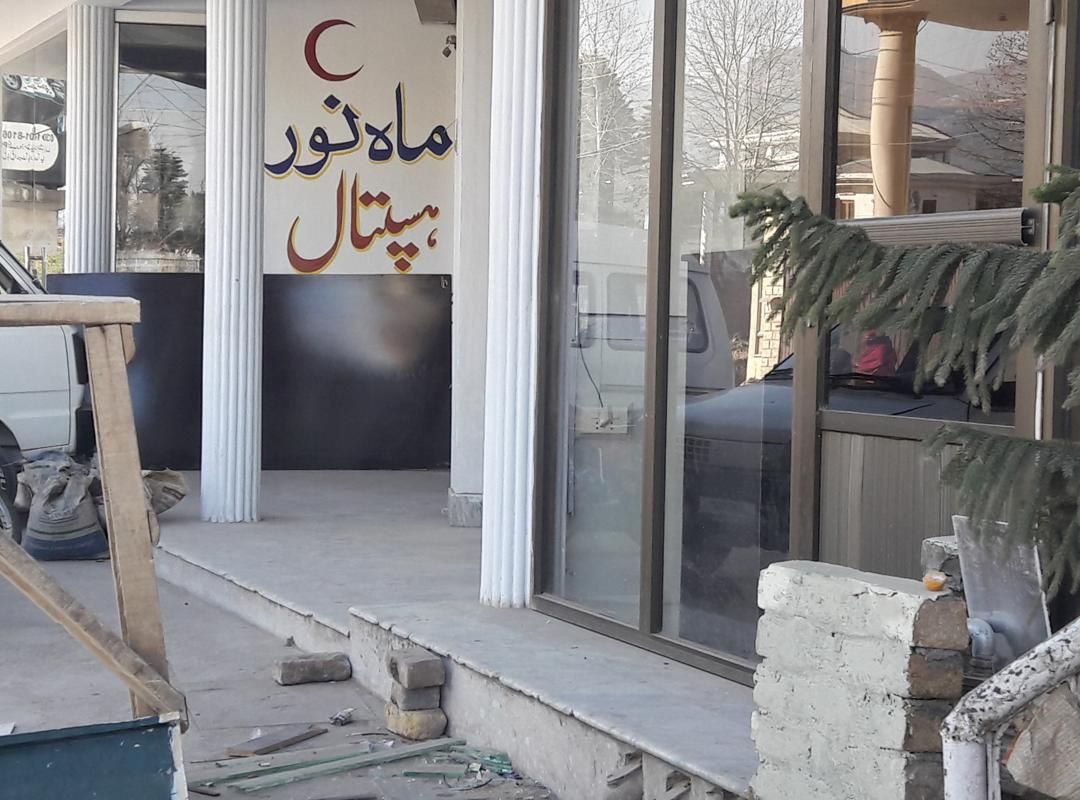

Supplement: Supplemental Information 2 [file peerj-cs-07-717-s002.zip › Testing Dataset/78.jpg]

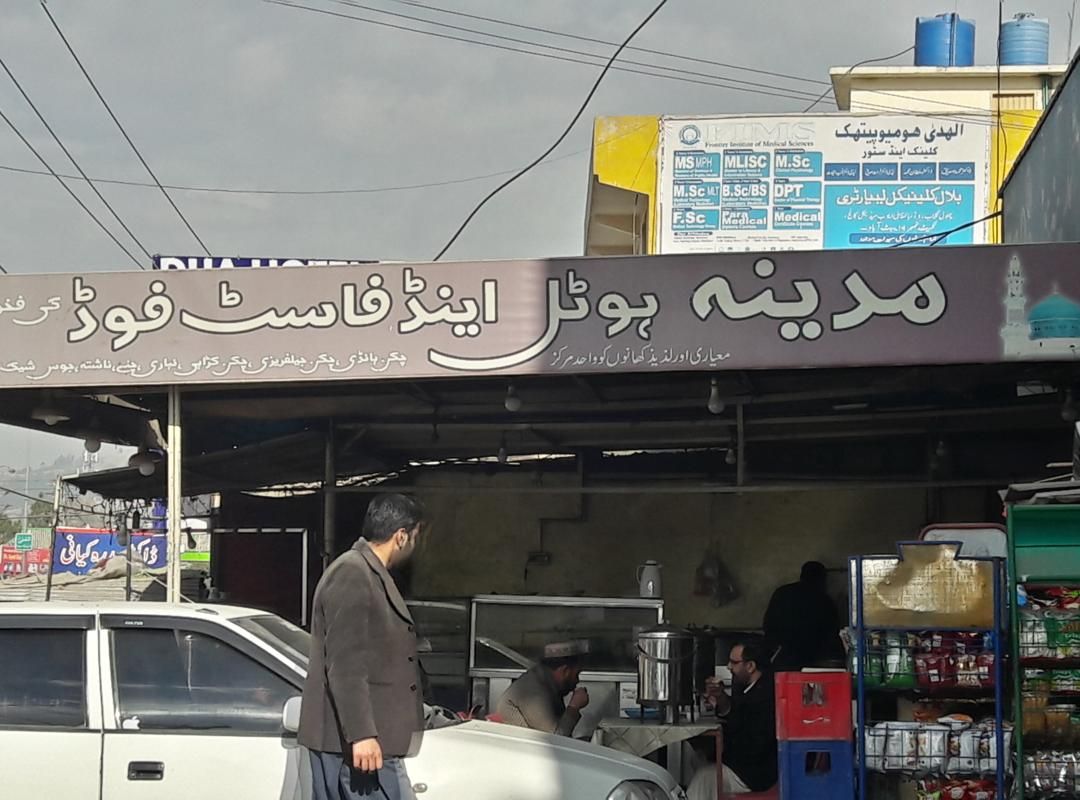

Supplement: Supplemental Information 2 [file peerj-cs-07-717-s002.zip › Testing Dataset/79.jpg]

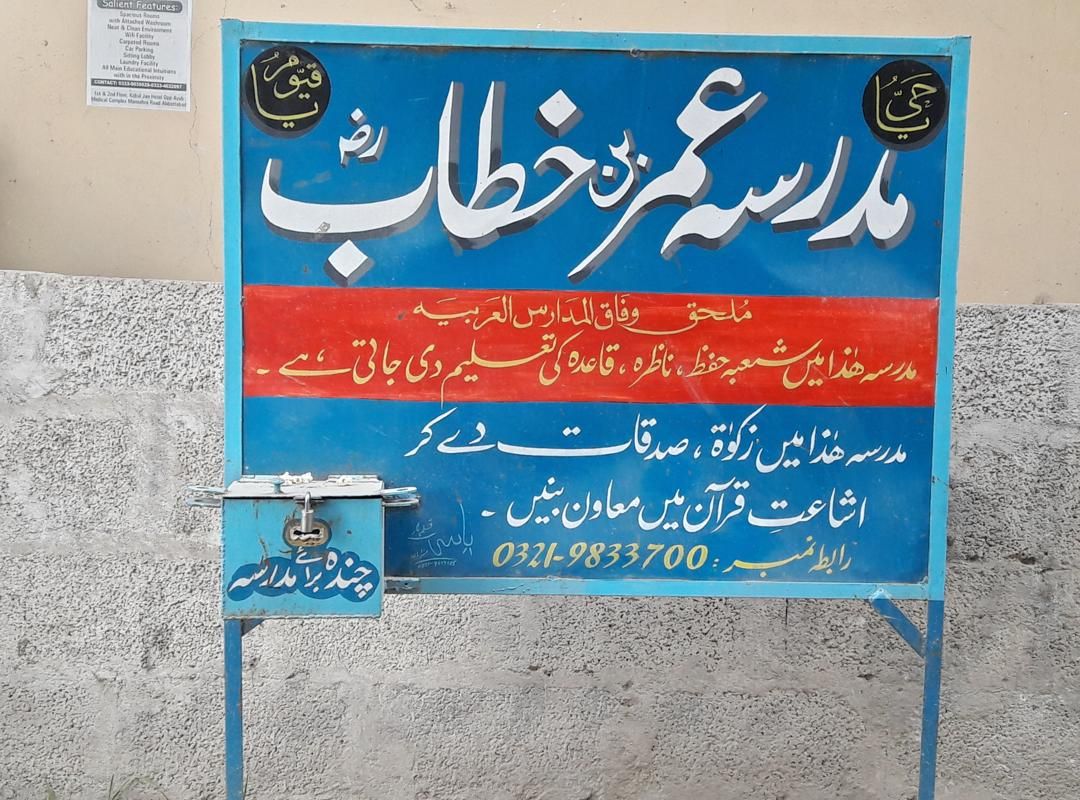

Supplement: Supplemental Information 2 [file peerj-cs-07-717-s002.zip › Testing Dataset/80.jpg]

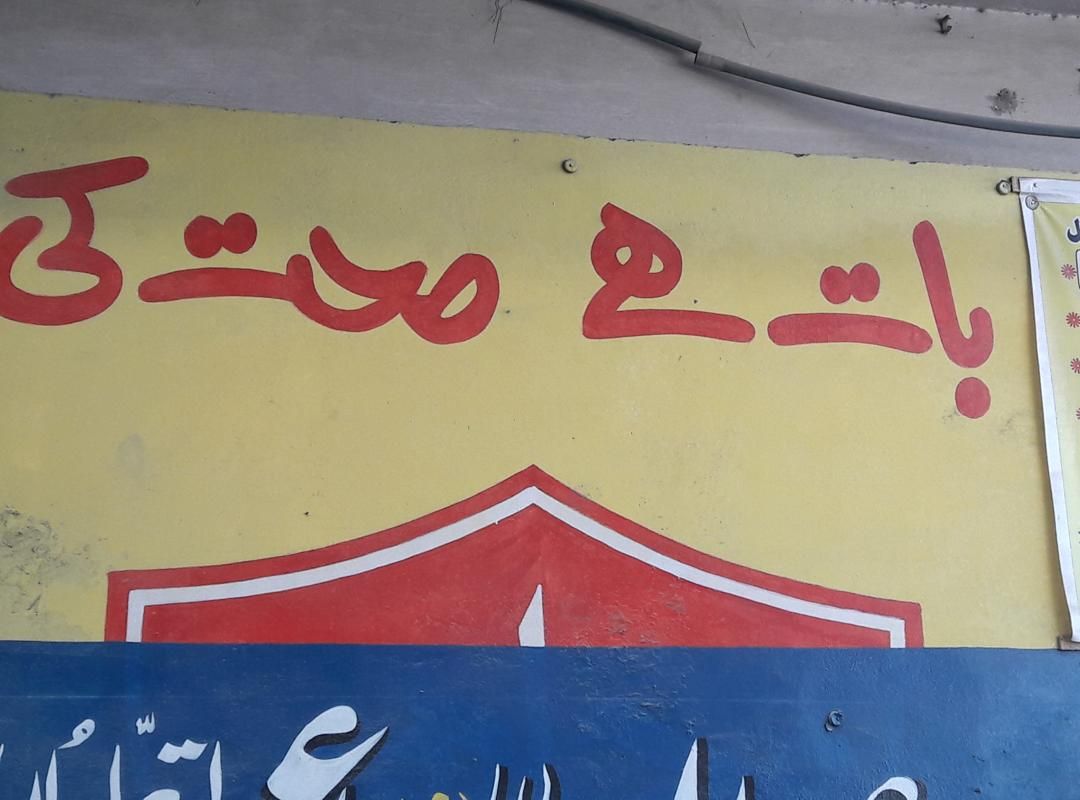

Supplement: Supplemental Information 2 [file peerj-cs-07-717-s002.zip › Testing Dataset/81.jpg]

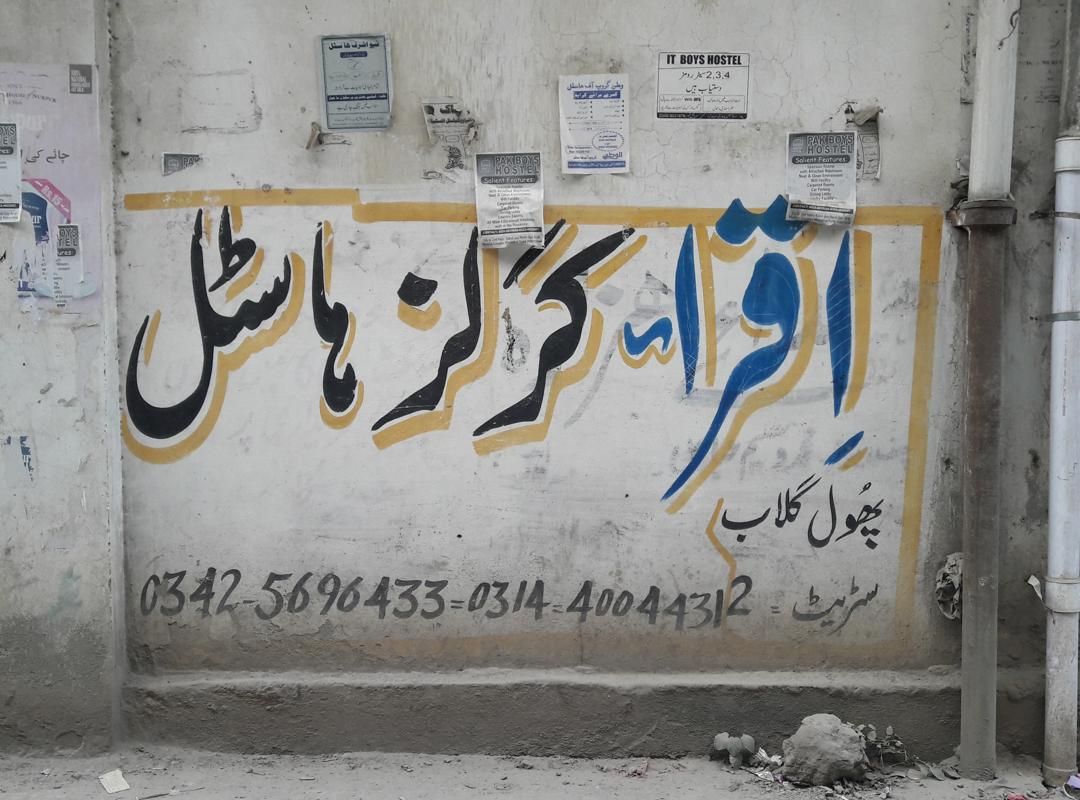

Supplement: Supplemental Information 2 [file peerj-cs-07-717-s002.zip › Testing Dataset/82.jpg]

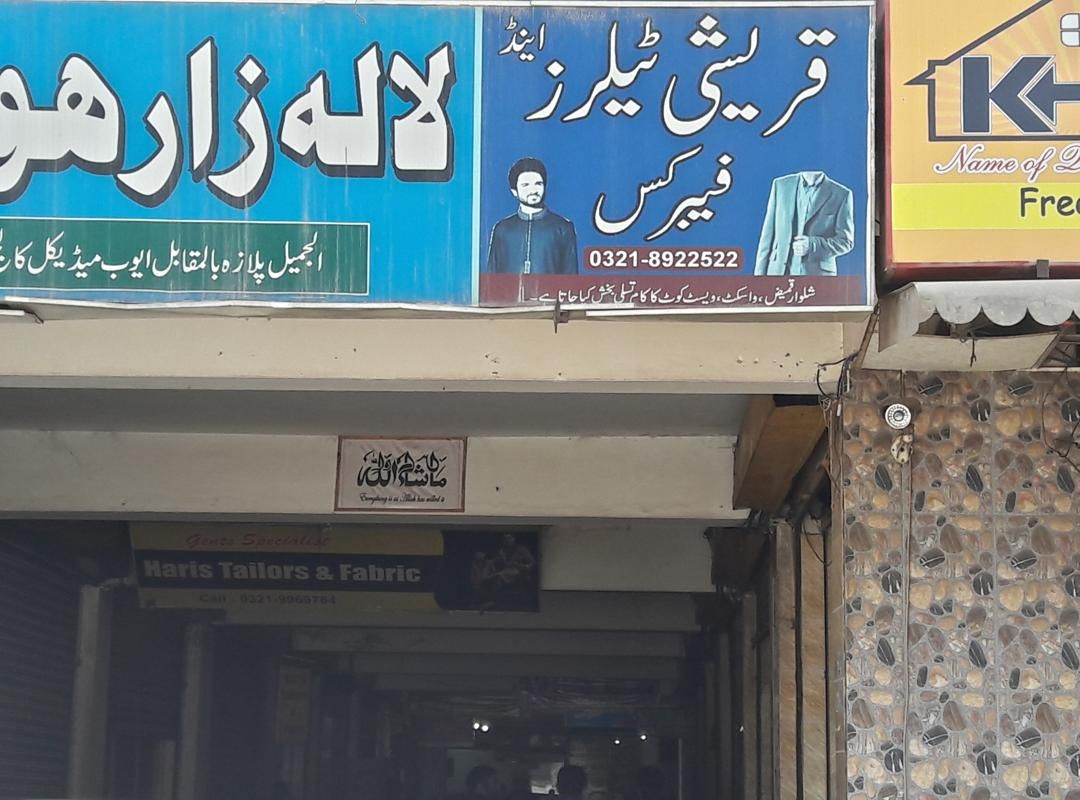

Supplement: Supplemental Information 2 [file peerj-cs-07-717-s002.zip › Testing Dataset/83.jpg]

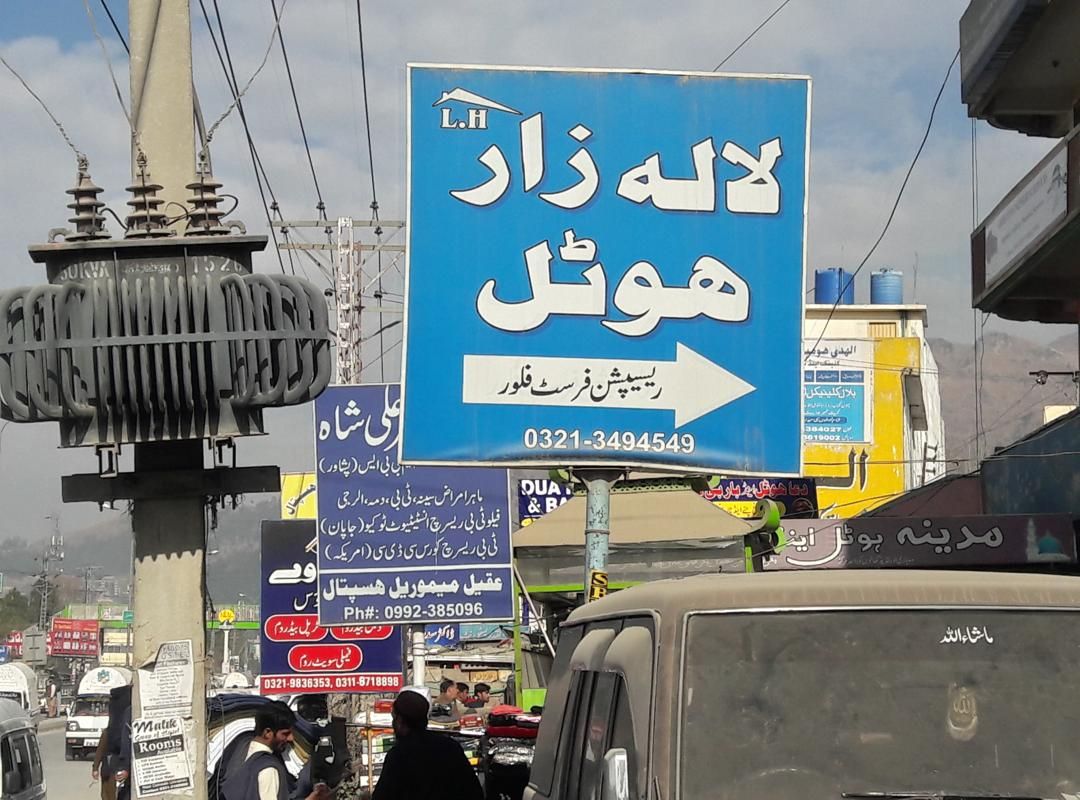

Supplement: Supplemental Information 2 [file peerj-cs-07-717-s002.zip › Testing Dataset/84.jpg]

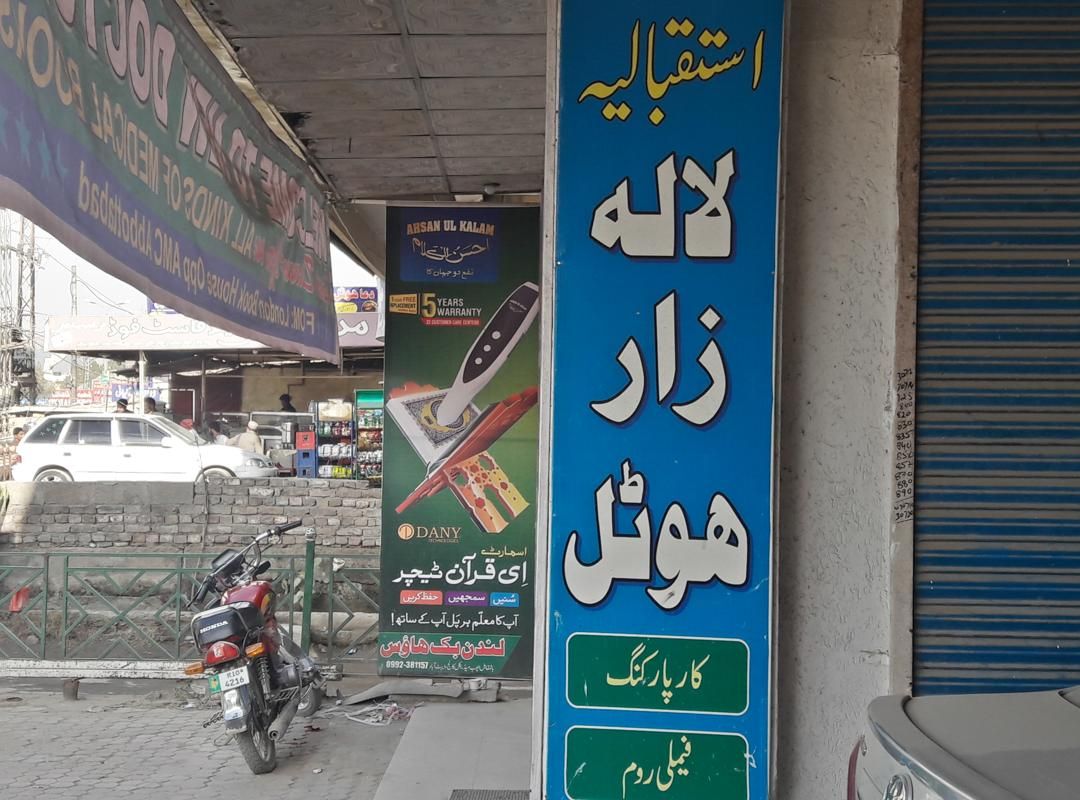

Supplement: Supplemental Information 2 [file peerj-cs-07-717-s002.zip › Testing Dataset/85.jpg]

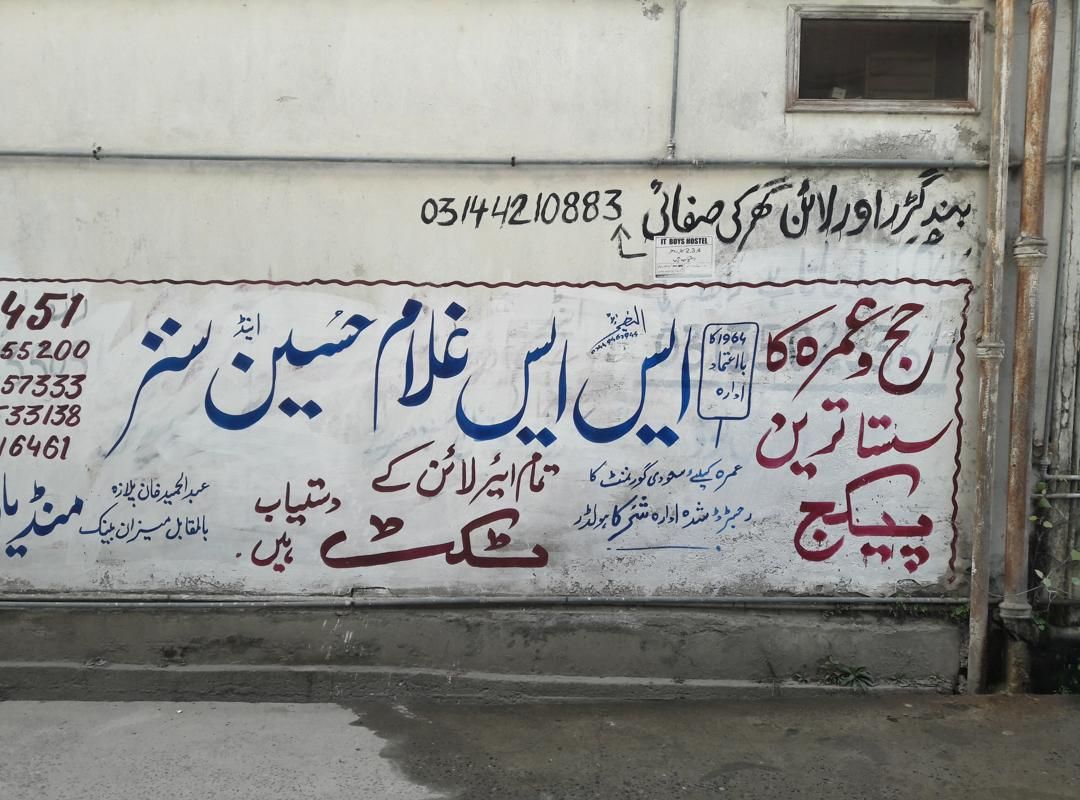

Supplement: Supplemental Information 2 [file peerj-cs-07-717-s002.zip › Testing Dataset/86.jpg]

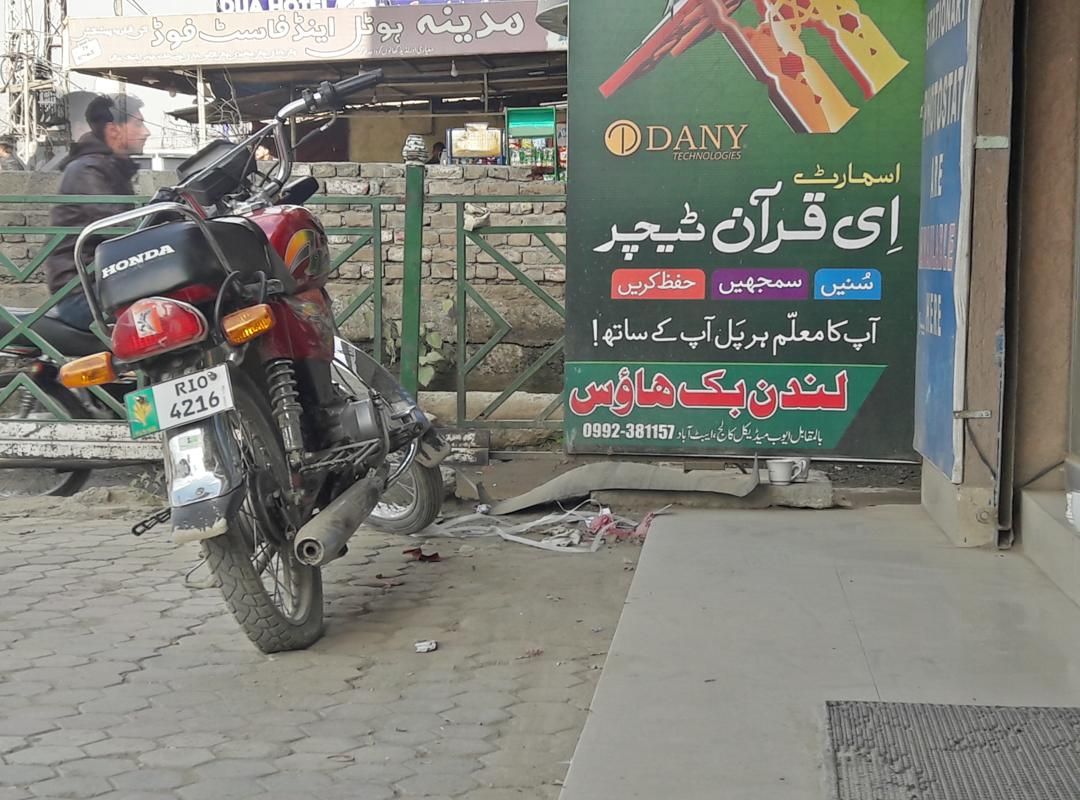

Supplement: Supplemental Information 2 [file peerj-cs-07-717-s002.zip › Testing Dataset/87.jpg]

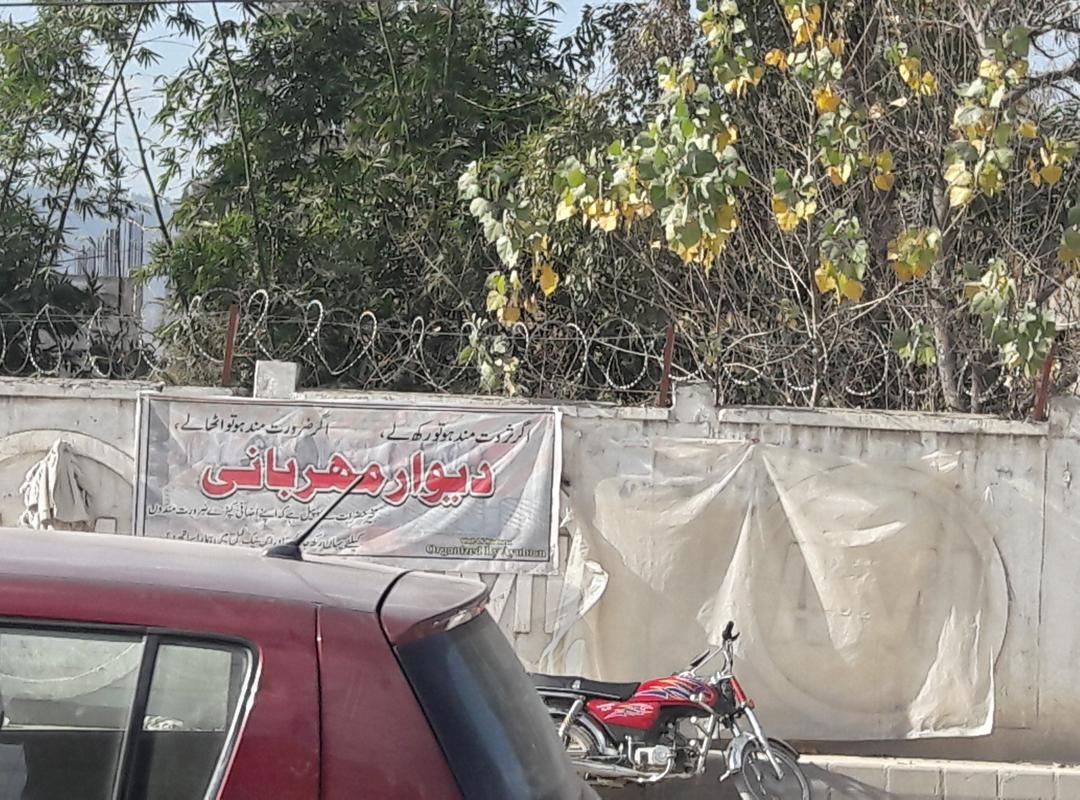

Supplement: Supplemental Information 2 [file peerj-cs-07-717-s002.zip › Testing Dataset/88.jpg]

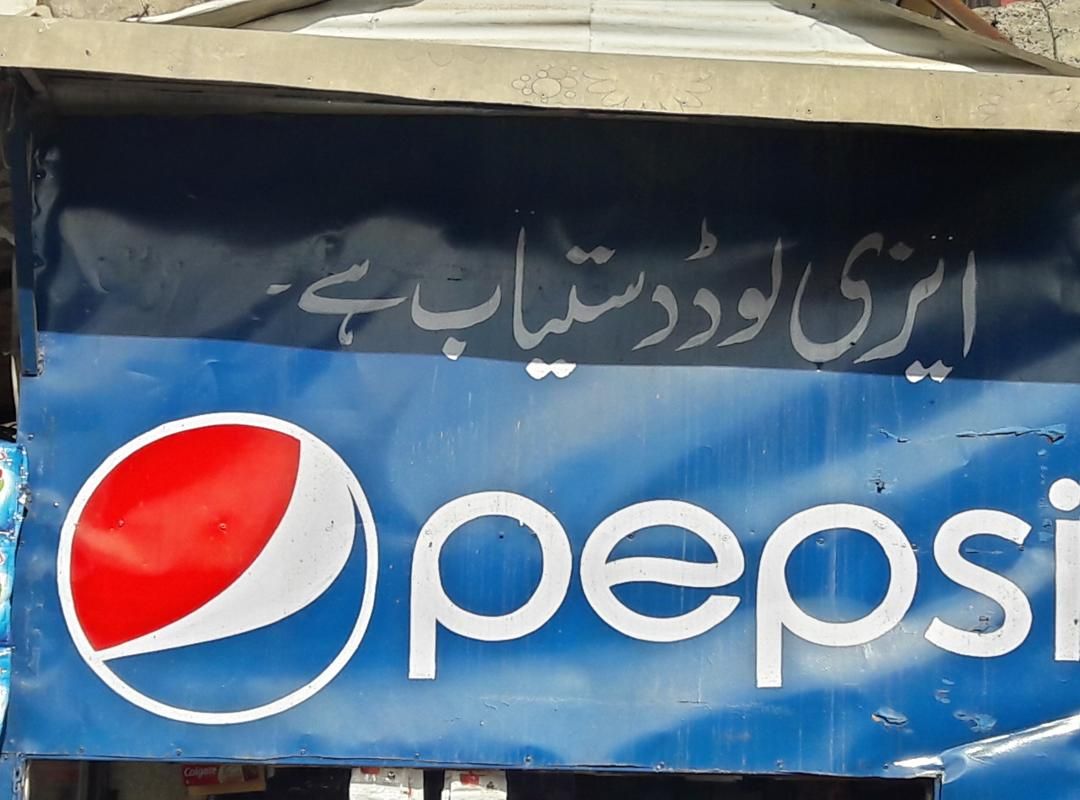

Supplement: Supplemental Information 2 [file peerj-cs-07-717-s002.zip › Testing Dataset/89.jpg]

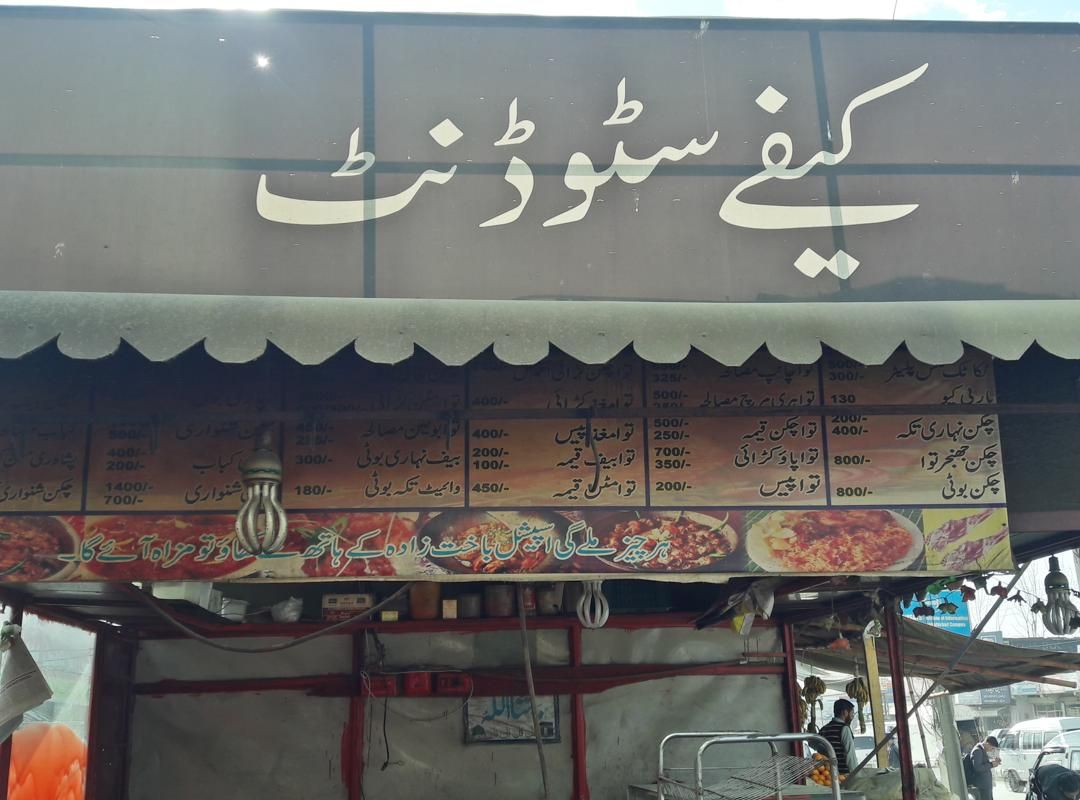

Supplement: Supplemental Information 2 [file peerj-cs-07-717-s002.zip › Testing Dataset/90.jpg]

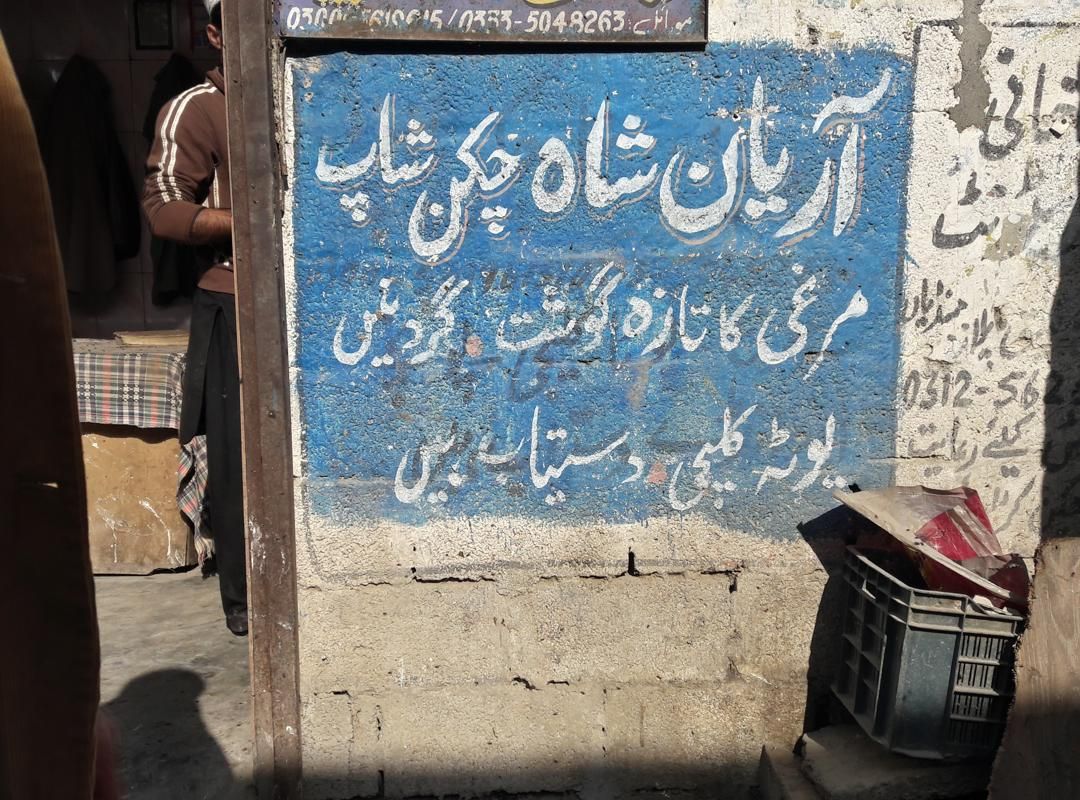

Supplement: Supplemental Information 2 [file peerj-cs-07-717-s002.zip › Testing Dataset/91.jpg]

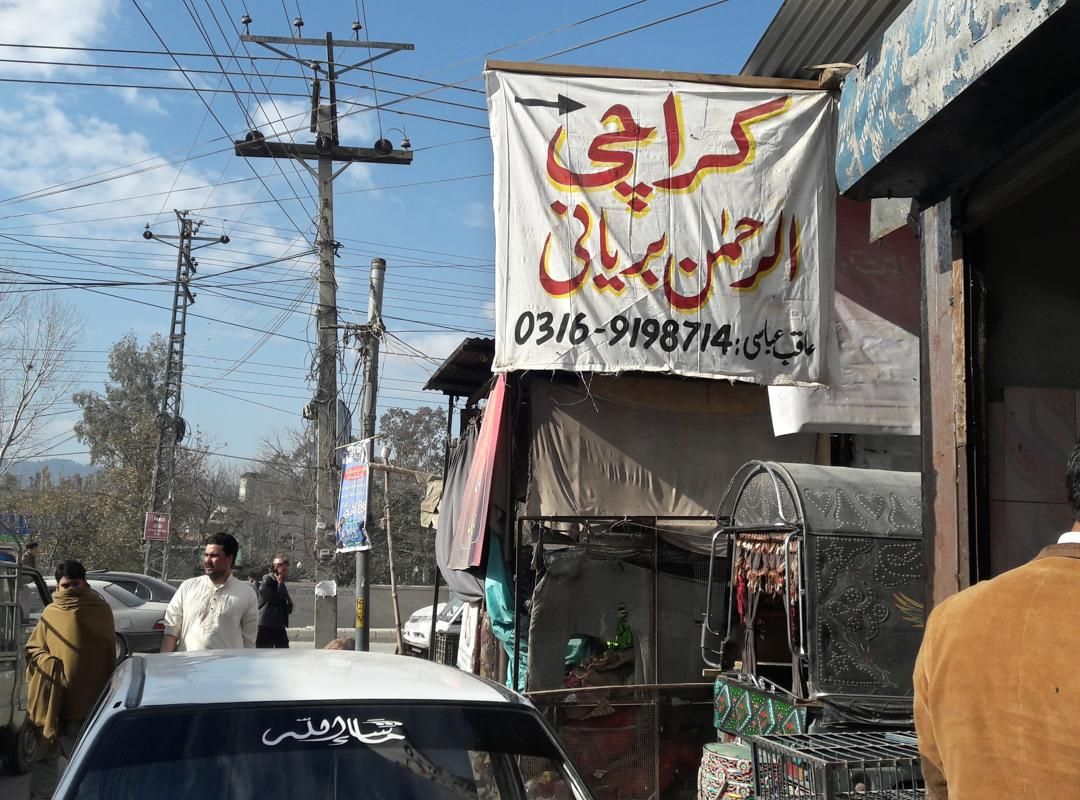

Supplement: Supplemental Information 2 [file peerj-cs-07-717-s002.zip › Testing Dataset/92.jpg]

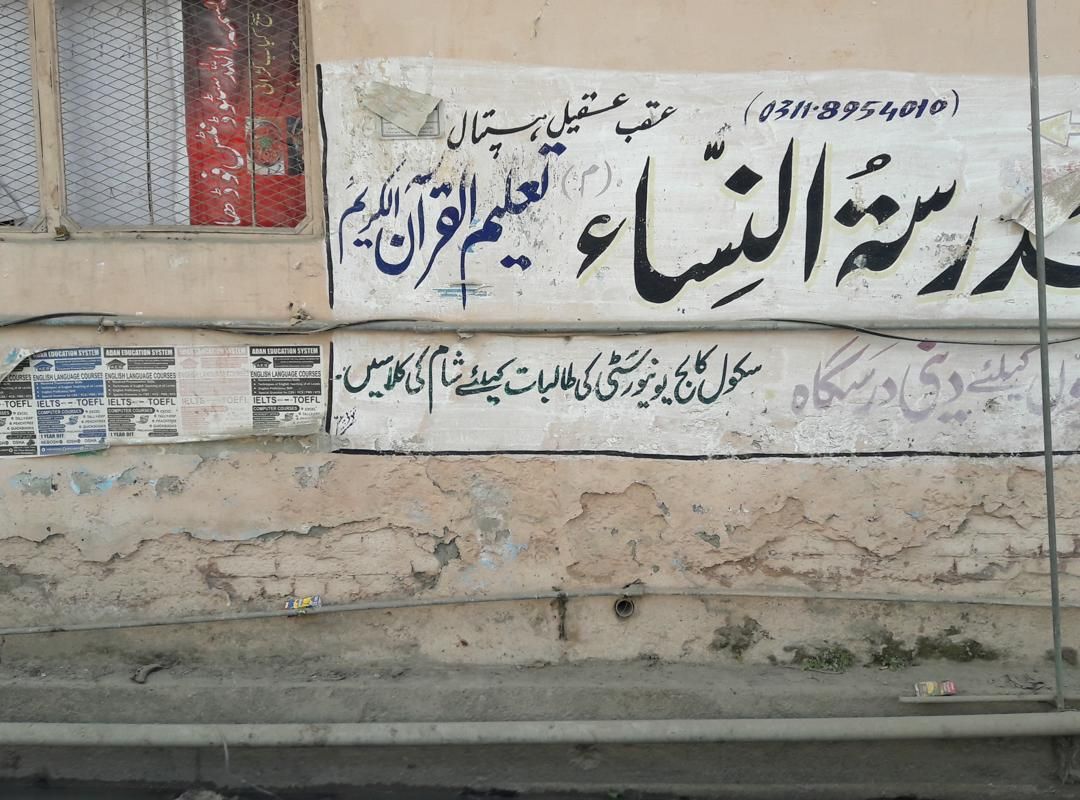

Supplement: Supplemental Information 2 [file peerj-cs-07-717-s002.zip › Testing Dataset/93.jpg]

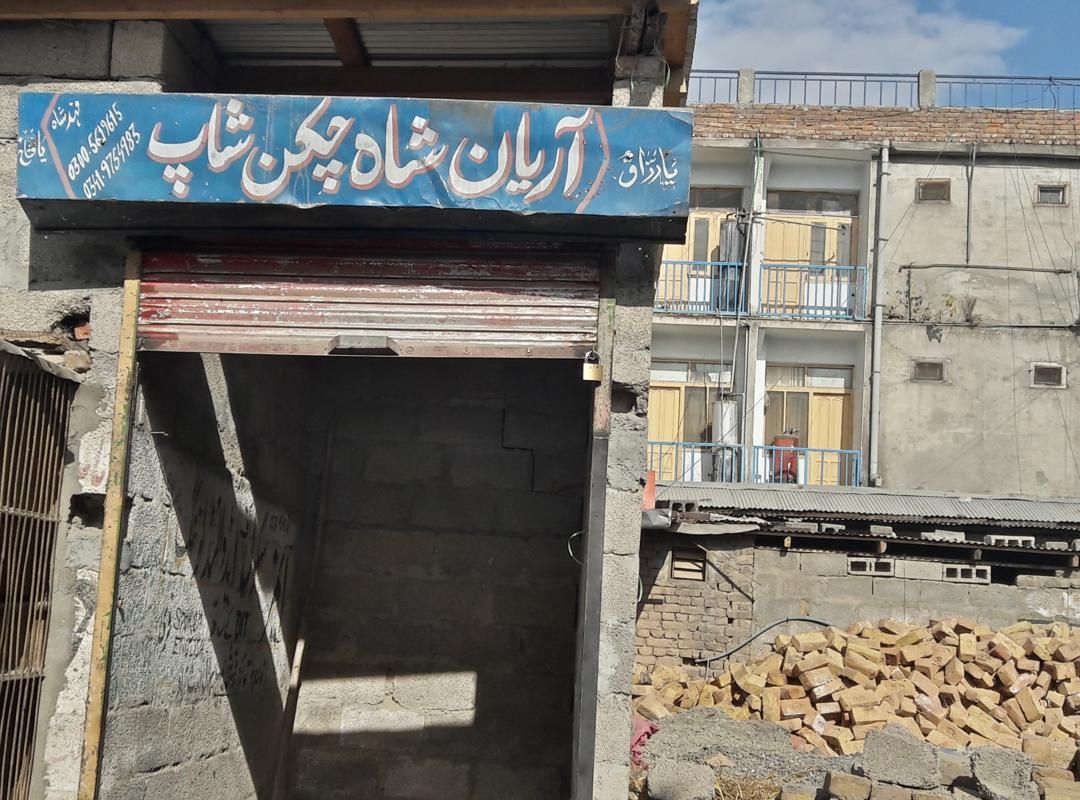

Supplement: Supplemental Information 2 [file peerj-cs-07-717-s002.zip › Testing Dataset/94.jpg]

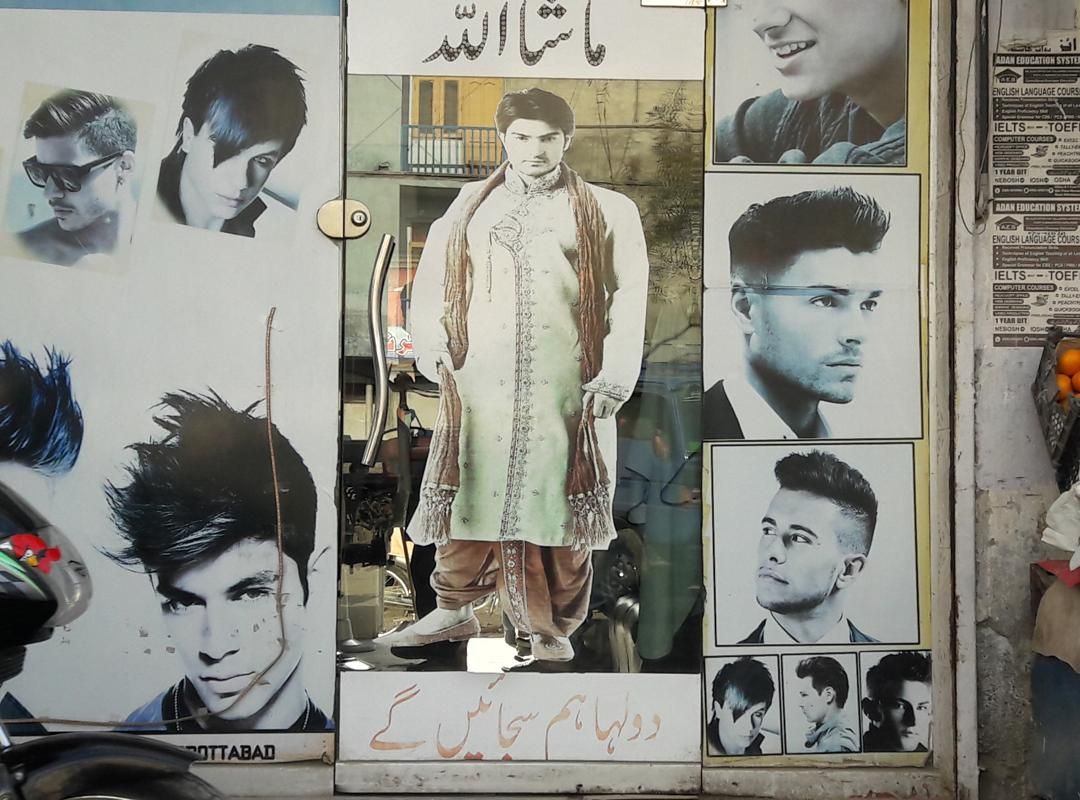

Supplement: Supplemental Information 2 [file peerj-cs-07-717-s002.zip › Testing Dataset/95.jpg]

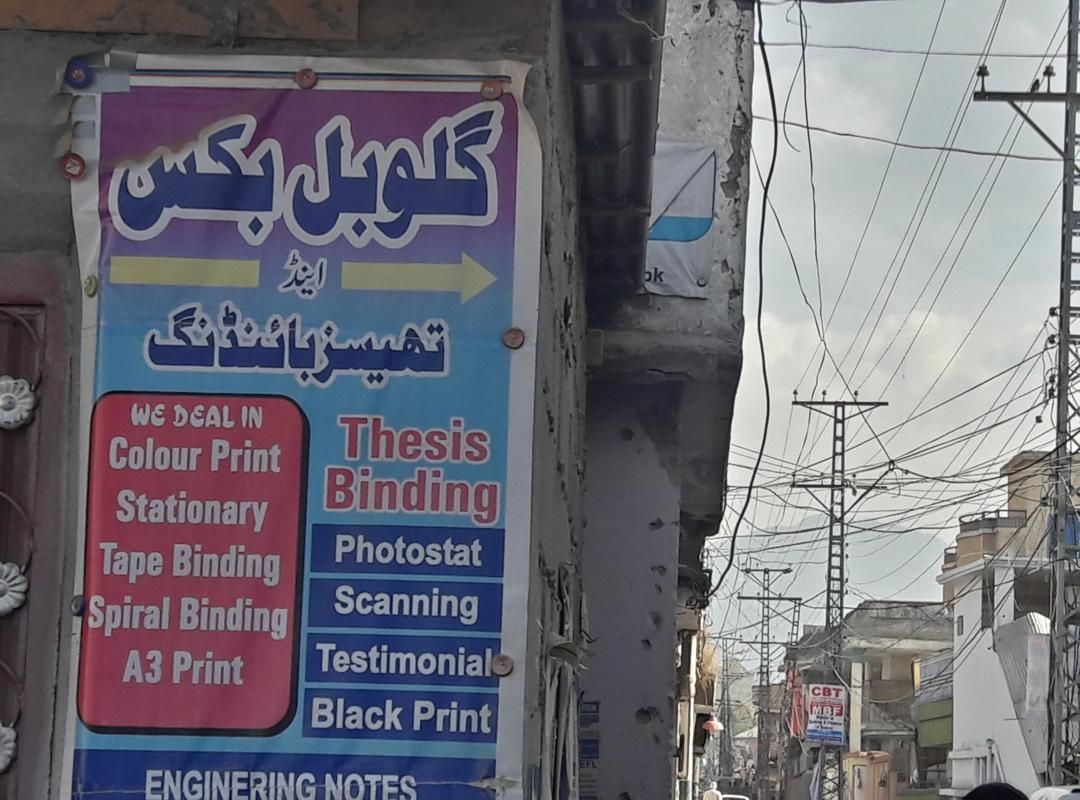

Supplement: Supplemental Information 2 [file peerj-cs-07-717-s002.zip › Testing Dataset/96.jpg]

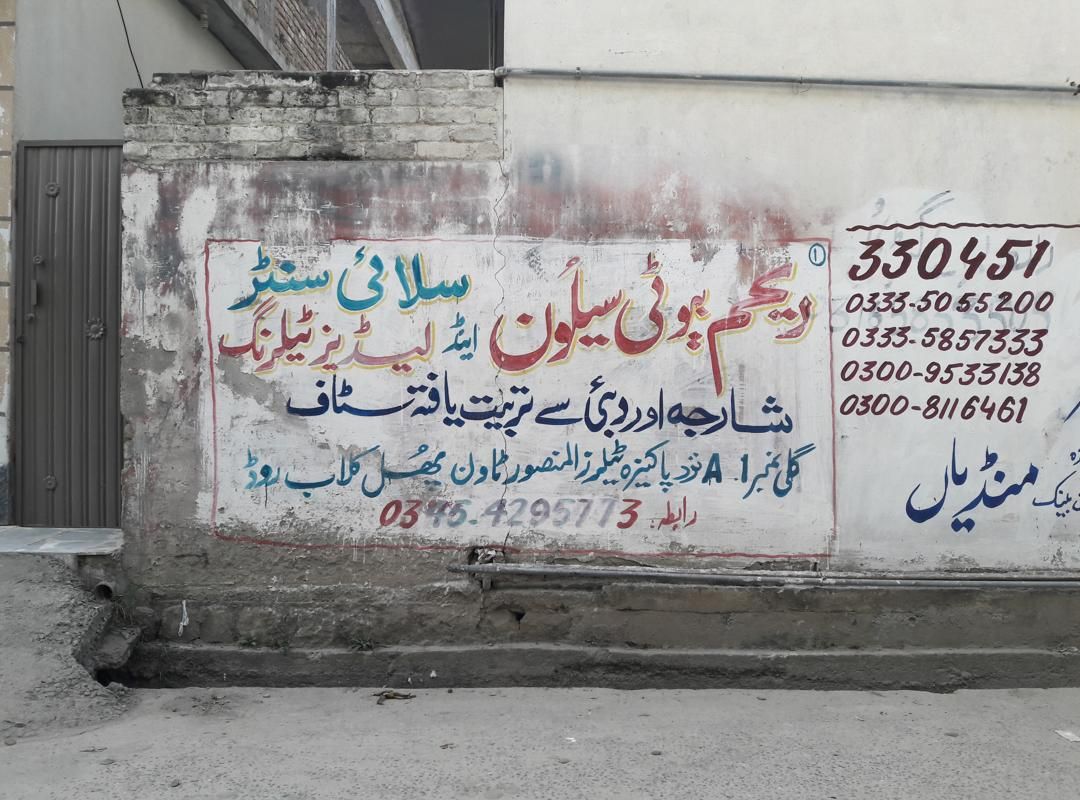

Supplement: Supplemental Information 2 [file peerj-cs-07-717-s002.zip › Testing Dataset/97.jpg]

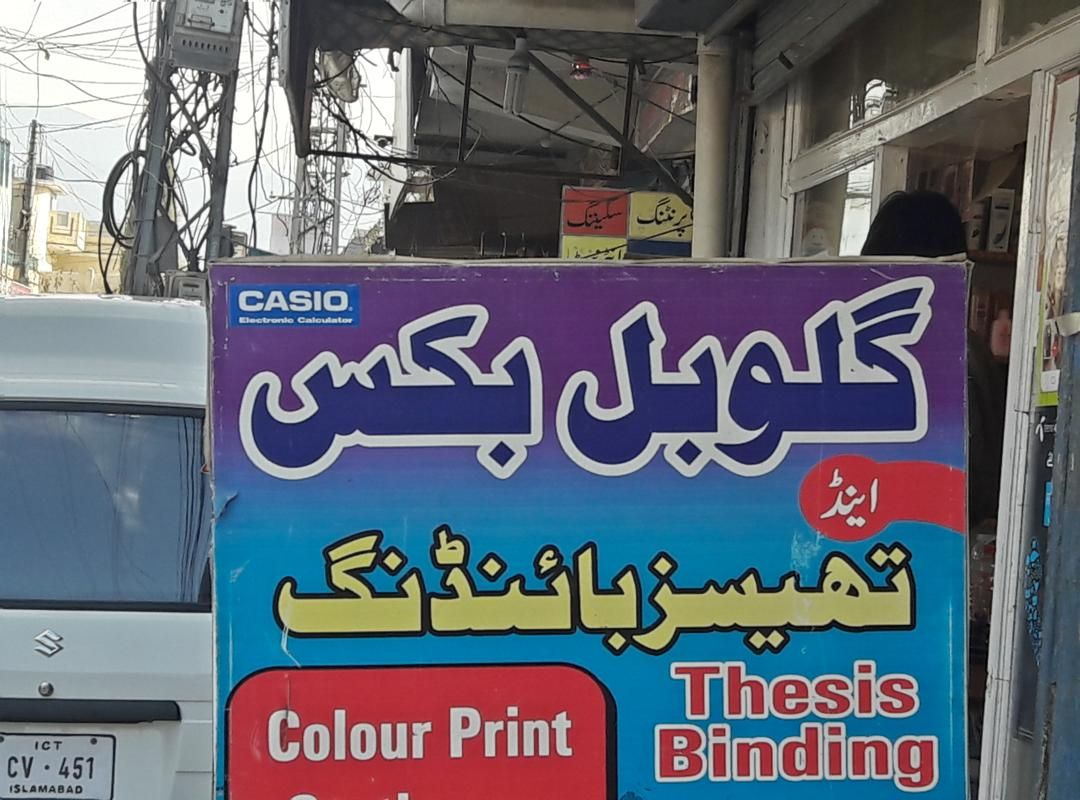

Supplement: Supplemental Information 2 [file peerj-cs-07-717-s002.zip › Testing Dataset/98.jpg]

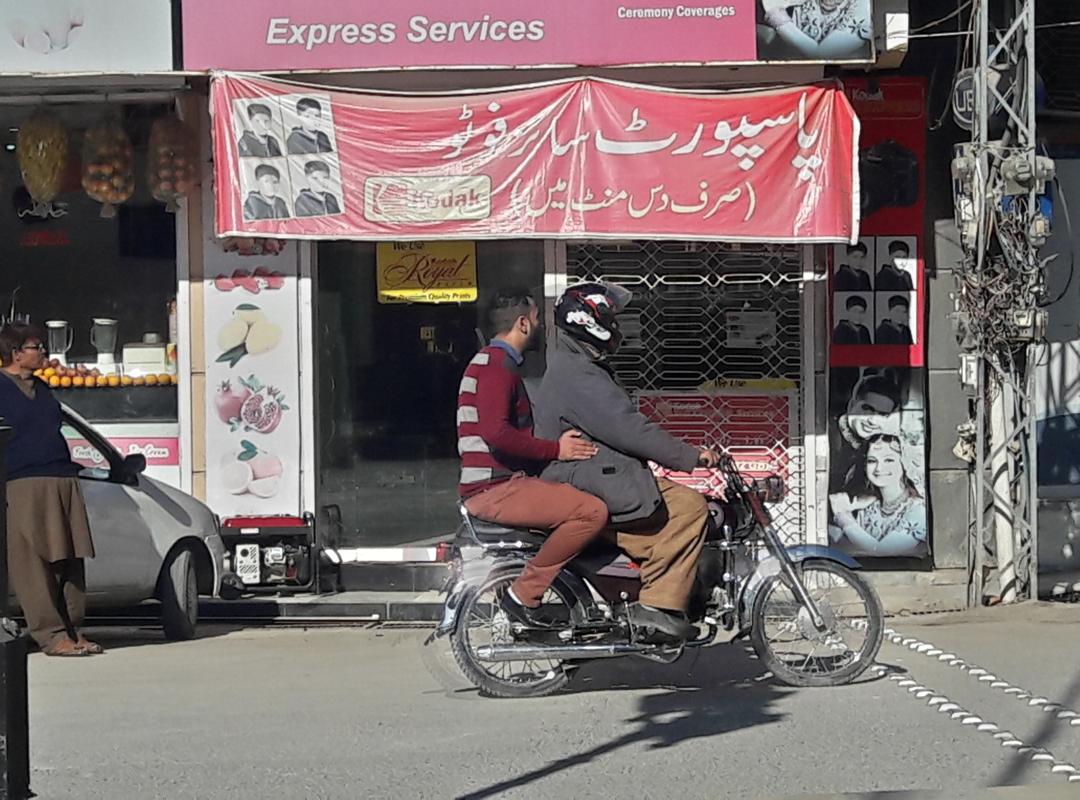

Supplement: Supplemental Information 2 [file peerj-cs-07-717-s002.zip › Testing Dataset/99.jpg]
